# Sample size calculation for training ensemble machine learning models on health data

**DOI:** 10.1016/j.patter.2026.101498

**Published:** 2026-03-26

**Authors:** Nicholas Mitsakakis, Dan Liu, Thomas Walters, Khaled El Emam

**Affiliations:** 1 CHEO Research Institute, Ottawa, ON, Canada; 2 School of Epidemiology and Public Health, Faculty of Medicine, University of Ottawa, Ottawa, ON, Canada; 3 Division of Gastroenterology, Hospital for Sick Children, Toronto, ON, Canada

**Keywords:** sample size calculation

## Abstract

Health research studies often suffer from small sample sizes, and training machine learning (ML) models requires large datasets. There is a dearth of literature on determining the adequate sample size for using ML models. We developed an empirically derived sample size calculator for ensemble ML models: random forests and two gradient-boosted decision trees (light gradient boosting machine [LGBM] and extreme gradient boosting [XGBoost]). This predicts the sample size required to achieve a pre-defined level of prognostic performance with a certain probability. Prognostic performance is defined as the sample area under the ROC curve (ROC-AUC) relative to the optimal model trained on the full (population) dataset. Our calculator’s accuracy was compared to three common heuristics and a statistical approach to sample size calculation. For example, the median relative error sample size prediction was 25% to achieve 85% of the optimal performance with 90% certainty for LGBM. Our model has significantly better accuracy than other methods for tree-based ensemble ML models.

## Highlights

• A conceptual model for thinking about power in prediction models has been developed
• A sample size estimator for ensemble tree-based ML models has been fitted
• The estimator is significantly more accurate than previously proposed methods
• The estimator can be used for sample size calculations for clinical prediction models

## The bigger picture

Machine learning (ML) is transforming healthcare by enabling predictive models that can guide clinical decisions, improve patient outcomes, and optimize resource allocation. Most clinical ML predictive studies, however, overlook a critical design element: determining the quantity of data needed to train a reliable model. Without adequate sample size planning, studies risk producing unstable models that fail to generalize, wasting resources and potentially leading to erroneous conclusions. This gap is especially concerning as ML becomes integral to regulatory submissions and clinical practice.

Our work introduces an empirically derived sample size calculator for ensemble ML models—random forests and gradient-boosted trees—trained on tabular health data. By modeling the relationship between sample size and predictive performance across diverse real-world datasets, we provide a practical tool that moves beyond outdated heuristics and inappropriately used statistical formulas, offering a method suitable for ML model complexity. This framework can improve study design, enhance transparency and reproducibility, and inform policy and reporting standards. Looking ahead, the methodology can be adapted to other domains where ML is applied to structured data, such as finance, education, and public policy. Ultimately, better sample size planning will lead to more trustworthy AI systems, fostering innovation while safeguarding patient and societal interests.

## Teaser

Most clinical prediction modeling studies using machine learning models do not estimate sample size. A conceptual model for thinking about power for machine learning models is presented in this study, and an estimator has been fitted for sample size calculations during study design.

## Introduction

Clinical research studies often have small dataset sizes.^1^ Conducting a study with a sample size that is deemed to be too small can lead to model instability^2^ especially of predictor effects,^3^ as well as overfitting and an inability to generalize predictions to unseen data^4^^,^^5^ even under ideal conditions (e.g., no data shift or drift). This can mean that the study is unable to meet its goals and objectives, and the resources and patient burden expended in collecting the data may not produce meaningful results, which is wasteful. In addition, the ethics of enrolling patients in studies that will not answer the research question(s) are tenuous.^6^ Sample size determination during study design is therefore essential to ensure that studies have sufficient observations to adequately answer the research question.

When the study employs traditional statistical methodologies, sample size calculation can be done using well-established methods.^7^ However, sample size calculation methods have not been established for machine learning (ML) models, which are increasingly applied in clinical research studies.^8^^,^^9^ While it is acknowledged that ML methods require large datasets to achieve optimal predictive performance,^4^^,^^10^ it is unclear what the exact sample size should be to give such optimal and stable model performance and whether the sample size requirements vary by ML method. This poses a significant problem for researchers.

Previous studies that examined ML sample size used existing collected data to fit a power curve and then used that to estimate performance improvements if additional data are collected or additional samples are annotated.^11^^,^^12^ However, this would not be applicable before data collection has started (i.e., during study design). Others make strong assumptions about the data, for example, normality of the predictor variables,^13^^,^^14^^,^^15^^,^^16^ which would not likely be met in many health datasets. While sample size estimation studies have been performed for image data^17^^,^^18^ and spectroscopy data,^19^ these data modalities are not applicable to our context, which is tabular health data. One study examined the impact of sample size and feature selection on random forests; however, in that case, the concept of power was not defined in terms of prognostic performance.^20^ Another study on sample size reported optimism, which is how well the model performed in the training data in comparison to the test data.^4^ In addition, many of these studies (1) did not tune the ML models, and therefore, it is not clear to what extent their results can be applied to the more practical scenario where model tuning is applied, and (2) examined a small range of sample sizes. More details on these studies are included in the supplemental information.

The vast majority of prognostic clinical research studies utilizing ML methods do not report a sample size calculation^3^^,^^9^^,^^21^^,^^22^ and very often rely on convenience sample sizes of available data, avoiding any discussion about the sufficiency of the data for developing an accurate and stable model. Some ML studies rely on precedents for sample size determination, which by themselves oftentimes cannot be justified. Alternatively, oversimplified “rule of thumb” practices borrowed from the statistical literature are often used, such as the “10 events per predictor,”^23^^,^^24^ “15 events per variable” (often used in prediction models^25^), and “300 events per variable.”^4^ The former two are consistent with the median number of 12.5 events per predictor found in the literature.^9^

Approaches found in the statistical literature may be used. For example, one strategy^26^ for calculating the required sample size relies on mathematical formulas and empirical approximations^23^ that are applicable to statistical models, such as linear, logistic, or Cox regression. These formulas and calculations are not necessarily relevant for general ML models because they have significant differences from traditional statistical regression methods in the way they work and the complexity of the relationships that they can capture. However, these methods are still used to estimate the sample size for ML models, despite having potentially nontrivial errors.^27^^,^^28^

Finally, there are methodologies for the estimation of the required sample size for studies aiming to clinically validate previously trained and developed ML models.^29^ These approaches assume the existence of already trained ML models, and they focus on the effect of the sample size of new data needed for precisely estimating measures of the model’s performance as part of the validation objective. As such, their goal is distinct from estimating sample size for developing and training a new ML model, and therefore, they cannot be applied to our objectives.

In this study, we develop a sample size calculator with the following characteristics: sample size calculations that would be performed *a priori*, before data collection, (1) for training an ML model and (2) for ensemble binary classification ML modeling methods that will be trained on a structured tabular dataset for prognostic purposes and (3) that would perform better than the available heuristic and statistical methods.

The specific ensemble ML methods that we consider are random forests and gradient-boosted decision trees.

## Methods

Our methodology consists of performing a retrospective simulation to model the relationship between sample size and ML model performance.

### Simulation design

**Figure 1:**
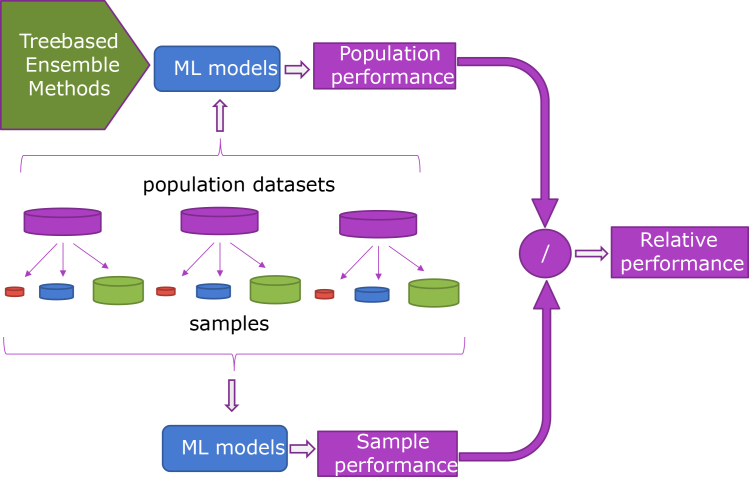
Diagram illustrating the process of our computational experiments

We used large real datasets as “population” datasets. Then, we trained, tuned, and assessed three tree-based ensemble ML models, obtaining the “optimal” performance for these classifiers on the population datasets. Subsequently, we sampled data with varying sizes from the population datasets and used them for training, tuning, and testing the ML models. The performance of these sample-trained models was then compared with the “optimal” performance, indicating the impact of a smaller sample size relative to the optimal performance. An overview of the overall process is shown in Figure 1.

#### Datasets

We used 13 large real-world health datasets^30^^,^^31^^,^^32^^,^^33^^,^^34^^,^^35^^,^^36^^,^^37^^,^^38^ to represent different populations. These datasets covered multiple domains and settings, including public health, health surveys, hospital discharge, intensive care unit (ICU) visits, adverse events, and specific population registries. The heterogeneity of complexity in these datasets was deliberate to enable generalization to a broader set of predictive clinical research scenarios. Being real datasets, they embodied a realistic mix of continuous and categorical variables, non-linearities, correlational structure, measurement error, possible noise variables, potential labeling errors for the outcome, and various levels of outcome imbalance. These are all factors that are known to have an impact on ML model performance and influence the relationship between sample size and performance.^39^^,^^40^^,^^41^

A summary of these datasets is provided in Table 1, with more details about each dataset provided in Tables S2–S14. For each dataset, a binary classification model was defined, and these are described in the supplemental information. The number of variables used in each classification model is shown in the last column of Table 1. In addition, the table shows the original number of observations and the number of observations after removing those with any missing values on the outcome variable.

**Table 1:** A description of the complete datasets used in the analyses

Dataset (dataset label)	Description of dataset	No. observations (original)	No. observations^a^	Variables used in analysis
COVID^30^ (COVID)	COVID-19 health records of Canadians collected by Esri Canada	1,384,881	745,623	7
Canadian Community Health Survey^31^ (CCHS)	a pooled version of survey data across multiple years that gathers health information for Canadian population	904,813	752,472	8
COVID Survival^32^ (Nexoid)	a secondary web-based survey dataset concerning COVID-19 survival prediction collected by the Nexoid company in London, UK	968,408	968,394	19
FDA Adverse Event Reporting System^33^ (FAERS)	adverse event and medication error reports submitted to FDA	881,204	251,409	7
Texas Inpatient Data^34^ (TEXAS)	discharges from Texas hospitals	745,999	745,997	11
Washington State Hospital Discharge^35^ (WASHINGTON2007)	hospital discharge information from the HCUP state inpatient database for 2007	644,902	644,901	8
Basic Stand Alone (BSA) Inpatient Claims^36^	claim-level information from 2008 Medicare inpatient claims	588,415	588,415	6
Washington State Hospital Discharge^35^ (WASHINGTON2008)	hospital discharge information from the HCUP state inpatient database for 2008	652,340	652,340	18
California Hospital Discharge^35^ (CALIFORNIA2007)	hospital discharge information from the HCUP state inpatient database for 2007	4,016,573	4,016,573	16
Florida Hospital Discharge^35^ (FLORIDA2007)	hospital discharge information from the HCUP state inpatient database for 2007	2,327,563	2,327,563	12
New York Hospital Discharge^35^ (NEWYORK2007)	hospital discharge information from the HCUP state inpatient database for 2007	4,666,541	4,666,541	14
Better Outcomes Registry & Network^37^ (BORN)	a registry that contains comprehensive perinatal, newborn, and child information in Ontario	963,083	963,083	18
Medical Information Mart for Intensive Care III^38^ (MIMIC-III)	health-related information for patients who stayed in critical care units of the Beth Israel Deaconess Medical Center between 2001 and 2012	540,482	540,482	10

a After data transformation/removing missing values on the outcome variable only.

#### Evaluation approach

Every population dataset was randomly split into 70% training and 30% test datasets. For each population dataset, the same testing partition was used to evaluate all models trained using that dataset. This ensured that the results from all models, irrespective of the sample size that was used to train them and irrespective of the type of ML algorithm, would be evaluated on the same test set, which allows consistent comparability across all models.

The area under the ROC curve (ROC-AUC) was used to assess the performance of the binary classification models. ROC-AUC is a measure of discrimination, which is an important aspect of the performance of an ML model, and it is widely used in similar studies because it can be used to compare performance across different studies and datasets. Other measures, such as the Brier score (measuring both discrimination and calibration), do not share similar properties, as they depend on the particular dataset, and they can mainly be used for comparing the performance of models trained on the same dataset. For instance, the Brier score depends inherently on the prevalence of the outcome, posing interpretation challenges when used across different datasets.^42^^,^^43^

#### Sampling from population data

Samples were drawn from each of the training partitions, using different sample sizes. The sizes of the samples were based on a geometric series. Define *b* ∼ *N*(*μ* = 1.5,*σ*^2^ = 0.005^2^), and the series would be *n*_*i*_ = [*b*^*i*+9^],*i* = 1, …,23, where [*x*] denotes rounding to the closest integer to *x*. The series would be stopped if a value exceeded the training partition size for a particular dataset.

The justification behind the choice of geometric series is based on the assumption and expectation that changes in a model’s performance (on ROC-AUC) diminish as the sample size increases (i.e., the performance will plateau). 100 series were generated for each dataset, and for each value in a series, a random sample of that size was drawn from each training dataset. Therefore, for each dataset, there were 2,300 models trained with varying sample sizes. Each series is randomized so that we can have sample sizes across the range rather than at fixed points, ensuring smoothness in the modeling described below.

### ML methods

Our investigation focuses on tree-based ensemble ML methods: random forests and gradient boosting decision trees (LightGBM and XGBoost). Tree-based models are the most common type of ML prognostic methods used in clinical research^9^; they perform better than linear models, such as logistic regression,^44^^,^^45^^,^^46^^,^^47^^,^^48^ and have also been found to perform better than deep learning models on tabular datasets.^49^^,^^50^

Model tuning used 5-fold cross-validation. All models were tuned using Bayesian optimization.^51^ The range for the tuning parameters, specific for each model, was previously suggested^52^^,^^53^^,^^54^ and is detailed in Table S1. High-cardinality variables were converted to embeddings^55^ using a scheme similar to target encoding. The sdgm R package that implements this functionality is available online.^56^

### Model specification for observed sample size

#### Concept of certainty curves

Previous studies assessed the pseudo-power of an ML model by examining the relationships between predictors and the outcome^20^ or used the power of the ROC-AUC calculation itself.^39^ We sought to develop a direct approach that is more easily interpretable by analysts when designing their studies.

Here, we describe a new approach that can be used for measuring and assessing the adequacy of the size of a dataset to be used for training ML models. The adequacy is characterized based on the estimated performance of the model(s) training on the particular dataset, as it compares with the hypothetical maximum (or optimal) performance of the model(s) when applied to the population from which the dataset is sampled. We are making the assumption that the performance of the model is monotonically improving on average as the sample size increases, and it reaches its maximum under the population from which the dataset is sampled.

Since the estimated performance on the dataset is stochastic, we adopt a probabilistic framework. We describe certainty curves whereby the analyst already knows which ML modeling technique will be used and wishes to determine the sample size to use for training that ML model.

While the description below is for ROC-AUC, similar reasoning applies to other model performance measures that can be used across different datasets and studies. Under this setting, certainty curve *C*(*n*) is a mathematical function from *N*∗ to (0,1), given by

(Equation 1) $$C\left(n\right)={\mathrm{Pr}}_{\left|S\right|=n,S\subset P\;}\left(AUC\left(S\right)\geq t_{s}|AUC\left(P\right)\geq t_{p}\right)\text{,}$$

where *S* denotes a random sample from a population dataset, |*S*| is the size of *S*, *ROC-AUC*(*X*) denotes the value of the ROC-AUC for the model trained on dataset *X*, *t*_*s*_ and *t*_*p*_ are threshold parameters for ROC-AUC values for models trained on a sample and on the population, respectively, and *P* is the population.

*C*(*n*) is giving the probability for the specific ML model to have ROC-AUC performance of at least equal to *t*_*s*_ when trained on a dataset *S*, which has sample size *n* from a population *P*, given that the model gives a ROC-AUC at least equal to *t*_*p*_ when trained on this population *P*. In practice, *t*_*s*_ ≤ *t*_*p*_. For example, for values *t*_*s*_ = 0.75 and *t*_*p*_ = 0.8 and assuming that the modeling method is random forest, *C*(*n*) gives the probability that a random forest model trained on a sample *S* with size *n*, from a specific population *P*, will have performance with a ROC-AUC ≥ 0.75, assuming that the model’s performance based on the population is given by a ROC-AUC ≥ 0.8.

The performance of a trained ML model generally improves with the increasing size of the training data. Therefore, here we assume that the certainty curve function *C*(*n*) is an increasing function (however, not necessarily *strictly* increasing).

Once *C*(*n*) is available for different values of *n*, or as a curve, one can use this to “solve” for the value of *n*, given a specific value of *C*(*n*). For instance, based on analogy with statistical power, we can obtain the sample size that gives 80% certainty by empirically solving *n*∗ = *C*^−1^(0.8). Since the certainty curve may not have a unique mapping between an input and an output value, we define the pseudo-inverse of *C*(*n*) to be *C*^−1^(*c*): = min{*n*: *C*(*n*) = *c*}, i.e., for a certainty value *c* (e.g., 0.8), *C*^−1^(*c*) is equal to the smallest *n* that gives *C*(*n*) = *c*.

The concept of certainty curves is inspired by the traditional statistical concept of power, used in hypothesis testing. Power is defined as the probability of “detecting a signal” (often expressed by the rejection of the null hypothesis using a statistical test), given that the signal exists, and it has a specific magnitude (expressed by the assumption that an alternative hypothesis is true). The analogy of our method with the definition of statistical power is as follows: the assumption “*ROC-AUC*(*P*) ≥ *t*_*p*_” plays the role of the alternative hypothesis being true, while the event “*ROC-AUC*(*S*) ≥ *t*_*s*_” plays the role of the detection of the “signal” of interest in a sample, in our case a model trained on a dataset of size *n* with sufficient performance (*ROC-AUC*(*S*) ≥ *t*_*s*_).

While our framework is described under the general case of independent values for *t*_*p*_ and *t*_*s*_ thresholds, in our evaluation, we adopt the approach where *t*_*s*_ is given as a fraction of *t*_*p*_, i.e., *t*_*s*_ = *λ*·*t*_*p*_. Under that setting, the required sample size refers to the capacity of a specific modeling method to be used for training a model that achieves satisfactory performance *relevant to the maximum performance* (based on the population data). For example, using *λ* = 0.85, assuming that at a population level, the performance of ROC-AUC ≥ 0.8 (i.e., *t*_*p*_ = 0.8), the performance of a model developed using a sample of the data is deemed satisfactory if ROC-AUC ≥ *t*_*s*_, where *t*_*s*_ = *λ*·*t*_*p*_ = 0.85 × 0.8 = 0.68.

#### Calculating the “observed” sample size

For each of the *K* datasets (*K* = 13) used for the simulation, we can obtain the observed sample size *n*∗ at a given level of certainty by first using dataset-specific univariable logistic regression models for modeling the certainty given in Equation 1, the outcome being whether the ROC-AUC values exceed the *t*_*s*_ threshold or not. Let logit(*C*_*obs*_(*n*)) = *a* + *bln*(*n*); then, a fitted model can be used for determining the sample size *n*∗ required for obtaining a sufficient level of certainty *C*∗, for a given dataset, i.e., *n*∗ = min{*n*: *C*_*obs*_(*n*) ≥ *C*∗}. This is considered to be the true sample size required to achieve a certain certainty level. A logistic regression model is fitted on the 2,300 observations per dataset.

Using the logistic regression model allows us to smooth the relationship between the outcome and the sample size and ensure that it is monotonic. The *a* and *b* parameters are not expected to be the same across different datasets and are used to determine the *n*∗ values used for performance evaluation.

#### A calculator of sample size

The sample size calculator uses a prediction model for estimating the certainty curve as a function of various dataset characteristics. Based on the certainty curve, the required sample size is obtained.

In our experiments, we have *P*_*k*_,*k* = 1, …,*K* real datasets, *n*_*i*_ sample sizes, *S*_*ijk*_ ⊂ *P*_*k*_ sample sets from the population data, where *i* refers to the sequence in the geometric series. The value of *j* pertains to the series, with *j* = 1, …,100. Then, for each value of *i*,*j*,*k*, we obtain a binary variable indicating whether the condition *ROC-AUC*(*S*_*ijk*_) ≥ *t*_*s*_ is met based on our experiments. More specifically, we set

(Equation 2) $$y_{ijk^{*}}=I\left(AUC\left(S_{ijk^{*}}\right)\geq t_{s}\right),j=1,\ldots ,100,k^{*}\in \left\{k:AUC\left(P_{k}\right)\geq t_{p}\right\}\text{.}$$

Then, the tuple $\left\{y_{ijk^{*}},\left|S_{ijk^{*}}\right|\right\}$ can be used for fitting a predictive calculator model.

Additional predictors in the calculator model relate to characteristics of the datasets that have previously been found to affect the sample size requirements. These are the amount of imbalance between the two classes of the binary outcome, the information provided by each predictor (measured by entropy), and the number of predictor parameters (or degrees of freedom [*df*]).^23^^,^^25^ They are captured by the following variables used as predictors in the calculator.

• The imbalance factor (*IF*) is given by *IF* = max{*prev*/(1 − *prev*), (1 − *prev*)/*prev*}, where *prev* is the prevalence of the outcome of interest. For example, if the outcome is mortality and 4% of the individuals in the dataset have died, then *prev* = 0.04 and *IF* = 24.
• The mean standardized entropy (*entr*) is given by the average value of standardized entropy across all predictors in the model. For any individual variable that has been discretized, it is defined as

(Equation 3) $$-\frac{\sum_{i=1}^{z}p_{i}\;\log_{2}\left(p_{i}\right)}{\log_{2}\left(z\right)}\text{,}$$

where *z* is the total number of categories in that variable and *p*_*i*_ is the proportion of observations in category *i*. For continuous variables with fewer than 10 unique values, it is treated as categorical. If a continuous variable has more than 10 categories, then it is categorized using the Sturges method.

• *df* parameters are based on the number of predictors, where each predictor contributes a value of 1 if it is numerical or binary and a value of *l* if it is categorical with (*l* + 1) levels, and summed across all predictors. For example, if a dataset had two variables, a binary one and a categorical one indicating the highest degree obtained, and there were six categories, then the value would be *df* = 1 + 5 = 6.

The values for the population datasets are summarized in Table 2. All the values across *i*, *j*, and *k* were used for fitting the calculator model as

(Equation 4) y∼n+log(IF)+entr+log(df).

**Table 2:** Characteristics of the population datasets

Dataset	Imbalance factor	Mean standardized entropy	Degrees of freedom
COVID	66.59	0.79	47
CCHS	1.77	0.87	11
Nexoid	1.55	0.34	31
FAERS	9.06	0.62	14,834
TEXAS	1.47	0.67	345
WASHINGTON2007	1.04	0.69	11,000
BSA	1.34	0.91	400
WASHINGTON2008	2.93	0.65	11,127
CALIFORNIA2007	1.18	0.68	9,330
FLORIDA2007	1.53	0.69	45,145
NEWYORK2007	1.62	0.75	19,669
BORN	13.27	0.32	27
MIMIC-III	3.76	0.28	46

The model in Equation 4 can then be used for estimating the probability values that define the certainty curve function *C*(*n*) for any value of *n*. Each model had 29,900 observations for fitting.

These data are clustered, with the dataset constituting the clustering factor. While modeling methods for clustered data would be the natural choice, their benefits when prediction (and not explanation) is the modeling objective are questionable.^57^ We therefore chose to use LGBM as the modeling method, given its often-found superior performance. Note that this is a second and different use of LGBM to fit the sample size calculator.

Using this estimation method and the results from our simulation, we can generate different certainty curves according to Equation 4 for each of the three ML modeling methods used in our simulation and each of the *λ* values. A researcher can choose the right certainty curve for estimating the required sample size, given that a specific modeling method and *λ* value have been chosen *a priori*.

Assuming a model fitted for a specific ML method *m* and using a specific value for *λ*, for a future study assuming data with known values for characteristics *IF*, *entr*, and *df*, the estimated certainty for a given sample size *n* is denoted by *C*_*λ,m*_(*IF*, *entr*, *df*, *n*). Given that, the required sample size for obtaining a certainty value of at least *C*∗ is given by $\hat{n}$ = min{*n*: *C*_*λ,m*_(*IF*, *entr*, *df*, *n*) ≥ *C*∗}.

The pseudocode for the sample size calculator is provided in the supplemental information.

#### Performance measure for sample size estimation models

We assess the performance of the certainty curve method by comparing the observed and predicted values of the required sample size for the *K* datasets, where predicted values, denoted by $\hat{n}$, were obtained using leave-one-dataset-out (i.e., the predicted sample size for a particular sample dataset was obtained by using a model trained on the remaining *K* − 1 dataset samples).

For each of the *K* datasets (*K* = 13) used for the simulation, the observed sample size *n*∗ was obtained by first constructing the observed certainty curve *C*_*obs*_(*n*) and then selecting the sample size *n*∗ that corresponds to the desired certainty value from the curve. This is considered to be the true sample size required to achieve the desired certainty level for a particular dataset.

Because we use leave-one-dataset-out, no observations used to fit the logistic regression model are used in training a sample size estimation model (see Equation 4) when that dataset is taken out. This ensures that no data leakage occurs.

The performance of a statistical estimation method,^26^ as well as three other heuristic approaches, 10 events per variable,^23^^,^^24^ 300 events per variable,^4^ and 15 events per variable,^25^ were also calculated as comparators. At the time of writing, these four methods are the only ones currently available for analysts to use for sample size estimation.

One metric that provides a measure of bias and the direction of bias is the median relative error (mRE), defined as

(Equation 5) $$\text{mRE}=100\times \text{median}\left(\frac{{\hat{n}}_{k^{*}}-n_{k^{*}}^{*}}{n_{k^{*}}^{*}}\right)\text{.}$$

We also use the median of the absolute log quotient error (mALQE) between the observed and predicted required sample sizes. That is,

(Equation 6) $$\text{mALQE}=100\times \left(\exp\left(\text{median}\left|\log\left(\frac{{\hat{n}}_{k^{*}}}{n_{k^{*}}^{*}}\right)\right|\right)-1\right)\text{.}$$

It can be interpreted as the median symmetric absolute percentage deviation between the observed and predicted values. Values close to 0 indicate a better fit. This measure is consistent with measures discussed previously,^58^^,^^59^^,^^60^ and it is based on the ratio between the observed and predicted sample sizes. It returns the same error when predicted and observed values are swapped. It is therefore symmetrical, and this is important so that under- and overestimation do not cancel each other out when aggregating across the whole dataset (as with mRE). By exponentiating the mALQE, we obtained a value of >1, playing the role of the ratio. By subtracting 1, we obtain a measure where 0 indicates a perfect fit. However, it does not give direction of bias, which the mRE provides.

## Results

**Figure 2:**
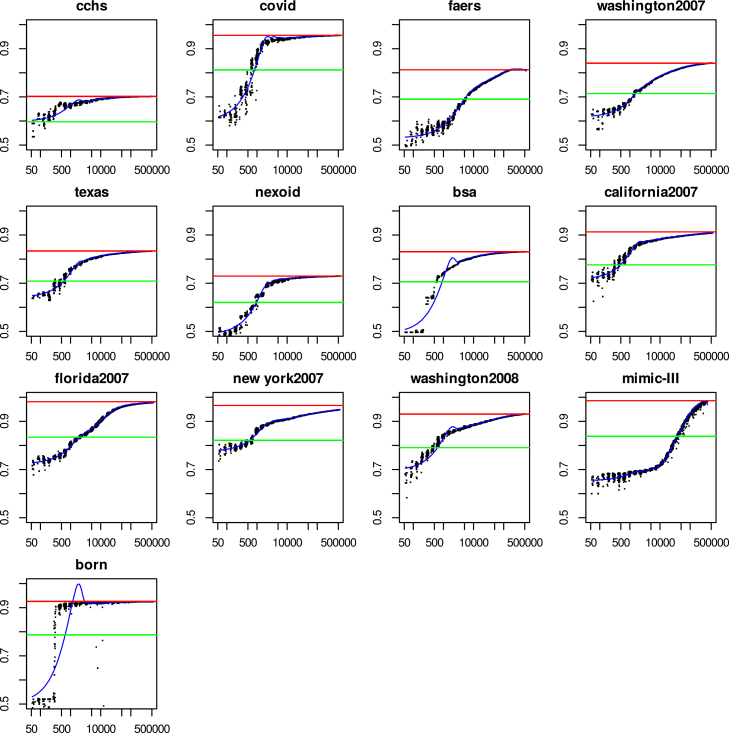
ROC-AUC values against sample size for LGBM models The red (top horizontal) line indicates population ROC-AUC and the green (bottom horizontal) line indicates 0.85∗(population ROC-AUC).

**Figure 3:**
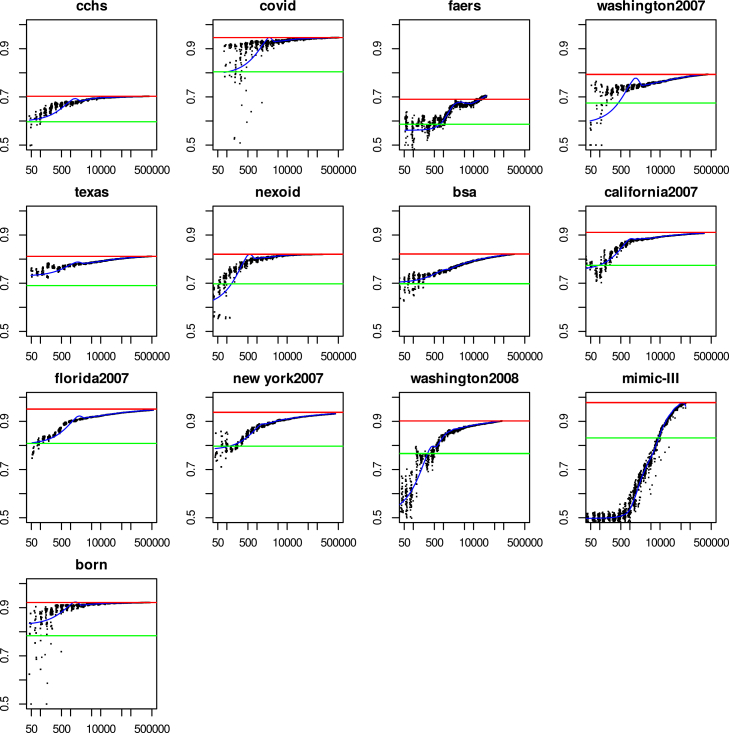
ROC-AUC values against sample size for RF models The red (top horizontal) line indicates population ROC-AUC and the green (bottom horizontal) line indicates 0.85∗(population ROC-AUC). RF, random forest.

**Figure 4:**
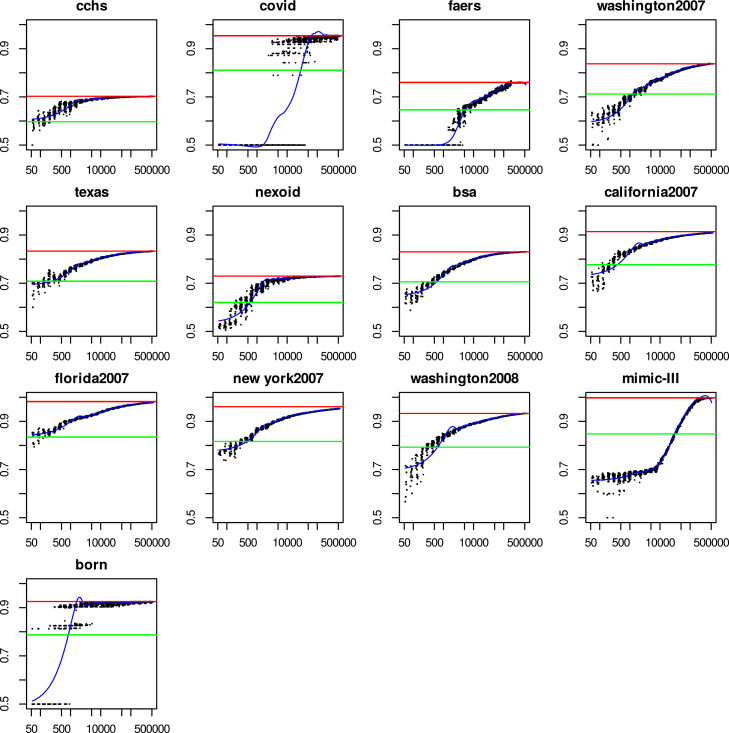
ROC-AUC values against sample size for XGB models The red (top horizontal) line indicates population ROC-AUC and the green (bottom horizontal) line indicates 0.85∗(population ROC-AUC). XGB, XGBoost.

The performance of the three ensemble models trained on the samples was plotted against the sample size for each dataset separately; large variability in the trend between sample size and ROC-AUC values across different datasets and different modeling methods was observed. For some datasets, a small sample size was sufficient for the model to achieve performance comparable to the population level, while in some other cases, the latter was achieved with only very large sample sizes. Samples from the COVID and Better Outcomes Registry & Network (BORN) datasets seem to approach population-level performance “the fastest” (i.e., requiring the smallest sample sizes), while samples from the WASHINGTON2007 dataset require very large sizes in order to approach population-level performance. Figures 2, 3, and 4 illustrate these results for the three types of ensemble models, where the red horizontal line indicates population-level ROC-AUC and the green line indicates the “target” ROC-AUC, equal to 0.85∗(population ROC-AUC).

The sample size calculator model was fitted to estimate certainty using different values for parameter *λ* (0.8, 0.85, and 0.9). The discrepancy between the predicted and observed required sample sizes at 80% and 90% certainty, measured by the mRE and mALQE, was compared between the proposed certainty curve method and the four other previously proposed methods. The results for the mRE are given in Tables 3 and 4 and for the mALQE in Tables 5 and 6 at 80% and 90% certainty, respectively. In addition to these summary error measures, the comparison of the predicted and observed required sample sizes for the 13 datasets is depicted in the scatterplots in Figures S1 and S2.

**Table 3:** Comparison of the percentage median (IQR) relative error using leave-one-dataset-out at 80% certainty between the certainty curve approach and other methods (Riley et al., 300 events per variable, 15 events per variable, and 10 events per variable)

Model	*λ*	Certainty curve (%)	Riley (%)	EPV300 (%)	EPV15 (%)	EPV10 (%)
LGBM	0.80	−66.8 (−88.7, 79.1)	9,012 (4,219, 95,297.5)	142,094.5 (29,570.5, 1,448,657.9)	7,009.9 (1,383.6, 72,338.1)	4,639.9 (889.2, 48,192.1)
LGBM	0.85	16.5 (−62.9, 222.7)	4,270.3 (2,760.9, 57,274.4)	142,094.5 (29,570.5, 1,448,657.9)	7,009.9 (1,383.6, 72,338.1)	4,639.9 (889.2, 48,192.1)
LGBM	0.90	56.2 (−41.2, 1,131)	2,868.2 (1,843.9, 45,661.1)	142,094.5 (29,570.5, 1,448,657.9)	7,009.9 (1,383.6, 72,338.1)	4,639.9 (889.2, 48,192.1)
RF	0.80	−37.4 (−62.5, 23.6)	93,404 (12,797.1, 503,340.6)	732,748.5 (231,330, 4,680,502.3)	36,543.1 (11,472, 233,930.8)	24,328.5 (7,616, 155,920.8)
RF	0.85	8.8 (−25.3, 150.1)	57,824 (6,276, 345,942.8)	732,748.5 (231,330, 4,680,502.3)	36,543.1 (11,472, 233,930.8)	24,328.5 (7,616, 155,920.8)
RF	0.90	130.8 (31.9, 392.5)	38,930 (4,334, 247,243.9)	732,748.5 (231,330, 4,680,502.3)	36,543.1 (11,472, 233,930.8)	24,328.5 (7,616, 155,920.8)
XGB	0.80	−33 (−88.9, 47.3)	19,078.9 (1,339.6, 195,964.9)	134,669.5 (8,221.8, 2,253,523.3)	6,638.9 (316.4, 112,581.5)	4,392.6 (178.2, 7,5021.1)
XGB	0.85	56.7 (−80.7, 312.8)	11,970.5 (590.8, 121,139.6)	134,669.5 (8,221.8, 2,253,523.3)	6,638.9 (316.4, 112,581.5)	4,392.6 (178.2, 7,5021.1)
XGB	0.90	395 (−44, 1070.2)	8,108.9 (368, 86,185.9)	134,669.5 (8,221.8, 2,253,523.3)	6,638.9 (316.4, 112,581.5)	4,392.6 (178.2, 7,5021.1)

**Table 4:** Comparison of the percentage median relative error using leave-one-dataset-out at 90% certainty between the certainty curve approach and other methods (Riley et al., 300 events per variable, 15 events per variable, and 10 events per variable)

Model	*λ*	Certainty curve (%)	Riley (%)	EPV300 (%)	EPV15 (%)	EPV10 (%)
LGBM	0.80	−61.3 (−87.2, 149.2)	9,012 (4,219, 94,946)	142,094 (28,828, 1,448,657)	7,009 (1,346, 72,338)	4,639 (864, 48,192)
LGBM	0.85	25.3 (−60.6, 254.6)	4,270.3 (2,760.9, 57,063)	142,094 (28,828, 1,448,657)	7,009 (1,346, 72,338)	4,639 (864, 48,192)
LGBM	0.90	64.8 (−38, 1,165.2)	2,868.2 (1,843.9, 45,661)	142,094 (28,828, 1,448,657)	7,009 (1,346, 72,338)	4,639 (864, 48,192)
RF	0.80	−28.1 (−59.7, 22.7)	91,072 (12,797, 476,843)	680,402 (231,330, 4,346,173)	33,925 (11,472, 217,214)	22,583 (7,616, 144,776)
RF	0.85	9.3 (−21.1, 166.8)	55,466 (6,276, 327,730)	680,402 (231,330, 4,346,173)	33,925 (11,472, 217,214)	22,583 (7,616, 144,776)
RF	0.90	137.4 (62.2, 444)	36,958 (4,334, 234,225)	680,402 (231,330, 4,346,173)	33,925 (11,472, 217,214)	22,583 (7,616, 144,776)
XGB	0.80	−22.7 (−86.8, 72.3)	19,078.9 (1,311, 193,711)	134,669 (7,528, 2,253,523)	6,638 (281, 112,581)	4,392 (155, 75,021)
XGB	0.85	74.6 (−77.5, 368.6)	11,970.5 (577, 121,139)	134,669 (7,528, 2,253,523)	6,638 (281, 112,581)	4,392 (155, 75,021)
XGB	0.90	471.2 (−34.8, 1136.1)	8,108.9 (358, 86,185)	134,669 (7,528, 2,253,523)	6,638 (281, 112,581)	4,392 (155, 75,021)

**Table 5:** Comparison of the percentage median absolute quotient error using leave-one-dataset-out at 80% certainty between the certainty curve approach and other methods (Riley et al., 300 events per variable, 15 events per variable, and 10 events per variable)

Model	*λ*	Certainty Curve	Riley	EPV300	EPV15	EPV10
LGBM	0.80	482	9,012	142,095	7,010	4,640
LGBM	0.85	222	4,270	142,095	7,010	4,640
LGBM	0.90	345	3,227	142,095	7,010	4,640
RF	0.80	121	93,404	732,749	36,543	24,328
RF	0.85	55	57,824	732,749	36,543	24,328
RF	0.90	202	38,930	732,749	36,543	24,328
XGB	0.80	681	1,9079	134,670	6,639	4,393
XGB	0.85	418	11,971	134,670	6,639	4,393
XGB	0.90	397	8,109	134,670	6,639	4,393

**Table 6:** Comparison of the percentage median absolute quotient error using leave-one-dataset-out at 90% certainty between the certainty curve approach and other methods (Riley et al., 300 events per variable, 15 events per variable, and 10 events per variable)

Model	*λ*	Certainty curve	Riley	EPV300	EPV15	EPV10
LGBM	0.80	465	9,012	142,095	7,010	4,640
LGBM	0.85	239	4,270	142,095	7,010	4,640
LGBM	0.90	390	3,239	142,095	7,010	4,640
RF	0.80	109	91,072	680,402	33,926	22,584
RF	0.85	45	55,466	680,402	33,926	22,584
RF	0.90	203	36,958	680,402	33,926	22,584
XGB	0.80	549	19,079	134,670	6,639	4,393
XGB	0.85	369	11,971	134,670	6,639	4,393
XGB	0.90	489	8,109	134,670	6,639	4,393

We can see that the certainty curve approach underestimates the sample size for low *λ* and overestimates for larger values of *λ*. The other methods consistently overestimate the sample size, often by a very wide margin. It can also be observed that the most accurate sample size estimation is obtained when the *λ* value is 0.85, and this is consistent across all settings.

## Discussion

### Summary

Having a sample size calculator for training ML models allows an analyst to deliberately balance the level of prognostic accuracy against the required sample size. To achieve prognostic accuracy that is closer to the optimal value (obtained with a very large dataset), the larger the sample size needs to be. However, there are no validated sample size calculators that can be used by data analysts, in general and specifically for health datasets, when training ML models. In practice, most clinical predictive ML modeling studies do not provide an explanation for how the sample size was calculated.^3^ This means that analysts need to rely on heuristics or sample size estimation methods that were not designed for ML applications. Oftentimes, researchers rely on sample sizes used in precedents, which tend to also be informed by the same heuristics, or use unsuitable calculators.^27^

In this study, we address this problem by developing an empirically based sample size calculator for tabular health data. The methodology we employ introduces the concept of a certainty curve that is parallel to traditional power analysis concepts. Three calculators were developed for ensemble methods, namely random forests and two boosted tree methods (LGBM and XGBoost). The R code for the calculators is available online.^61^

The analyst needs to determine the certainty level that they wish to have (analogous to the power level) to obtain a prognostic accuracy that is a *λ* proportion of the optimal value (analogous to the effect size). The analyst also needs to provide the various parameters that characterize the dataset, which capture the data complexity. These can be computed using domain knowledge, a small data sample, and/or data from previous studies with similar datasets.

For estimating the sample size, we trained an LGBM model using characteristics of the population level (*entr*, number of *df*, and class imbalance) as predictors. An alternative approach that seems attractive would be to instead use as predictors these characteristics but for the samples (which would be different from the parameter values at the population level). This approach would seemingly increase the accuracy of the calculator. However, such a model will not be of practical use for the estimation of the required sample size for achieving a specific performance target with the required certainty level. This is because in that case, the required sample size would be given by min{*n*: *C*_*λ,m*_(*IF*(*n*), *entr*(*n*), *df*(*n*), *n*) ≥ *C*∗}, and the user would need to provide as input to the sample size calculator the hypothetical values for *IF*, *entr*, and *df* parameters that correspond to a nominal range of possible samples sizes, i.e., as functions of *n* (*IF*(*n*), *entr*(*n*), *df*(*n*)). These functions are largely heterogeneous across different datasets and therefore difficult to determine *a priori* by the user. For all of these reasons, we refrained from using the sample-specific values of the parameters as predictors in the LGBM model for the prediction of the certainty curve, using instead the population-level values, which would be more stable.

Other measures of dataset complexity have been proposed in the literature.^62^ However, these would be difficult to compute without access to complete datasets, which is not going to be the case when estimating sample size during the study design phase of a project.

The results show that our calculator has an accuracy that is significantly better than existing heuristic methods or calculators designed for statistical methods. In fact, our results provide a compelling case against using the heuristics and the sample size calculators designed for regression models, as these tend to significantly overestimate the required sample size.

As shown in Figures S1 and S2, our sample size calculator has the largest error on the BORN and Medical Information Mart for Intensive Care (MIMIC) datasets. It is likely that there are other data characteristics that are not captured by our three model parameters and that this results in the full complexity of these datasets not being captured.

The most accurate results using our sample size calculator are obtained at a *λ* of 0.85, which means that the model performance is at 85% of the optimal model performance with the full population. One explanation for that is that the slope of the relationship between the sample size and ROC-AUC is steepest at that *λ* value across all datasets, making it easier to get more precise sample size estimates. For the 0.8 and 0.9 *λ* values, one can adjust the estimated sample sizes by considering the mRE. For example, if at a *λ* of 0.9, the estimated sample size is 1,000 observations, then it can be adjusted down by 56%–641% for training an LGBM model.

The alternative and currently more often used approach to calculating the sample size for ML models is the Riley et al. method, originally developed for regression models. To illustrate the differences in error, we present some examples of sample size calculations using the certainty curve method, as well as the Riley et al. regression method. For our examples, we assume an LGBM model, *λ* = 0.85, and 80% certainty. For the first example, we use the COVID dataset, which has 47 *df* but a large *IF* of 66.59. The true required sample size is 670, while the one calculated using the certainty curve method is 450. The Riley et al. method, which is highly influenced by the large *IF*, predicts *n* = 19,168, which is an extreme overestimation.

In a different scenario, the WASHINGTON2007 dataset has almost perfect outcome balance (*IF* = 1.04) but a very large number of *df* (11,000). The true sample size is 1,410, and our method predicts *n* = 444—an underestimation. The Riley et al. method predicts *n* = 662,957, which is again an extreme overestimation.

Finally, for the Canadian Community Health Survey (CCHS) dataset, with 11 *df* and *IF* = 1.77, the true required sample size is *n* = 100, and our method predicts *n* = 855, while the Riley et al. method predicts *n* = 3,667.

Therefore, in all examples with different dataset characteristics, the Riley et al. method overestimates the sample size required. In a recent study, this method was assumed to be a lower bound for estimating the sample sizes required for ML prognostic models,^21^ concluding that most clinical prediction studies using ML have sample sizes that are too small. Our results suggest that this regression-based approach for determining sample size is actually overestimating sample size requirements and therefore should not be used to determine minimal sample size requirements.

A recent study examined the impact of sample size on the prognostic performance of multiple types of ML models for a single digital mental health dataset.^5^ Based on that, they recommended a minimal sample size of 750 for simple models and 1,000–1,500 observations for more complex models. These results provide practical guidance within a single domain, and it is uncertain if they can be applied to datasets of varying complexity and in different domains.

Another study developed models to predict sample sizes for untuned ML models and was trained on a mixture of real and synthetic datasets under an assumption of *λ* = 0.97,^63^ with the sample size estimates shown in Table 7. These highlight an important point: setting a high *λ* can result in unrealistic sample sizes for contemporary clinical research. For example, if an estimator recommends 10 million observations for training an ML model with a very high *λ*, then that is not a useful result because it is unlikely that researchers can collect datasets of that size. Furthermore, some of these numbers are larger than the population size. Part of the high sample size estimate values may be attributed to ML models that are not tuned. It is also very likely that the high *λ* values in these estimation models result in a large overestimation of sample size. The *λ* values we used give more realistic sample size estimates and make explicit the performance expectations.

**Table 7:** Sample size estimates for our original 13 datasets using the estimation models in REF.^63^ for random forest and XGBoost models

Dataset	RF	XGB
BORN	89,961	202,410
BSA	6,004	59,595
CALIFORNIA2007	4.8E+13	1,696,640,204
CCHS	6,714,146	1,409,297
COVID	186,712	281,171
FAERS	14,805,033,363	45,100,940
FLORIDA2007	3E+15	10,920,225,797
MIMIC-III	1.59E+52	3.55E+26
Nexoid	N/A^a^	N/A^a^
NEWYORK2007	5.96E+18	3.29E+11
TEXAS	209,326	294,405
WASHINGTON2007	1.47E+25	2.47E+14
WASHINGTON2008	1.50E+22	1.12E+13

The original paper did not have results for LGBM.

a One of the parameters required for using this estimator is the difference in performance between logistic regression and the ML model, and that is used as a measure of non-linearity. The estimator expects the ML model to always perform better. For the Nexoid dataset, logistic regression performed better than XGB, and therefore, this resulted in model misbehavior/failure.

It is clear from our results that the certainty curve method is an improvement over existing heuristic and model-based methods, sometimes by multiple orders of magnitude. Nevertheless, the certainty curve method still exhibits wide variation in its accuracy even when the mRE is quite small, and it can benefit from further work to improve the sample size calculation model.

We have provided an R package that can be used to compute sample size requirements for future studies using these three ML models we investigated.^63^ Future work can extend this study to other types of ML models.

These results and accompanying code can support organizations in their medical device submissions to health regulators that include ML models, whereby the regulators stipulate the need for the use of an adequate sample size.^64^ Furthermore, recent reporting guidelines for ML research studies require the provision of sample size justifications.^65^^,^^66^^,^^67^

### Impact of additional data complexity characteristics

Plots of discrepancies between observed and predicted sample sizes for all 13 datasets, shown in Figures S1 and S2, reveal a systematic discrepancy for the MIMIC-III (underprediction) and BORN (overprediction) datasets. This discrepancy appears to be present in most combinations of ML methods and *λ* values used. One possible explanation for this discrepancy is that the data characteristic variables used as predictors for the model to estimate the certainty curve and predict the required sample size are missing important data characteristics that impact the sample size requirements.

While the data characteristics we considered in the sample size calculator reflect those that can be calculated with reasonable certainty before data collection (during study design) or at early stages of data collection, there may be data complexity characteristics that are influential that are not easily computed. We nevertheless explore such complexity metrics to understand why MIMIC-III and BORN behave differently.

We explored the relevance of additional data complexity metrics, namely L2 and mutual information (MI). The L2 metric has been used to represent data complexity,^62^^,^^68^ and it indicates linear separability (returning the misclassification error of a linear support vector machine (SVM) model on the entire dataset), with high values indicating less linear separability. The MI metric is calculated as the average pairwise MI values across all pairs of predictors in the dataset. High MI indicates that the predictors are dependent on each other, and therefore, there is greater redundancy among them.

We calculated values for these complexity metrics for large-sized subsets (*n* = 10,000, 20,000, 30,000, 40,000, and 50,000) from the original data. Given the computationally intensive nature of these metrics, we used linear regression models of the metric values against the sample size and projected the values for datasets of size *n* = 100,000. Patterns observed for this sample size would be relevant and present in the full datasets as well. The projected values are shown in Table 8.

**Table 8:** The projected values for complexity metrics for the 13 datasets

Dataset	MI	L2
BORN	0.002	0.054
BSA	0.241	0.273
CALIFORNIA2007	0.191	0.189
CCHS	0.069	0.364
COVID	0.082	0.101
FAERS	0.708	0.070
FLORIDA2007	0.129	0.182
MIMIC-III	0.028	0.115
Nexoid	0.002	0.196
NEWYORK2007	0.299	0.172
TEXAS	0.05	0.259
WASHINGTON2007	0.376	0.280
WASHINGTON2008	0.182	0.213

For the BORN dataset, we see that it has the lowest value for L2, indicating a very high linear separability, suggesting that a simple prediction model can achieve high prediction accuracy. Such model simplicity would also explain why a smaller sample size would be sufficient for good performance. This is evident in the plots in Figures 2, 3, and 4. For this type of dataset, the sample size estimation model overestimated the sample size needed to reach high accuracy.

We can see in Figures 2, 3, and 4 that for the MIMIC-III dataset, the benefit of larger sample sizes only occurs at relatively large numbers, with a distinct “elbow” at around *N* = 10,000, which is quite different from the other datasets. Some of this behavior may be explained by the relatively low MI value, suggesting limited redundancy among the predictors, as well as middling L2 separability. Therefore, unlike the BORN dataset, the non-redundant predictors have lower predictive power, requiring a more complex prediction model, and thus more data, to improve performance. Because of the relatively favorable values of other variables (*IF* and *df*), the predicted sample size is not very high; however, the observed values are much higher.

Therefore, additional complexity metrics, which would be difficult to collect during the early stages of a study before data collection, can potentially improve the sample size prediction.

### Sensitivity analysis

There are two types of sensitivity analyses that are relevant to the sample size estimation model. The first pertains to sensitivity to a particular dataset that is used in its training. The second is sensitivity to the accuracy of the input values that characterize a dataset.

With respect to sensitivity to the training dataset, Tables S15–S18 present the mALQE and mRE values after the removal of each training dataset. The mRE results show greater variability than the mALQE results, demonstrating higher sensitivity to individual datasets. For the mRE results, datasets such as COVID, FDA Adverse Event Reporting System (FAERS), WASHINGTON2007, and TEXAS had a sizable impact on the results when they were removed, indicating sensitivity to that performance metric. For example, the mRE for 80% certainty and 0.85 *λ* on the LGBM model changed from 16% to 77.56% for these datasets and from 56.2% to 83.42% for the 0.9 *λ* value. The mALQE results were less impacted by the datasets, demonstrating more robustness to the training data used for that metric. Notwithstanding the sensitivity to these specific training datasets, these results would not change the overall findings from our study.

The user of our sample size estimation model would need to provide three inputs: *entr*, number of *df*, and class imbalance. These values would be needed at an early stage of a study to determine how much data to collect.

The number of *df* (the number of categories for categorical variables) would be known *a priori*, as these would be defined as part of the study design (e.g., response categories in a questionnaire or diagnoses in a set of inclusion criteria). Plus, this information would be available from previous ML modeling work that used similar datasets in the domain, as it is almost always reported as part of descriptive statistics, and ML modeling reporting guidelines require that information to be reported (for example, see Klement and El Emam,^65^ El Emam et al.,^66^ and Collins et al.^67^).

Similarly, the class imbalance would be known from previous studies, and an analyst’s domain knowledge would indicate whether an outcome is rare or common. Such imbalance information is required as part of ML modeling reporting guidelines.^65^^,^^67^

However, the *entr* requires an approximate knowledge of the distribution of the variables. Instead, one needs to rely on previous studies to estimate it or data collected in a smaller feasibility study, which may lead to imprecision in its calculation. To examine this, we performed a post hoc sensitivity analysis to determine how much the predicted sample size using our calculator is affected by inaccuracies in the calculation of the *entr*.

We used 17 medium-sized datasets with binary outcomes^69^^,^^70^^,^^71^^,^^72^^,^^73^^,^^74^^,^^75^^,^^76^^,^^77^^,^^78^^,^^79^^,^^80^^,^^81^^,^^82^^,^^83^^,^^84^^,^^85^ (a summary of these datasets is provided in Table S19) and calculated the estimates for *entr*, number of *df*, and *IF* based on the data. Using these values and our sample size calculator, we estimated the required sample size based on *λ* = 0.85 and a certainty of 0.8. Subsequently, we recalculated the predicted sample size but this time used values for standardized mean entropy that deviate from the original value by some amount. We measure the discrepancy in the sample size prediction as the percentage of relative error given by 100∗(*n*ʹ − *n*)/*n*, where *n* and *n*ʹ denote the calculated sample size when the current and deviated entropy value is used, respectively. Subsequently, we calculated an aggregate measure of discrepancy as the proportion of the datasets where the relative error does not exceed a prespecified value (10%, 25%, and 50%).

Table 9 presents the aggregate discrepancy for different values of standardized entropy deviation. For example, for a deviation of −0.25 in the *entr*, 47% of the sample size estimates were within 10% of the value estimates with the correct *entr*. This means that approximately half the time, the sample size is within 10% of the actual estimated value if there was no error in calculating the *entr* value.

**Table 9:** Aggregate discrepancy (as the percentage of relative difference from the originally predicted value) for the calculated sample size, for different levels of deviation of the mean standardized entropy

Discrepancy	Deviation in mean standardized entropy					
	−0.5	−0.25	−0.1	0.1	0.25	0.5
Median	48	2	0	−1	−1	−1
Within 10%	17.6	47	64.7	41.1	41.1	29.4
Within 25%	23.5	52.9	82.3	70.5	76.4	70.5
Within 50%	58.8	82.35	88.2	100	100	100

Detailed results of the discrepancy for each individual dataset are found in Table S20.

The table shows that medium-level deviation results in modest discrepancy for the sample size estimate. When the deviation of *entr* approaches ±0.5, the sample size estimates become less reliable. Therefore, the sample size estimator is robust to small to medium errors in the calculation of this parameter. However, analysts should still refer to prior studies to get the best approximation of the distribution of predictor variables in a dataset.

### Future work

The error we observe in our estimators may be a function of the heterogeneity of the datasets that we used in our study. This was a deliberate decision to ensure that the results would be applicable across a broad set of health domains. However, to the extent that heterogeneity affected model error, future work can follow the provided methodology to develop new sample size estimators that are suitable for more homogeneous datasets. This can be achieved by having more domain-specific datasets (e.g., many public health datasets) and, for each domain, developing a different sample size estimator that is specific. The development of a catalog of such specific sample size estimators would work best if there were sharp definitions of the domains so that an analyst can easily determine which estimator is most applicable.

Analysis of additional data complexity metrics that cannot be computed at the start of a study, before data collection, indicates that they may capture important data characteristics that impact sample size computation. These were not included in our model because they are not useful in a practical setting where decisions need to be made during study design. However, they do suggest further data complexity metrics that future work should attempt to estimate.

The concepts and methods presented in this study can be extended in future work to genetic data and imaging data to enable sample size calculations for these data modalities.

### Limitations

Our sample size calculator was developed using 13 large datasets. These datasets covered different situations, including public health, hospital discharge, ICUs, registry, adverse events, and health surveys. This heterogeneity arguably improves the generalizability of the results. However, this heterogeneity also likely caused the sensitivity of our results to the training datasets used, as assessed using the mRE. A more homogeneous set of data would provide sample size calculators that are tailored to specific domains and that would more completely capture potential non-linearities that appear in practice between the features and outcomes.

The sample characteristics, such as the *entr*, may need to be estimated from similar datasets. However, our sensitivity analysis indicates that the sample size calculator is tolerant of medium levels of imprecision in the calculation of *entr*.

### Ethics

This study was approved by the CHEO Research Institute Research Ethics Board protocol number 24/36X.

## Resource availability

### Lead contact

Requests for further information and resources should be directed to and will be fulfilled by the lead contact, Khaled El Emam (kelemam@ehealthinformation.ca).

### Materials availability

This study did not generate new unique reagents.

### Data and code availability

The following provides information on the availability of each of the datasets.

• BORN^37^: BORN collects Ontario’s prescribed perinatal, newborn, and child registry with the role of facilitating quality care for families across the province. It can be accessed through a data request at https://bornontario.ca/en/data/data.aspx.
• Basic Stand Alone (BSA)^36^: the BSA inpatient claims dataset is about claim-level information, where each record is an inpatient claim incurred by a 5% sample of Medicare beneficiaries. The dataset is publicly available at https://www.cms.gov/data-research/statistics-trends-and-reports/basic-stand-alone-medicare-claims-public-use-files/bsa-inpatient-claims-puf.
• California, Florida, New York, and Washington State Inpatient Databases (SID)^35^ Healthcare Cost and Utilization Project (HCUP) and Agency for Healthcare Research and Quality: these datasets contain the patient’s hospital discharge information for 2007 and 2008 and are available for purchase at https://hcup-us.ahrq.gov/tech_assist/centdist.jsp.
• CCHS^31^: CCHS data are Canadian population-level information concerning health status, health system utilization, and health determinants collected by Statistics Canada through a telephone survey. The availability of CCHS data is restricted and requires an access request at https://www150.statcan.gc.ca/n1/pub/82-620-m/2005001/4144189-eng.htm.
• COVID-19^30^: the COVID-19 dataset collects Canadian health records of COVID-19 gathered by the Public Health Agency of Canada and is available at Esri Canada (https://resources-covid19canada.hub.arcgis.com/).
• FAERS^33^: FAERS is a database comprising information on adverse event and medication error reports submitted to the FDA and can be downloaded at https://open.fda.gov/data/faers/.
• MIMIC-III^38^: MIMIC-III is a large database that contains deidentified health-related data associated with over 40,000 patients who stayed in critical care units of the Beth Israel Deaconess Medical Center between 2001 and 2012. Access to the MIMIC database is granted upon signing a data use agreement with PhysioNet at https://physionet.org/content/mimiciii/1.4/.
• COVID-19 Survival (Nexoid)^32^: the COVID-19 survival dataset comprises web-based survey data collected by a company called Nexoid in the UK. It is publicly available at https://www.covid19survivalcalculator.com/en/download.
• Texas Hospital Discharge^34^: the TEXAS dataset contains the patient’s hospital discharge information from Texas and is available at (may require a fee) https://www.dshs.texas.gov/center-health-statistics/chs-data-sets-reports/texas-health-care-information-collection/health-data-researcher-information/texas-inpatient-public-use.

The code used in this analysis can be accessed as follows.

• The ML modeling was performed using the R sdgm package available from https://osf.io/DCJM6.^56^
• The R code for applying the sample size estimation models on a new dataset is available from https://osf.io/7bs8q/.^61^

## Acknowledgments

The authors thank Fida Dankar for reviewing an earlier version of this manuscript. This research is funded by the Canada Research Chairs Program through the 10.13039/501100000024Canadian Institutes of Health Research, Discovery Grant RGPIN-2022-04811 from the 10.13039/501100000038Natural Sciences and Engineering Research Council of Canada, and the Canadian Children Inflammatory Bowel Disease Network.

## Author contributions

Conceptualization and design, N.M., K.E.E., and D.L.; analysis, simulations, and drafting of the initial version of the article, N.M., K.E.E., and D.L.; reviewing and revising the article, N.M., K.E.E., and D.L.; drafting the manuscript, N.M., K.E.E., and D.L.; review and editing, N.M., K.E.E., D.L., and T.W.

## Declaration of interests

At the time of writing, K.E.E. was the Scholar-in-Residence at the Office of the Information and Privacy Commissioner of Ontario.

## Supplemental information

Document S1. Figures S1 and S2, Tables S1–S20, and supplemental notes

Document S2. Article plus supplemental information
