# Supplementary material for: Sample size calculation for training ensemble machine learning models on health data
Source: Patterns (N Y). 2026 Mar 26;7(6):101498. doi: 10.1016/j.patter.2026.101498 (PMC13280678; doi:10.1016/j.patter.2026.101498)
Supplement: Document S1. Figures S1 and S2, Tables S1–S20, and supplemental notes [file mmc1.pdf]

Patterns, Volume 7

Supplemental information

Sample size calculation for training ensemble machine learning models on health data

Nicholas Mitsakakis, Dan Liu, Thomas Walters, and Khaled El Emam

Supplemental Methods and Notes

Supplemental Figures

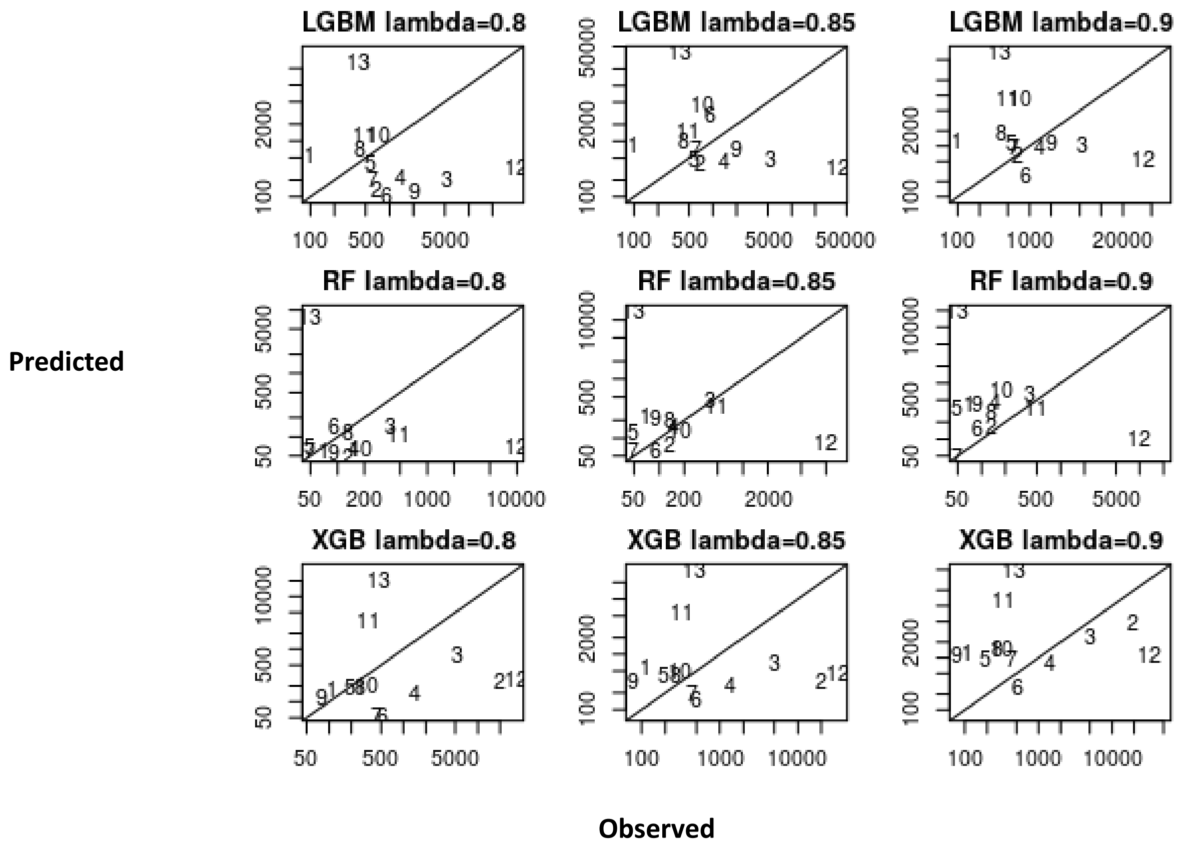

Figure S1: Comparison of the predicted and observed sample sizes for the 13 datasets for different modeling methods and values of lambda, for 80% certainty. Each number in a scatterplot corresponds to a specific dataset, according to the following numbering: 1: cchs, 2: covid, 3: faers, 4: washington2007, 5: texas, 6: nexoid, 7: bsa, 8: california2007, 9: florida2007, 10: newyork2007, 11: washington2008, 12: mimic, 13: born. [S1-S9]

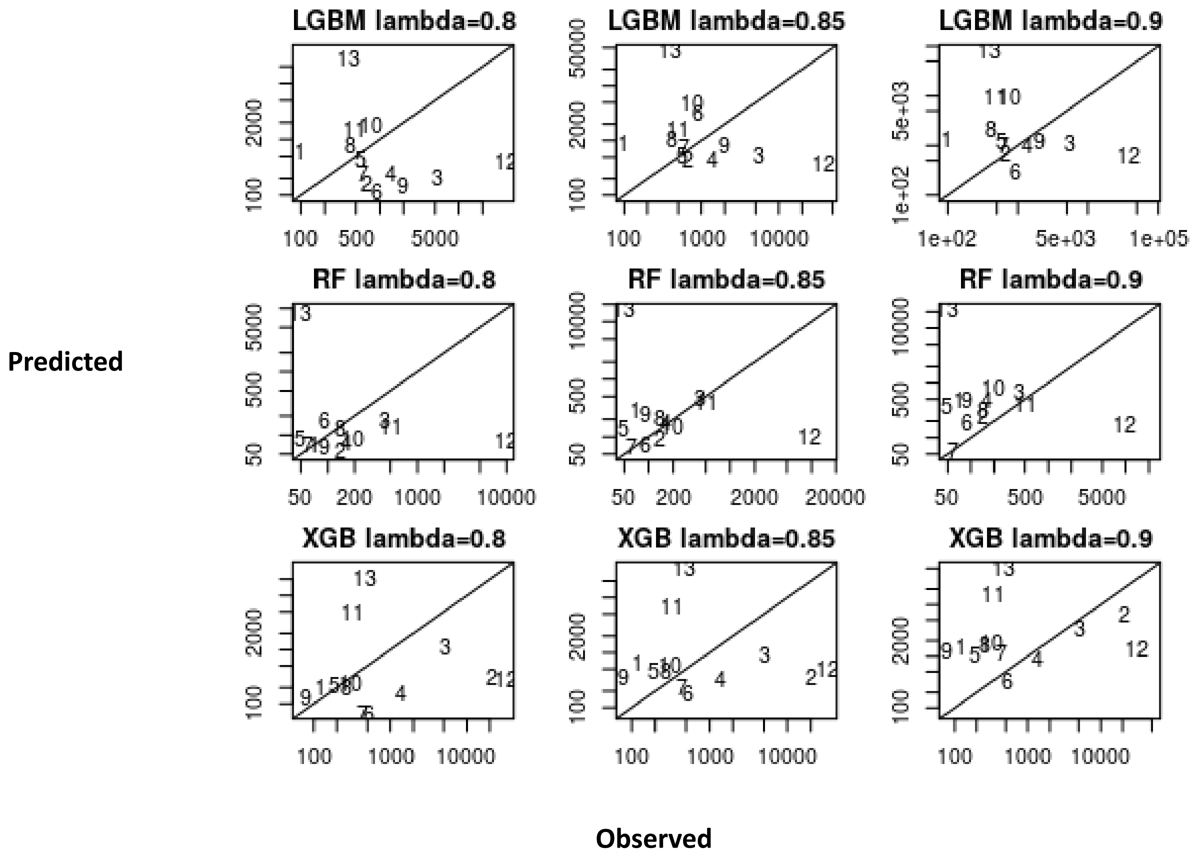

Figure S2: Observed vs predicted required sample sizes for different modeling methods and values of lambda, for 90% certainty.

Supplemental Tables

LGBM				
**Hyperparameter**	**Default**	**Lower bound**	**Upper bound**	**Transform**
booster	1 (gbdt)	1 (gbdt)	2 (goss)	2^learning_rate
max_depth	6	1	15	
learning_rate	log2 (0.3)	-10	0	
early_stopping_rounds	7	7	30	
min_data_in_leaf	10	1	60	
num_leaves	15	4	60	
**Random forest**				
**Hyperparameter**	**Default**	**Lower bound**	**Upper bound**	**Transform**
num.trees	500	1	2000	round(n^min.node.size), where n is the number of observations
min.node.size	0.5	0	1	
max.depth	15	1	50	
min.bucket	10	1	60	
**XGBoost**				
**Hyperparameter**	**Default**	**Lower bound**	**Upper bound**	**Transform**
gamma	0	-15	3	2^gamma
eta	log2(0.3)	-10	0	2^eta
max_depth	6	1	15	
early_stopping_rounds	7	7	30	
max_leaves	15	4	60	
min_child_weight	1	0	7	2^min_child_weight

Table S1: The hyperparameters, their default values, and range for tuning the ensemble models.

Variable	Description	Type	Mean (SD*) or level count (% of total size) or number of categories	Missingness (% of the total size)
Date	The date when a case is reported	Numeric (computed as the number of days since 1st January 2020)	348.07 (96.16)	0.00
Age group	Patient's age group in years	Numeric	<20: 17.92%<br>20-29: 20.26%<br>30-39: 17.08%<br>40-49: 14.82%<br>50-59: 13.53%<br>60-69: 8.26%<br>70-79: 3.99%<br>>80: 2.16%	1.99
Gender	Patient's gender	Categorical	Female: 43.39%<br>Male: 50.05%	0.00
Region	Health unit in Canada	Categorical	40	0.00
Exposure	The type of being exposed to someone with COVID-19	Categorical	Close contact: 31.21%<br>Outbreak: 10.78%<br>Travel-related: 1.14%	0.00
Province	Province in Canada where case is reported	Categorical	Ontario: 70.25%<br>Alberta: 29.75%	0.00
Case status	The status of a patient	Categorical	Recovered: 1.48 %<br>Deceased: 98.52%	0.00

*SD: standard deviation

Table S2: Descriptive statistics for the COVID-19 dataset [S1]

Variable	Description	Type	Mean (SD) or level count (% of total size)	Missingness (% of the total size)
Age	Patient's age in years	Numeric	47.24 (20.19)	0.00
Sex	Patient's gender	Categorical	Female: 45.84%<br>Male: 54.16%	0.00
Education	Patient's highest level of education	Categorical	< Secondary school graduate: 26.14%<br>Secondary school graduate: 16.93%<br>Some post-secondary education: 6.65%<br>Post-secondary certificate: 48.55%	1.72
Marital status	Patient's marital status	Categorical	Married: 43.41%<br>Common-law: 8.12%<br>Widow/separation/divorce: 19.55%<br>Single/never married: 28.74%	0.17
House income	Total household income from all sources	Numeric	57603.42 (32061.49)	9.59
Household size	Size of entire household	Numeric	2.39 (1.23)	15.66
Immigration	Whether a patient is an immigrant	Categorical	Immigrant: 13.50%<br>Non-immigration: 84.11%	1.81
Smoking	Type of smoking	Categorical	Daily smoking: 17.92%<br>Occasional smoking: 2.64%<br>Always occasional smoking: 1.68%<br>Former daily smoking: 25.76%<br>Former occasional smoking: 14.56%<br>Never smoked: 36.91%	0.00
Obesity	Patient's self-reported BMI	Numeric	25.80 (5.15)	0.00
Hypertension	Whether a patient was diagnosed with hypertension	Categorical	Yes: 20.23%<br>No: 79.46%	0.00
Diabetes	Whether a patient was diagnosed with diabetes	Categorical	Yes: 6.99%<br>No: 92.91%	0.00
Physical activity	Daily energy expenditure	Numeric	2.20 (2.43)	0.00
Fruit and vegetable consumption	Daily consumption of fruits and vegetables	Numeric	4.78 (2.53)	0.00
CANHEART	Whether a patient is in ideal cardiovascular health; this is a sum of the prior six	Categorical	Ideal: 63.95%<br>Non-ideal: 36.05%	0.00

	variables.			

Table S3: Descriptive statistics for the CCHS dataset [S2].

Variable	Description	Type	Mean (SD) or level count (% of total size)	Missingness (% of the total size)
Age	Age group in years	Numeric	0_10: 0.58%<br>10_20: 3.35%<br>20_30: 21.56%<br>30_40: 29.84%<br>40_50: 21.09%<br>50_60: 12.48%<br>60_70: 7.47%<br>70_80: 2.97%<br>80_90: 0.54%<br>90_100: 0.12%<br>100_110: 0.01%	0.00
Sex	Patient's gender	Categorical	Female: 63.13%<br>Male: 36.53%	0.34
Race	Patient's race	Categorical	White: 24.19%<br>Hispanic: 1.40%<br>Asian: 1.21%<br>Mixed: 0.96%<br>Black: 0.46%<br>Other: 0.32%	71.45
Smoking	Type of smoking	Categorical	Heavy: 1.68%<br>Medium: 7.64%<br>Light: 4.44%<br>Quit0: 5.54%<br>Quit5: 6.58%<br>Quit10: 9.23%<br>Vape: 5.95%<br>Never smoked: 58.74%	0.19
BMI	Body mass index	Numeric	29.37 (7.81)	0.00
House count	House person count	Numeric	3.14 (1.57)	0.00
Public transport count	Number of public transports used	Numeric	0.38 (1.70)	71.12
Nursing home	Whether it is a nursing home	Categorical	1: 0.07%<br>0: 99.93%	0.00
COVID-19 symptoms	Whether a patient shows symptoms of COVID-19	Categorical	1: 2.04%<br>0: 97.96%	0.00
COVID-19 contact	Whether a patient has close contact with someone infected with COVID-19	Categorical	1: 4.33%<br>0: 95.67%	0.00
Health worker	Whether a patient is a healthcare worker	Categorical	1: 1.79%<br>0: 98.21%	0.00
Asthma	Whether a patient has asthma	Categorical	1: 15.26%<br>0: 84.74%	0.00
Kidney disease	Whether a patient has kidney disease	Categorical	1: 0.36%<br>0: 99.64%	0.00
Liver disease	Whether a patient has liver disease	Categorical	1: 0.21%<br>0: 99.79%	0.00
Heart disease	Whether a patient has heart disease	Categorical	1: 1.87%<br>0: 98.13%	0.00

Lung disease	Whether a patient has lung disease	Categorical	1: 1.45%<br>0: 98.55%	0.00
Diabetes	Whether a patient has diabetes	Categorical	1: 6.17%<br>0: 93.83%	0.00
Hypertension	Whether a patient has hypertension	Categorical	1: 13.83%<br>0: 86.17%	0.00
Outcome	Whether a patient has a high risk of getting infected with COVID-19	Categorical	Yes: 39.20%<br>No: 60.80%	0.00

Table S4: A summary of descriptive statistics for the COVID survival dataset [S3].

Variable	Description	Type	Mean (SD) or level count (% of total size) or number of categories	Missingness (% of the total size)
Outcome	Whether a patient has died	Categorical	Death: 9.94%<br>Non-death: 90.06%	0.00
Event date	Date the adverse event occurred	Numeric<br>(difference from 1/1/2020)	466.52 (827.94)	62.26
Gender	Patient's gender	Categorical	Female: 51.82%<br>Male: 37.85%	10.33
Age	Patient's age in years	Numeric	55.90 (20.80)	33.41
Weight	Patient's weight in kg	Numeric	73.05 (25.70)	74.13
Drug name	Name of medicinal product	Categorical	10,545	0.00
Indication	Medical terminology describing the indication for use	Categorical	4,287	0.00

Table S5: Descriptive statistics for the FAERS dataset [S4].

Variable	Description	Type	Mean (SD) or level count (% of total size) or number of categories	Missingness (% of the total size)
Outcome	Whether a patient's length of stay is greater than 3 days	Categorical	0: 40.42%<br>1: 59.58%	0.00
Age	Patient's age groups	Numeric	0: 11.95%<br>1: 1.66%<br>2: 1.62%<br>3: 1.08%<br>4: 1.39%<br>5: 1.58%<br>6: 1.58%<br>7: 4.84%<br>8: 5.28%<br>9: 4.94%<br>10: 3.75%<br>11: 3.41%<br>12: 3.93%<br>13: 4.96%<br>14: 5.58%<br>15: 5.95%<br>16: 6.47%<br>17: 6.01%<br>18: 5.78%<br>19: 5.32%<br>20: 3.94%<br>21: 2.40%<br>22: 0.21%<br>23: 2.68%<br>24: 2.86%<br>25: 0.54%<br>26: 0.28%	0.00
Sex	Patient's gender	Categorical	Female: 56.25%<br>Male: 37.16%	6.58
Race	Patient's race	Categorical	1 American Indian/Eskimo/Aleut: 0.77%	0.13

			2 Asian or Pacific Islander: 1.68%<br>3 Black: 12.61%<br>4 White: 61.45%<br>5 Other: 23.35%	
Ethnicity	Whether a patient is of Hispanic origin	Categorical	1 Hispanic Origin: 28.14%<br>2 Not of Hispanic Origin: 70.45%	1.41
Location	Patient's mailing address in Texas and contiguous states	Categorical	AR: 0.48%<br>FC: 0.25%<br>LA: 0.21%<br>NM: 0.57%<br>OK: 0.32%<br>TX: 97.21%<br>XX: 0.02%<br>ZZ: 0.92%	0.01
Weekday	The day of week a patient is admitted	Categorical	1 Monday: 16.97%<br>2 Tuesday: 17.22%<br>3 Wednesday: 16.38%<br>4 Thursday: 15.89%<br>5 Friday: 14.98%<br>6 Saturday: 9.42%<br>7 Sunday: 9.14%	0.00
Risk mortality	Risk of mortality score from the All Patient Refined (APR) Diagnosis Related Group (DRG) from the 3M™ APR-DRG Grouper.	Categorical	0 No class specified: 0.10%<br>1 Minor: 60.15%<br>2 Moderate: 20.26%<br>3 Major: 13.26%<br>4 Extreme: 6.22%	0.00
Severity	Severity of illness score from the All Patient Refined (APR) Diagnosis Related Group (DRG) from the 3M™ APR-DRG Grouper.	Categorical	0 No class specified: 0.10%<br>1 Minor: 35.40%<br>2 Moderate: 33.39%<br>3 Major: 22.76%<br>4 Extreme: 8.35%	0.00
DRG	All Patient Refined (APR) Diagnosis Related Group	Categorical	316	0.00

	(DRG) as assigned by 3M APR-DRG Grouper			
Fees	Total non-covered amount of the charge	Numeric	57.51 (1375.47)	0.02

Table S6: Descriptive statistics for the Texas inpatient dataset [S5].

**Variable**	**Description**	**Type**	**Mean (SD) or 1 count (% of total size) or number of categories**	**Missingness (% of the total size)**
Outcome	Whether a patient's length of stay is greater than 3 days	Categorical	Yes: 49.07%<br>No: 50.93%	0.00
Age	Patient's age in years	Numeric	45.58 (28.45)	0.01
Atype	Admission type	Categorical	1: 34.69%<br>2: 18.03%<br>3: 34.23%<br>4: 12.81%<br>5: 0.23%	0.00
Aweekend	Whether admission occurs on a weekend	Categorical	1: 19.32%<br>0: 80.68%	0.00
Died	Whether a patient died during hospitalization	Categorical	1: 1.99%<br>0: 98.01%	0.00
DRG	Diagnosis-related-group (DRG) in effect on discharge date	Categorical	862	0.00
DX1	Primary diagnosis	Categorical	5863	15.14
ZIP	Patient's ZIP code	Categorical	4271	0.06

Table S7: Descriptive statistics for the Washington state hospital discharge dataset [S6].

**Variable**	**Description**	**Type**	**Mean (SD) or 1 count (% of total size) or number of categories**	**Missingness (% of the total size)**
Outcome	Whether the length of stay on a claim is greater than 2.5 days	Categorical	Yes: 57.35%<br>No: 42.65%	0.00
Age	The beneficiary's age	Numeric	1 Under 65: 19.73%<br>2 65- 69: 13.19%<br>3 70-74: 14.65%<br>4 75-79: 15.55%<br>5 80-84: 16.10%<br>6 85 & older: 20.78%	0.00
Gender	The beneficiary's gender	Categorical	1 Male: 56.12%<br>2 Female: 43.88%	0.00

DRG	Diagnostic related groups to which a hospital claim belongs for prospective payment purposes	Categorical	311	0.00
ICD-9	Primary procedure (primarily surgical procedures) performed during the inpatient stay	Categorical	85	47.00
Payment	Quintile value (or code) to which the actual Medicare payment amount on the claim belongs	Categorical	1: 19.97%<br>2: 20.12%<br>3: 20.00%<br>4: 19.82%<br>5: 20.09%	0.00

Table S8: Descriptive statistics for the Basic Stand Alone inpatient claims dataset [S7].

**Variable**	**Description**	**Type**	**Mean (SD) or level count (% of total size) or number of categories**	**Missingness (% of the total size)**
Outcome	Whether a patient's length of stay is greater than 2 days	Categorical	1: 74.58%<br>0: 25.42%	0.00
AGE	Patient's age in years	Numeric	45.79 (28.43)	0.01
FEMALE	Whether a patient's gender is female	Categorical	1 Female: 58.69%<br>0 Male: 41.31%	0.01
RACE	Patient's race	Categorical	1 White: 23.86%<br>2 Black: 1.18%<br>3 Hispanic: 2.80%<br>4 Asian or Pacific Islander: 1.31%<br>5 Native American: 0.48%<br>6 Other: 0.02%	70.34
ATYPE	Admission type	Categorical	1 Emergency: 35.59%<br>2 Urgent: 17.80%<br>3 Elective: 33.22%<br>4 Newborn: 12.74%<br>5 Trauma Center: 0.65%	0.00
AWEEKEND	Whether a patient's admission day is on a weekend	Categorical	1 Admitted Saturday - Sunday: 19.50%<br>0 Admitted Monday - Friday: 80.50%	0.00
DRG	Diagnosis Related Group	Categorical	746	0.00
DX1	ICD-9-CM Diagnosis	Categorical	6149	0.00
PAY1	Expected primary payer (Medicare, Medicaid, private insurances, etc.)	Categorical	1 Medicare: 31.21%<br>2 Medicaid: 20.03%<br>3 Private insurance: 42.89%<br>4 Self-pay: 2.80%<br>5 No charge: 0.60%<br>6 Other: 2.46%	0.00
TOTCHG	Total charges	Numeric	26040.52 (43943.20)	0.01
ZIP	Zip code	Categorical	4192	0.00
CHRON1	ICD-9-CM Chronic Condition Indicators	Categorical	1 Chronic condition: 35.16%<br>0 Non-chronic condition: 64.82%	0.01
CHRONB1	Chronic Condition Indicators - body system	Categorical	18	0.01

PCLASS1	Procedure Classes Refined for ICD-10-PCS procedure codes	Categorical	1 Minor diagnostic: 6.66%<br>2 Minor therapeutic: 24.98%<br>3 Major diagnostic: 0.44%<br>4 Major Therapeutic: 31.07%	36.84
CM_ALCOH	AHRQ comorbidity measure for ICD-9-CM codes: alcohol abuse	Categorical	1 Comorbidity is present: 3.01%<br>0 Comorbidity is not present: 96.99%	0.00
CM_DEPRE	AHRQ comorbidity measure for ICD-9-CM codes: depression	Categorical	1 Comorbidity is present: 6.31%<br>0 Comorbidity is not present: 93.69%	0.00
CM_HTN_C	AHRQ comorbidity measure for ICD-9-CM codes: hypertension (combine uncomplicated and complicated)	Categorical	1 Comorbidity is present: 28.69%<br>0 Comorbidity is not present: 71.31%	0.00
CM_OBESE	AHRQ comorbidity measure for ICD-9-CM codes: obesity	Categorical	1 Comorbidity is present: 5.55%<br>0 Comorbidity is not present: 94.45%	0.00

Table S9: Descriptive statistics for the hospital Washington dataset [S6].

Variable	Description	Type	Mean (SD) or level count (% of total size) or number of categories	Missingness (% of the total size)
Outcome	Whether a patient's length of stay is greater than 3 days	Categorical	1: 54.10%<br>0: 45.90%	0.00
AGE	Patient's age in years	Numeric	44.59 (28.58)	0.95
FEMALE	Whether a patient's gender is female	Categorical	1 Female: 57.24%<br>0 Male: 39.85%	2.91
RACE	Patient's race	Categorical	1 White: 46.72%<br>2 Black: 7.27%<br>3 Hispanic: 28.52%<br>4 Asian or Pacific Islander: 7.07%<br>5 Native American: 0.07%<br>6 Other: 2.04%	8.31
AWEEKEND	Whether a patient's admission day is on a weekend	Categorical	1 Admitted Saturday - Sunday: 20.39%<br>0 Admitted Monday - Friday: 79.61%	0.00
DRG	Diagnosis Related Group	Categorical	746	0.00
DX1	ICD-9-CM Diagnosis	Categorical	8548	0.00
PAY1	Expected primary payer (Medicare, Medicaid, private insurances, etc.)	Categorical	1 Medicare: 31.13%<br>2 Medicaid: 25.57%<br>3 Private insurance: 34.79%<br>4 Self-pay: 3.41%<br>6 Other: 5.09%	0.02
TOTCHG	Total charges	Numeric	45065.28 (78294.38)	12.00
CHRON1	ICD-9-CM Chronic Condition Indicators	Categorical	1 Chronic condition: 34.22%<br>0 Non-chronic condition: 65.78%	0.00
CHRONB1	Chronic Condition Indicators - body system	Categorical	18	0.00
PCLASS1	Procedure Classes Refined for ICD-10-PCS procedure codes	Categorical	1 Minor diagnostic: 9.65%<br>2 Minor therapeutic: 28.12%<br>3 Major diagnostic: 0.46%<br>4 Major Therapeutic: 26.37%	35.40
CM_ALCOH	AHRQ comorbidity measure for ICD-9-CM codes: alcohol abuse	Categorical	1 Comorbidity is present: 3.84%<br>0 Comorbidity is not present: 96.16%	0.00

CM_DEPRE	AHRQ comorbidity measure for ICD-9-CM codes: depression	Categorical	1 Comorbidity is present: 5.80%<br>0 Comorbidity is not present: 94.20%	0.00
CM_HTN_C	AHRQ comorbidity measure for ICD-9-CM codes: hypertension (combine uncomplicated and complicated)	Categorical	1 Comorbidity is present: 33.31%<br>0 Comorbidity is not present: 66.69%	0.00
CM_OBESE	AHRQ comorbidity measure for ICD-9-CM codes: obesity	Categorical	1 Comorbidity is present: 7.23%<br>0 Comorbidity is not present: 92.77%	0.00

Table S10: Descriptive statistics for the hospital California dataset [S6].

Variable	Description	Type	Mean (SD) or level count (% of total size) or number of categories	Missingness (% of the total size)
Outcome	Whether a patient's length of stay is greater than 3 days	Categorical	1: 60.46%<br>0: 39.54%	0.00
AGE	Patient's age in years	Numeric	51.23 (27.04)	0.00
FEMALE	Whether a patient's gender is female	Categorical	1 Female: 56.02%<br>0 Male: 43.98%	0.00
RACE	Patient's race	Categorical	1 White: 65.53%<br>2 Black: 16.92%<br>3 Hispanic: 13.28%<br>4 Asian or Pacific Islander: 0.76%<br>5 Native American: 0.27%<br>6 Other: 2.47%	0.76
ATYPE	Admission type	Categorical	1 Emergency: 54.15%<br>2 Urgent: 16.43%<br>3 Elective: 20.73%<br>4 Newborn: 8.17%<br>5 Trauma Center: 0.52%	0.00
AWEEKEND	Whether a patient's admission day is on a weekend	Categorical	1 Admitted Saturday - Sunday: 19.51%<br>0 Admitted Monday - Friday: 80.49%	0.00
DRG	Diagnosis Related Group	Categorical	861	0.00
DX1	ICD-9-CM Diagnosis	Categorical	7380	0.00
PAY1	Expected primary payer (Medicare, Medicaid, private insurances, etc.)	Categorical	1 Medicare: 42.71%<br>2 Medicaid: 17.50%<br>3 Private insurance: 27.52%<br>4 Self-pay: 6.23%<br>5 No charge: 2.28%<br>6 Other: 3.77%	0.00
TOTCHG	Total charges	Numeric	33604.48 (52812.95)	0.01
ZIP	Zip code	Categorical	14729	0.00

Note: SD: standard deviation

Table S11: Descriptive statistics for the hospital Florida dataset [S6].

Variable	Description	Type	Mean (SD) or level count (% of total size) or number of categories	Missingness (% of the total size)
Outcome	Whether a patient's length of stay is greater than 3 days	Categorical	1: 61.82%<br>0: 38.18%	0.00
AGE	Patient's age in years	Numeric	48.87 (27.36)	0.00
FEMALE	Whether a patient's gender is female	Categorical	1 Female: 56.68%<br>0 Male: 43.32%	0.00
RACE	Patient's race	Categorical	1 White: 56.73%<br>2 Black: 17.43%<br>3 Hispanic: 13.56%<br>4 Asian or Pacific Islander: 3.38%<br>5 Native American: 1.01%<br>6 Other: 5.90%	1.98
ATYPE	Admission type	Categorical	1 Emergency: 60.22%<br>2 Urgent: 9.86%<br>3 Elective: 20.86%<br>4 Newborn: 8.90%<br>5 Trauma Center: 0.00%	0.16
AWEEKEND	Whether a patient's admission day is on a weekend	Categorical	1 Admitted Saturday - Sunday: 19.26%<br>0 Admitted Monday - Friday: 80.74%	0.00
DRG	Diagnosis Related Group	Categorical	863	0.00
DX1	ICD-9-CM Diagnosis	Categorical	7956	0.00
PAY1	Expected primary payer (Medicare, Medicaid, private insurances, etc.)	Categorical	1 Medicare: 36.08%<br>2 Medicaid: 23.69%<br>3 Private insurance: 32.38%<br>4 Self-pay: 5.44%<br>5 No charge: 0.17%<br>6 Other: 2.23%	0.00
TOTCHG	Total charges	Numeric	24628.84 (43545.43)	0.01
ZIP	Zip code	Categorical	10814	0.00
CHRON1	ICD-9-CM Chronic Condition Indicators	Categorical	1 Chronic condition: 40.86%<br>0 Non-chronic condition: 59.14%	0.00
CHRONB1	Chronic Condition Indicators - body system	Categorical	18	0.00

PCLASS1	Procedure Classes Refined for ICD-10-PCS procedure codes	Categorical	1 Minor diagnostic: 15.19%<br>2 Minor therapeutic: 31.30%<br>3 Major diagnostic: 0.54%<br>4 Major Therapeutic: 25.86%	27.12

Table S12: Descriptive statistics for the hospital New York dataset [S6].

Variable	Description	Type	Mean (SD) or level count (% of total size) or number of categories	Missingness (% of the total size)
Birth weight	Whether a newborn baby has low birthweight (<2,500 grams)	Categorical	1: 7.01%<br>0: 92.99%	0.00
Gestational age	Gestational age of a newborn baby	Numeric	1 < 34 weeks: 2.32%<br>2 34-36 weeks: 6.00%<br>3 37-38 weeks: 27.15%<br>4 39-41 weeks: 64.11%<br>5 >=42 weeks: 0.43%	0.00
Maternal age	Maternal age in years at time of stillbirth or live birth	Numeric	1 <= 19: 2.15%<br>2 20-34: 74.12%<br>3 35-39: 19.31%<br>4 >=40: 4.36%	0.06
Maternal BMI	Maternal pre-pregnancy body mass index	Numeric	1 <18.5: 4.53%<br>2 18.5-24.9: 43.44%<br>3 25-29.9: 20.22%<br>4 >=30: 15.86%	15.94
Parity	Total number of pregnancies a mother has experienced	Numeric	0: 42.82%<br>1: 34.57%<br>2: 13.98%<br>3: 4.64%<br>>=4: 2.89%	1.10
Preterm birth	Number of previous preterm pregnancies	Numeric	0: 93.57%<br>1: 4.41%<br>2: 0.71%<br>3: 0.13%<br>>=4: 0.05%	1.13
Abortions	Number of previous abortions	Numeric	0: 65.77%<br>1: 20.85%<br>2: 7.25%<br>3: 2.50%<br>>=4: 1.50%	2.13
Smoking	Maternal smoking status at time of admission	Categorical	Yes: 7.64%<br>No: 88.14%	4.21
Alcohol	Alcohol exposure in pregnancy	Categorical	Yes: 2.23%<br>No: 92.54%	5.23
Prenatal screening	Whether a mother has prenatal screening during pregnancy	Categorical	Yes: 66.30%<br>No: 33.70%	0.00

Addiction	Mental health concern regarding addiction	Categorical	Yes: 0.60%<br>No: 93.72%	5.68
Anxiety	Mental health concern regarding anxiety	Categorical	Yes: 8.99%<br>No: 85.33%	5.68
Depression	Mental health concern regarding depression	Categorical	Yes: 7.58%<br>No: 86.74%	5.68
Diabetes	Maternal health condition regarding diabetes	Categorical	Yes: 1.00%<br>No: 93.27%	5.73
Genetics	Maternal health condition regarding genetics	Categorical	Yes: 0.00%<br>No: 94.26%	5.73
Cocaine drug	Drug exposure to Cocaine in pregnancy	Categorical	Yes: 0.25%<br>No: 94.65%	5.10
Hallucinogens drug	Drug exposure to Hallucinogens in pregnancy	Categorical	Yes: 0.02%<br>No: 94.88%	5.10
Opioids drug	Drug exposure to Opioids in pregnancy	Categorical	Yes: 0.42%<br>No: 94.48%	5.10

Table S13: Descriptive statistics for the BORN dataset [S8].

Variable	Description	Type	Mean (SD) or level count (% of total size) or number of categories	Missingness (% of the total size)
Readmission	Whether a patient is re-admitted to ICU	Categorical	1 Yes: 21.01%<br>0 No: 78.99%	0.00
Age	Patient's age in the time of first admission	Numeric	63.43 (16.16)	0.00
Ethnicity	Patient's ethnicity group	Categorical	38	15.14%
Admission type	Patient's admission type	Categorical	Elective: 18.10%<br>Emergency: 78.52%<br>Urgent: 3.37%	0.00
Heart rate	Vital sign for heart rate	Numeric	87.86 (15.89)	0.47%
NT-proBNP	Lab test for N-terminal prohormone of brain natriuretic peptide	Numeric	4.10 (1.17)	43.55%
Creatinine	Lab test for serum creatinine	Numeric	4.10 (1.17)	43.54%
Bun	Lab test for blood urea nitrogen	Numeric	4.10 (1.17)	43.53%
Potassium	Lab test for potassium	Numeric	4.10 (1.17)	43.52%
Cholesterol	Lab test for cholesterol	Numeric	4.10 (1.17)	43.54%

Table S14: Descriptive statistics for the MIMIC-III dataset [S9].

	**LGBM**			**RF**			**XGB**		
**lambda**	0.80	0.85	0.90	0.80	0.85	0.90	0.80	0.85	0.90
cchs	-73.45	1.88	26.22	-46.09	8.44	104.81	-58.71	0.25	260.81
covid	-47.52	77.56	83.42	-23.82	14.51	166.26	-30.13	59.16	395.98
faers	-47.52	77.56	83.42	-23.82	14.51	166.26	-30.13	59.16	395.98
washington2007	-47.52	77.56	83.42	-23.82	14.18	104.81	-30.13	59.16	395.98
texas	-73.45	77.56	26.22	-46.09	8.44	104.81	-58.71	0.25	260.81
nexoid	-47.52	1.88	83.42	-46.09	14.51	166.26	-30.13	59.16	395.98
bsa	-54.18	62.98	53.40	-46.09	8.44	166.26	-30.13	59.16	395.98
california2007	-73.45	1.88	26.22	-46.09	8.44	140.23	-58.71	0.25	260.81
florida2007	-47.52	77.56	83.42	-32.55	8.44	104.81	-58.71	0.25	260.81
newyork2007	-73.45	1.88	26.22	-23.82	14.51	104.81	-55.87	2.68	261.76
washington2008	-73.45	1.88	26.22	-23.82	14.51	166.26	-58.71	0.25	260.81
mimic	-47.52	77.56	83.42	-23.82	14.51	166.26	-30.13	59.16	395.98
born	-73.45	1.88	26.22	-46.09	8.44	104.81	-58.71	0.25	260.81
**default**	**-66.8**	**16.5**	**56.2**	**-37.4**	**8.8**	**130.8**	**-33**	**56.7**	**395**

Table S15: The percentage mRE sensitivity to the dataset at 80% certainty.

	LGBM			RF			XGB		
lambda	0.80	0.85	0.90	0.80	0.85	0.90	0.80	0.85	0.90
cchs	-68.58	8.31	36.11	-40.16	8.58	127.17	-50.07	13.99	313.33
covid	-41.28	92.19	94.50	-17.87	11.13	170.17	-22.35	81.06	479.88
faers	-41.28	92.19	94.50	-17.87	11.13	170.17	-22.35	81.06	479.88
washington2007	-41.28	92.19	94.50	-17.87	10.41	127.17	-22.35	81.06	479.88
texas	-68.58	92.19	36.11	-40.16	8.58	127.17	-50.07	13.99	321.97
nexoid	-41.28	8.31	94.50	-40.16	11.13	170.17	-22.35	81.06	479.88
bsa	-48.55	75.18	65.85	-40.16	8.58	170.17	-22.35	81.06	479.88
california2007	-68.58	8.31	36.11	-40.16	8.58	159.94	-50.07	20.44	313.33
florida2007	-41.28	92.19	94.50	-29.95	8.58	127.17	-50.07	13.99	313.33
newyork2007	-68.58	8.31	36.11	-17.87	11.13	127.17	-49.70	13.99	313.33
washington2008	-68.58	8.31	36.11	-17.87	11.13	170.17	-50.07	13.99	313.33
mimic	-41.28	92.19	94.50	-17.87	11.13	170.17	-22.35	81.06	479.88
born	-68.58	8.31	36.11	-40.16	8.58	127.17	-50.07	13.99	313.33
default	-61.3	25.3	64.8	-28.1	9.3	137.4	-22.7	74.6	471.2

Table S16: The percentage mRE sensitivity to the dataset at 90% certainty.

	LGBM			RF			XGB		
**lambda**	0.80	0.85	0.90	0.80	0.85	0.90	0.80	0.85	0.90
cchs	449.51	214.95	287.82	139.87	51.15	163.90	740.41	362.28	395.97
covid	491.24	222.31	381.43	89.14	51.15	260.03	608.45	362.28	419.38
faers	441.04	214.95	287.82	89.14	57.65	260.03	740.41	362.28	419.38
washington2007	441.04	222.31	381.43	105.01	57.65	214.94	608.45	364.81	419.38
texas	491.24	222.31	381.43	139.87	51.15	163.90	740.41	420.48	418.39
nexoid	441.04	215.36	381.43	139.87	57.65	260.03	608.45	420.48	419.38
bsa	491.24	222.31	381.43	139.87	57.65	260.03	662.72	420.48	419.38
california2007	491.24	222.31	319.76	139.87	53.28	260.03	740.41	420.48	395.97
florida2007	441.04	222.31	381.43	139.87	51.15	163.90	740.41	420.48	395.97
newyork2007	491.24	214.95	287.82	89.14	57.65	163.90	740.41	420.48	419.38
washington2008	491.24	214.95	287.82	89.14	57.65	260.03	608.45	362.28	395.97
mimic	441.04	214.95	287.82	89.14	51.15	163.90	608.45	362.28	395.97
born	441.04	214.95	287.82	89.14	51.15	163.90	608.45	362.28	395.97
**default**	**482**	**222**	**345**	**121**	**55**	**202**	**681**	**418**	**397**

Table S17: The percentage mALQE sensitivity to the dataset at 80% certainty.

	LGBM			RF			XGB		
**lambda**	0.80	0.85	0.90	0.80	0.85	0.90	0.80	0.85	0.90
cchs	383.45	219.29	276.08	113.28	35.77	168.17	602.46	356.05	479.81
covid	480.32	246.61	408.59	97.90	39.42	266.97	436.15	356.05	495.36
faers	383.45	219.29	290.14	97.90	49.28	266.97	602.46	356.05	495.36
washington2007	396.97	246.61	408.59	101.56	49.28	224.85	436.15	396.00	495.36
texas	480.32	246.61	408.59	113.28	35.77	168.17	602.46	396.00	495.36
nexoid	383.45	219.29	408.59	113.28	49.28	266.97	436.15	396.00	495.36
bsa	480.32	246.61	408.59	113.28	49.28	266.97	480.18	396.00	495.36
california2007	480.32	246.61	276.08	113.28	35.77	266.97	602.46	396.00	479.81
florida2007	383.45	246.61	408.59	113.28	35.77	168.17	602.46	382.74	479.81
newyork2007	480.32	219.29	276.08	97.90	49.28	168.17	602.46	396.00	486.54
washington2008	480.32	226.63	276.08	97.90	49.28	266.97	436.15	356.05	479.81
mimic	383.45	219.29	276.08	97.90	35.77	168.17	436.15	356.05	479.81
born	383.45	219.29	276.08	97.90	35.77	168.17	436.15	356.05	479.81
**default**	**465**	**239**	**390**	**109**	**45**	**203**	**549**	**369**	**489**

Table S18: The percentage mALQE sensitivity to the dataset at 90% certainty.

Dataset #	Data Name	Description	Number of Records	Number of Variables
1	Adult	The census income data from 1994 Census database	44842	13
2	BankNote	Data of images that were taken for the evaluation of tan authentication procedure for banknotes.	1371	5
4	Breast Cancer Wisconsin	Diagnostic Wisconsin Breast Cancer Database	683	9
4	Breast Cancer Coimbra	Diagnostic Coimbra Breast Cancer Database	116	9
5	Breast Cancer	This data is provided by the Oncology Institute to predict the breast cancer.	227	10
6	Chronic Kidney Disease	This dataset is collected in Apollo Hospital, India. It can be used to predict the chronic kidney disease.	209	21
7	Heart Disease	The Cleveland heart database	303	13
8	Colposcopy/green	The three modalities of data are dedicated to determining two classes of the colposcopic sequences (bad, good).	98	56
9	Colposcopy/hinselmann		97	56
10	Colposcopy/schiller		92	56
11	Diabetic Mellitus	The data is dedicated to determining the type of diabetic mellitus. This dataset is from OpenML.	281	97
12	Diabetic Retinopathy	This dataset contains features extracted from the Messidor image set to predict whether an image contains signs of diabetic retinopathy or not.	1151	19
13	EEGb Eye State	All data is from one continuous EEG measurement with the Emotiv EEG Neuroheadset. The data set consists of 14 EEG values and a value indicating the eye state.	14980	14
14	Stroke	Health care database to predict stroke	29072	10
15	Thoracic Surgery	The data is dedicated to the classification problem related to the post-operative life expectancy in lung cancer patients.	470	16
16	Titanic_train	The train dataset on Kaggle is a subset of the passenger information on Titanic. This dataset is used to predict whether the passenger survived or not. This dataset is from Kaggle.	891	8
17	Z-Alizadeh Sani	This data is used to predict two possible categories of CAD (normal or not normal).	303	56

Table S19: Datasets [S10-S26] used for performing the sensitivity analysis of mean standardized entropy. Unless otherwise stated, the datasets are available from the UCI repository.

Dataset #	Deviation in Standardized Mean Entropy					
	**-0.5**	**-0.25**	**-0.1**	**0.1**	**0.25**	**0.5**
1	0.9	0.0	0.0	-41.7	-27.8	-34.2
2	40.5	40.5	-17.7	-2.0	-11.3	-11.3
3	38.8	38.8	1.0	-9.9	-8.7	-8.7
4	95.9	94.2	94.2	40.1	26.0	27.7
5	1736.1	0.0	0.0	0.0	-25.0	-11.8
6	0.0	0.0	0.0	0.0	0.0	0.0
7	46.3	17.6	8.8	82.1	82.1	82.1
8	-33.5	-29.2	-22.7	-0.1	-17.8	-17.8
9	5141.9	-0.8	0.0	0.0	0.0	-20.1
10	3126.6	0.0	0.0	-19.5	-12.0	-26.7
11	3977.4	0.0	0.0	-43.6	-27.8	-35.1
12	95.9	94.2	94.2	40.1	26.0	27.7
13	26.4	26.9	26.9	-9.2	8.5	63.6
14	16.4	16.4	1.6	-12.1	-13.9	-13.9
15	-9.0	0.0	0.0	-41.3	-16.9	-24.1
16	22.6	22.6	0.6	-7.2	-8.8	-8.8
17	38.6	38.6	-21.9	-0.2	-8.9	-8.9

Table S20: The discrepancy results for the required sample size estimation corresponding to deviation from mean entropy value, as input to the sample size calculator. Discrepancy is measured by the percent relative error given by 100*(n’-n)/n, where n, n’ denotes the calculated sample size when the measured and deviated entropy value is used, respectively.

Supplemental Notes

Previous Sample Size Estimation Studies

Figueroa et at [S27], discusses a learning curve fitting approach, proposed also by Mukherjee et al [S28]. The authors used an inverse power law method to estimate the effect of training data size and accuracy of a classifier. The method requires the estimate of a performance measure for training data of different sizes. The authors use 3 large datasets to validate their method. It can be used in real applications in order to determine if additional data would be beneficial in terms of significantly increasing the performance of the classifier. However, it cannot be used for determining a-priori and without training a model on existing data what the required sample size of the training data would be.

Raudys and Jain [S29] reviewed a number of statistical pattern recognition methods for binary classification, focusing on the effect of training and testing sample size on feature selection and error estimation. The investigation includes mainly parametric methods (FDA, QDA, Parzen Window classifier) as well as KNN. They summarize the number of observations in the smallest class needed so that the classification error estimated by the sample is less than 50% larger than the theoretical classification error (produced by an infinite size sample). This investigation relies on normality and other assumptions. It includes a series of theoretical results that can be used for specific cases and under specific assumptions. As such, generalization of these results requires a lot of caution.

Fukunara and Hayes [S30] investigate the effect of sample size on the performance of a binary classifier. They consider the cases of Linear and Quadratic classifiers under the assumption of normally distributed data. They provide mathematical expressions of how sample size of the training and test set affect the estimation of important parameters of the underlying distribution (such as means and covariate matrix), which are needed for the classifiers under investigation.

Larracy and colleagues [S31] used a simulation to investigate the effect of training sample size, validation framework and feature selection method, under different scenarios of discriminability in binary classification problems. They also assess the performance of learning curves as tools for forecasting the improvement of ML models with larger training data sizes. The study confirms that nested CV is the preferred validation method when the sample size is small. However, it has various limitations: the data simulation mechanism is simplistic (all features are normally distributed with equal standard deviations, while their mean determines the two classes, and the discriminability between them). The only ML method that is used is linear SVM. Sample sizes under consideration range from 10 to 600, where the performance of the models seems to plateau.

van der Ploeg et al [S32] use simulated population data generated based on three existing clinical datasets and investigated the performance of four ML models, as well as logistic regression, when trained and tested on subsets of the population sets with varying sizes. They reported “optimism” of each model (i.e., how well the model performed in the training data, with comparison to the test data), concluding that ML modeling methods are “data hungry” when compared to traditional logistic regression. The study has some limitations including ignoring the essential step of tuning for the ML models, using a limited set of simulated population dataset scenarios, and using optimism as a measure of model’s performance is of limited utility in practice, as the “test” performance can be directly evaluated using resampling methods (e.g. cross-validation).

Vabalas and colleagues [S33] investigate how the settings around training and model assessment affect the performance of the model and the accuracy of the assessment. They explore the literature on the topic focusing on the application of ML methods in studies on autism, using meta-analysis on the effect of sample size and model accuracy. Furthermore, they perform simulations, on varying sample sizes (n=200 to 1000), two-class data with 50 normally distributed features (similar to Laracy et al.), distinguishing two scenarios of discriminability (all features being noise vs 10 discriminating features and 40 noise) and 3 approaches for feature selection. They use SVM as their ML model, and distinguish between a number of validation strategies (split, k-fold CV, nested CV). They show that for small sample sizes, nested CV gives a more accurate assessment of model performance than simple CV (where feature selection and tuning

happens a priori). In their simulations, the models' performance seems to plateau at a sample size of around 600.

McNemara and colleagues [S34] also study the effect of sample size on the performance of ML models, using an extensive simulation. They are particularly interested in the robustness of the models under training against measurement error in the predictors, in addition to mislabeling error, and presence of features that are unrelated with the outcome. They compare the performance of gradient boosted machines, random forests, logistic regression with regularization (elastic net), using the Area Under the Curve and binary log loss as performance measures. They found that the tree-based models achieve superior performance that plateaus at around n=3000. Overall, the study is well designed and provides useful insights, however the simulation data mechanism is limited to a couple of specific approaches and results cannot be generalized to an arbitrary setting of real data.

Zantvoort et al. [S35] evaluated prognostic improvement as data size increases for a single mental health dataset, also examining the impact of the number of features and the complexity of the features. They provide guidance on appropriate minimal sample sizes in this domain.

In addition, the following studies use specific data types different from clinical data.

Acharjee and colleagues [S36] use a simulation to validate a number of variable selection methods coupled with random forests. Their method aims to guide study design and required size of training data, but mainly focus on "omics" applications (genomics, metabolomics) and it is uncertain if these approaches can be extended to different fields.

D'souza and colleagues [S37] investigate the effect of the "structural optimization" of CNN models on the accuracy, for classification problems, under different sample sizes. Various candidate structures were generated over the choices of the number of layers and weights with a constrained value of the Vapkin-Chervonenkis (VC) dimension (a measure of the complexity of the model, calculated by an expression provided in Bartlet et al [S38]). Their analysis uses samples of varying sizes from three large image datasets (MNIST, CIFAR10, mitosis). Along other details, they conclude that the influence of the network structure is larger when the training sample size is small. Their study can provide various insights on the importance of finding an optimal network structure, under different sample size constraints. However, the applicability of these findings to other types of ML models is very limited, considering also the very limited number of datasets used (only 3) and variety of training data sizes (100, 500, 1000).

In the work of Beleitas et al [S39] the effect of the size of both the training and the test sample on the accuracy and precision of the performance measure is investigated. The authors use real and simulated sprectroscopy data and they apply an LDA model using 10 latent variables (derived by the application of a partial least square model) as features. They focus exclusively on multiclass sensitivity as performance measure, and they assess how well a learning curve is able to forecast the performance of a model with a larger training dataset. They provide a simple calculation of required sample size for achieving desired precision (as measured by the width of the confidence interval of the sensitivity). They primarily focus on very small sample sizes (n=20), which are more relevant in the field of mass spectrometry analysis. Their analysis provides some interesting insights, but it has limited generalizability to the general context of sample size requirements in ML applications.

Balki et al. [S40] investigate the issue of sample size requirements and its effect on model performance in the field of medical imaging. They conduct a systematic review and find a limited number of studies (n=18) that assess the performance of their models with respect to the size of training data. An even smaller number of studies (n=4) attempt to develop a methodology for specifying the required sample size for obtaining the desired level of accuracy. These methodological approaches can be divided into pre-hoc (model based) and post-hoc (curve-fitting). The former use theoretical findings for specific models (e.g. 1-hidden layer feed-forward neural network) and construct mathematical rules and expressions for guiding the determination of required sample size. The latter are used for fitting a curve of model's accuracy on training data size, which can then be used for forecasting the required size for obtaining a desired performance level. This type of methodology is relevant and useful in the setting of medical imaging where the size of training data depends on the capacity to label the images. The required sample size is not known a-priori, but it can be determined after training and assessing the model on small size data and as such obtaining points to be used in curve fitting, and subsequent forecasting of the required sample

size. Although the review is very thorough and comprehensive, its findings cannot necessarily generalize to the general case of ML applications on non-image data. Nevertheless, they illustrate the scarcity of sound and robust methodological approaches for sample size determination for ML applications, and the need for further development.

Details on the population datasets

The following are the details for the datasets that were used in this study:

Canadian COVID-19

The first dataset is the Canadian COVID-19 dataset from the Public Health Agency of Canada [S1]. It contains over 1 million health records of individuals who have tested positive for COVID-19. We are interested in fitting a model that predicts mortality caused by COVID-19. The binary outcome of interest is derived from the case status in the dataset, and a value of 1 is assigned if the patient has died due to COVID-19 while a value 0 is assigned if the patient has recovered. The selected predictors for modeling include the following variables: date, age group, gender, region, exposure, province. Table S2 presents an overview of the variables that are included in the binary model.

Canadian Community Health Survey

The CCHS data is a cross-sectional telephone survey administered by Statistics Canada that collects information on health status, health care utilization and health determinants of Canadians [S2]. This dataset is a pooled version of survey data from 2001 to 2013, and variables we are using are presented in Table S3. The model outcome is cardiovascular health and the covariates are age, sex, education, house income, household size, immigration as predictors to predict the ideal state of cardiovascular health using variables from the dataset [S41]. To assess cardiovascular health, we follow the definition of ideal cardiovascular health introduced by the American Heart Association to calculate the Cardiovascular Health in Ambulatory Care Research Team (CANHEART) health index score, which is determined by 7 health factors including smoking, obesity, hypertension, diabetes, physical activity, and fruit and vegetable consumption [S42]. The final CANHEART index score ranges from 0 (worst) to 6 (best). The outcome is assigned to be 1 if the score is above 3.5 [S43], which is considered to be an intermediate or ideal state of cardiovascular health, and 0 otherwise.

COVID-19 Survival

The COVID-19 survival dataset [S3] that is used in the study is a web-based survey data collected by the research team by Nexoid, a company in United Kingdom. They collect demographic, socioeconomic and health-related information of individuals to predict two crucial aspects related to COVID-19: the probability of being infected with COVID-19 as well as probability of mortality associated with COVID-19. In our study, we focus on the probability of COVID-19 infection using the important demographic, behavioral and health factors including age, sex, race, smoking, nursing home, COVID-19 symptoms, COVID-19 contact, health worker, and the presence of comorbidities such as asthma, kidney disease, liver disease, heart disease, lung disease, diabetes, and hypertension. The outcome of interest is determined by the risk scores of getting infected with COVID-19. The patients whose risk scores exceed the mean risk score are considered as having a high risk of contracting COVID-19, while those with scores below the mean are classified as having a low risk. Table S4 summarizes the basic statistics of the selected variables.

FDA Adverse Events

The next dataset contains the reports submitted to the FDA Adverse Event Reporting System for patients with adverse events [S4]. The binary outcome of interest for this dataset is whether or a patient has died. Our primary goal with this dataset is to explore the relationship between the patient mortality and various predictors, including event date, gender, age, weight, drug name and the indication for drug use. Detailed statistics for these variables can be found in Table S5.

Texas Inpatients (2012)

Texas inpatient dataset [S5] contains 75 variables. Similar to the Washington state hospital discharge data, in this dataset, we explore the relationship between those demographic and health factors and the length of stay in the Texas hospitals. The involved covariates include age, sex, race, ethnicity, location,

weekday, risk mortality, severity, DRG and fees with detailed description in Table S6. According to their length of stay in the hospital, the patients are classified into two groups, and the outcome is assigned with a value of 1 if the patient's length of stay is greater than or equal to 3 days and 0 otherwise.

Washington State Hospital Discharges (2007)

The Washington State Hospital Discharge dataset [S6] contains over 350 variables. Among these, we model the relationship between those demographic and health factors and the length of stay in the hospital. The covariates were: age, atype, aweekend, died, DRG, primary diagnosis code, and ZIP code. A detailed description of these variables is displayed in Table S7. The outcome of our study categories patients into two groups based on their length of stay. A value of 1 is assigned if the patient's length of stay is greater than or equal to 3 days and 0 otherwise.

Basic Stand Alone (BSA) Inpatient Claims

This dataset [S7] contains the claim-level information with each recording being an inpatient claim chosen from a 5% random sample of Medicare beneficiaries during 2008. In this study, we choose the variables including age, gender, DRG, ICD-9 primary procedure code, Medicare payment and the length of stay and explore the relationship between the length of stay and its relevant demographic and claim-related factors. The outcome is defined as a binary variable taking a value of 1 if the length of stay on the file is greater than or equal to 2.5 days, and 0 otherwise. Table S8 provides an overview of the detailed statistics for these variables.

Washington State Hospital Discharges (2008)

This dataset [S6] contains 652,340 inpatient discharge records in 2008 from community hospitals in Washington from State Inpatient Databases that are used to track the trends in healthcare utilization, access, charges, quality and outcomes in United States. We are interested in examining the relationship between length of stay and its demographic and health factors. Specifically, the covariates of interest include age, female, race, admission type, aweekend, DRG, DX1, primary payer, total charges, zip code, chronic conditional indicators, and procedure classes for ICD-10-PCS procedure codes, comorbidity measures for alcohol abuse, depression, hypertension and obesity. The outcome is created by classifying the patients into two groups based on the median of their length of stay. A value of 1 is assigned if the patient's length of stay is greater than or equal to 2 days and 0 otherwise. Detailed statistics of the variables are displayed in Table S9.

California Hospital Discharges (2008)

This dataset [S6] contains over 4 million inpatient discharge records in 2008 from community hospitals in California from State Inpatient Databases that are used to track the trends in healthcare utilization, access, charges, quality and outcomes in United States. We are interested in exploring the relationship between length of stay and its demographic and health factors. Specifically, the covariates of interest include age, female, race, aweekend, DRG, DX1, primary payer, total charges, chronic conditional indicators, and procedure classes for ICD-10-PCS procedure codes, comorbidity measures for alcohol abuse, depression, hypertension and obesity. The outcome is generated by dividing the patients into two groups based on the median of their length of stay. A value of 1 is assigned if the patient's length of stay is greater than or equal to 3 days and 0 otherwise. Detailed statistics of the variables are displayed in Table S10.

Florida Hospital Discharges (2007)

This dataset [S6] contains over 2.3 million inpatient discharge records in 2007 from community hospitals in Florida from State Inpatient Databases that are used to track the trends in healthcare utilization, access, charges, quality and outcomes in United States. We are interested in exploring the relationship between length of stay and its demographic and health factors. Specifically, the covariates of interest

include age, female, race, admission type, aweekend, DRG, DX1, primary payer, total charges and zip code. The outcome is created by classifying the patients into two groups based on the median of their length of stay. A value of 1 is assigned if the patient's length of stay is greater than or equal to 3 days and 0 otherwise. Detailed statistics of the variables are displayed in Table S11.

New York Hospital Discharges (2007)

This dataset [S6] consists of over 4.6 million inpatient discharge records in 2007 from community hospitals in New York from State Inpatient Databases that are used to track the trends in healthcare utilization, access, charges, quality and outcomes in United States. We are interested in examining the relationship between length of stay and demographic and health factors. Specifically, the covariates of interest include age, female, race, admission type, aweekend, DRG, DX1, primary payer, total charges, zip code, chronic conditional indicators and procedure classes for ICD-10-PCS procedure codes. The outcome is created by classifying the patients into two groups based on the median of their length of stay. A value of 1 is assigned if the patient's length of stay is greater than or equal to 3 days and 0 otherwise. Detailed statistics of the variables are displayed in Table S12.

Better Outcomes Registry & Network

Data are collected from BORN Ontario birth registry [S8] that covers about 1 million records regarding Ontario's maternal demographic characteristics, obstetrical history, health behaviors, prenatal screening and newborn care information. We combine the pregnancy and infant datasets and examine the association between low birthweight and its related risk factors. The relevant factors include as gestational age, maternal age, maternal body mass index, total number of pregnancies a mother has experienced, number of previous preterm pregnancies, number of previous abortions, maternal smoking status, alcohol exposure, prenatal screening, mental health concerns for addiction, anxiety, depression, maternal health conditions for diabetes and genetics and drug exposure to Cocaine, Hallucinogens and Opioids. We follow the definition of low birthweight[25] and classify the newborns whose birth weights are less than 2,500 grams as infants with low birthweight. A value of 1 is given for newborns with low birthweight and 0 otherwise. A summary of descriptive statistics for the variables is presented in Table S13.

Medical Information Mart for Intensive Care III

The dataset [S9] is extracted from MIMIC-III relational database (version 1.4), which contains deidentified clinical data of the patients who were admitted to the Beth Israel Deaconess Medical Center in Boston, Massachusetts [S9,S44,S45]. It contains various tables of patient's data regarding the demographics, admission information, lab tests, diagnosis codes, caregiver information, discharge notes. We use this dataset to investigate the relationship between 30-day readmission and its related demographics, vital signs and lab test values. The demographics include the age of the patients when they were first admitted to the ICU, their ethnicity group and admission type. The vital signs consider the (systolic and diastolic) blood pressure, heart rate and respiration rate. Several lab measurements are also incorporated into the analysis. The selection criteria for readmitted patients is to include those who were readmitted within 30-day of initial hospital discharge from the ICU. The patients who were readmitted to the ICU are assigned a label of 1, while those who were not readmitted are assigned a label of 0. Table S14 summarizes the descriptive statistics of the selected variables.

Sensitivity Analysis of Sample Size Calculation to Training Dataset

We investigated the sensitivity of the sample size calculator to the datasets that were used to train it. Tables S15-S18 show the results of the certainty curve sample size estimation performance on our two evaluative metrics, mRE and mALQE, when removing each individual dataset completely from training at different values of lambda and certainty. For example, the CCHS row indicates the performance value when the CCHS is removed from the training datasets. The performance is still calculated using leave-one-dataset-out for the remaining 12 datasets after removing each dataset from training. The default value at the bottom of the table shows the performance value with all of the datasets included in the calculation.

Pseudocode process for the sample size calculator function

The following pseudocode describes the algorithm implanted in the R code found in [S46].

Algorithm: EstimateRequiredSampleSize

Goal:
Estimate the minimum sample size needed to achieve a desired certainty level using a predictive model and smoothed certainty estimates.

Inputs:

- model: a trained predictive model (e.g., LightGBM)
- certainty_threshold: target certainty level (default = 0.8)
- degrees_of_freedom: model complexity indicator
- entropy: average entropy of the data
- imbalance: class imbalance factor

Output:

- required_sample_size: the estimated sample size needed
- achieved_certainty: the model's predicted certainty at that sample size

Steps:

1. **Initialize Parameters**
    - Define a range of candidate sample sizes.
    - Compute their logarithmic values for modeling.
2. **Predict Certainty Across Sample Sizes**
    - Use the model to estimate certainty for each candidate sample size, given fixed values of entropy, imbalance, and degrees of freedom.
3. **Determine Relevant Sample Size Range**
    - If the predicted certainty varies meaningfully:
        - Identify the central region where certainty transitions.
        - Expand this region to include context before and after the transition.
        - Exclude sample sizes that are too small.
    - Otherwise, use the full range of sample sizes.
4. **Refine Certainty Estimates**
    - Recompute certainty predictions over the refined sample size range using a secondary model.

- Apply a smoothing function (e.g., loess) to the logit-transformed certainty values.

5. **Find Minimum Sample Size Meeting Certainty Threshold**
    - Identify the smallest sample size where the smoothed certainty exceeds the threshold.
    - If none found, default to the smallest candidate.
6. **Return Results**
    - Convert the selected log sample size back to its original scale.
    - Return both the required sample size and the corresponding certainty.

References

1. Esri Canada (2023). Canadian health records of COVID-19 gathered by the Public Health Agency of Canada. Available at: https://resources-covid19canada.hub.arcgis.com/
2. Canadian Community Health Survey (2021). Statistics Canada. Available at: https://www150.statcan.gc.ca/n1/pub/82-620-m/2005001/4144189-eng.htm
3. Nexoid (2021). COVID-19 survival dataset. Available at: https://www.covid19survivalcalculator.com/en/download
4. FDA Adverse Event Reporting System (2018). Database comprising information on adverse event and medication error reports submitted to FDA. Available at: https://open.fda.gov/data/faers/
5. Texas Inpatient Public Use Data File (2025). Patient hospital discharge information from Texas hospitals. Available at: https://www.dshs.texas.gov/center-health-statistics/chs-data-sets-reports/texas-health-care-information-collection/health-data-researcher-information/texas-inpatient-public-use
6. Healthcare Cost and Utilization Project (HCUP), Agency for Healthcare Research and Quality (2025). California, Florida, New York, and Washington State Inpatient Databases (SID). Available at: https://hcup-us.ahrq.gov/tech_assist/centdist.jsp
7. Centers for Medicare & Medicaid Services (CMS) (2025). Basic Stand Alone (BSA) Inpatient Claims Public Use File (PUF). Available at: https://www.cms.gov/data-research/statistics-trends-and-reports/basic-stand-alone-medicare-claims-public-use-files/bsa-inpatient-claims-puf
8. Better Outcomes Registry & Network (BORN) Ontario (2021). Data Resource Profile: Better Outcomes Registry & Network (BORN) Ontario. Available at: https://academic.oup.com/ije/article/50/5/1416/6294519
9. Johnson, A.E.W., Pollard, T.J., Shen, L., Lehman, L.H., Feng, M., Ghassemi, M., Moody, B., Szolovits, P., Celi, L.A., and Mark, R.G. (2016). MIMIC-III, a freely accessible critical care database. Scientific Data 3, 160035. Available at: https://doi.org/10.1038/sdata.2016.35
10. Heart Disease (1989). Dataset for predicting heart disease presence. Available at: https://doi.org/10.24432/C52P4X; also available at: https://doi.org/10.17605/OSF.IO/7BS8Q
11. Breast Cancer Wisconsin (1993). Breast cancer diagnostic dataset. Available at: https://doi.org/10.24432/C5DW2B; also available at: https://doi.org/10.17605/OSF.IO/7BS8Q
12. Chronic Kidney Disease (2015). Kidney disease prediction dataset. Available at: https://doi.org/10.24432/C5G020; also available at: https://doi.org/10.17605/OSF.IO/7BS8Q
13. Breast Cancer Coimbra (2018). Diagnostic Coimbra breast cancer dataset. Available at: https://doi.org/10.24432/C52P59; also available at: https://doi.org/10.17605/OSF.IO/7BS8Q
14. Breast Cancer (1988). Breast cancer prediction dataset. Available at: https://doi.org/10.24432/C51P4M; also available at: https://doi.org/10.17605/OSF.IO/7BS8Q

15. EEG Eye State (2013). EEG measurement dataset. Available at: https://doi.org/10.24432/C57G7J; also available at: https://doi.org/10.17605/OSF.IO/7BS8Q

16. Adult (1996). Adult income classification dataset. Available at: https://doi.org/10.24432/C5XW20; also available at: https://doi.org/10.17605/OSF.IO/7BS8Q

17. Bank note (2012). Bank note dataset. Available at: https://doi.org/10.24432/C55P57; also available at: https://doi.org/10.17605/OSF.IO/7BS8Q

18. Titanic Survival (1912). Titanic survival prediction dataset. Available at: https://www.kaggle.com/datasets/hesh97/titanicdataset-traincsv; also available at: https://doi.org/10.17605/OSF.IO/7BS8Q

19. Stroke (2023). Stroke Prediction dataset. Available at: https://doi.org/10.17605/OSF.IO/7BS8Q

20. Colposcopy/green (2017). Digital Colposcopy (green) dataset. Available at: https://doi.org/10.24432/C5C022; also available at: https://doi.org/10.17605/OSF.IO/7BS8Q

21. Colposcopy/hinselmann (2017). Digital Colposcopy (hinselmann) dataset. Available at: https://doi.org/10.24432/C5C022; also available at: https://doi.org/10.17605/OSF.IO/7BS8Q

22. Colposcopy/schiller(2017). Digital Colposcopy (schiller) dataset. Available at: https://doi.org/10.24432/C5C022; also available at: https://doi.org/10.17605/OSF.IO/7BS8Q

23. Thoracic Surgery (2014). Post-operative life expectancy classification prediction dataset. Available at: https://doi.org/10.24432/C5Z60N; also available at: https://doi.org/10.17605/OSF.IO/7BS8Q

24. Diabetic Retinopathy Debrecen (2014). Diabetic retinopathy prediction dataset. Available at: https://doi.org/10.24432/C5XP4P; also available at: https://doi.org/10.17605/OSF.IO/7BS8Q

25. Z-Alizadeh Sani (2013). Coronary artery disease prediction dataset. Available at: https://doi.org/10.24432/C5Q31T; also available at: https://doi.org/10.17605/OSF.IO/7BS8Q

26. Pima Indians Diabetes (1988). Diabetes dataset from Pima Indians. Available at: https://doi.org/10.17605/OSF.IO/7BS8Q

27. Figueroa, R.L., Zeng-Treitler, Q., Kandula, S., and Ngo, L.H. (2012). Predicting sample size required for classification performance. BMC Med Inform Decis Mak *12*, 8. Available at: https://doi.org/10.1186/1472-6947-12-8.

28. Mukherjee, S., Tamayo, P., Rogers, S., Rifkin, R., Engle, A., Campbell, C., Golub, T.R., and Mesirov, J.P. (2003). Estimating Dataset Size Requirements for Classifying DNA Microarray Data. Journal of Computational Biology *10*, 119–142. Available at: https://doi.org/10.1089/106652703321825928.

29. Raudys, S.J., and Jain, A.K. (1990). Small sample size effects in statistical pattern recognition: recommendations for practitioners and open problems. In [1990] Proceedings. 10th International Conference on Pattern Recognition (IEEE Comput. Soc. Press), pp. 417–423. Available at: https://doi.org/10.1109/ICPR.1990.118138.

30. Fukunaga, K., and Hayes, R.R. (1989). Effects of sample size in classifier design. IEEE Trans. Pattern Anal. Machine Intell. *11*, 873–885. Available at: https://doi.org/10.1109/34.31448.

31. Larracy, R., Phinyomark, A., and Scheme, E. (2021). Machine Learning Model Validation for Early Stage Studies with Small Sample Sizes. In 2021 43rd Annual International Conference of the IEEE Engineering in Medicine & Biology Society (EMBC) (IEEE), pp. 2314–2319. Available at: https://doi.org/10.1109/EMBC46164.2021.9629697.

32. van der Ploeg, T., Austin, P.C., and Steyerberg, E.W. (2014). Modern modelling techniques are data hungry: a simulation study for predicting dichotomous endpoints. BMC Medical Research Methodology *14*, 137. Available at: https://doi.org/10.1186/1471-2288-14-137.

33. Vabalas, A., Gowen, E., Poliakoff, E., and Casson, A.J. (2019). Machine learning algorithm validation with a limited sample size. PLOS ONE *14*, e0224365. Available at: https://doi.org/10.1371/journal.pone.0224365.

34. McNamara, M.E., Zisser, M., Beevers, C.G., and Shumake, J. (2022). Not just "big" data: Importance of sample size, measurement error, and uninformative predictors for developing prognostic models for digital interventions. Behaviour Research and Therapy *153*, 104086. Available at: https://doi.org/10.1016/j.brat.2022.104086.

35. Zantvoort, K., Nacke, B., Görlich, D., Hornstein, S., Jacobi, C., and Funk, B. (2024). Estimation of minimal data sets sizes for machine learning predictions in digital mental health interventions. npj Digit. Med. *7*, 1–10. Available at: https://doi.org/10.1038/s41746-024-01360-w.

36. Acharjee, A., Larkman, J., Xu, Y., Cardoso, V.R., and Gkoutos, G.V. (2020). A random forest based biomarker discovery and power analysis framework for diagnostics research. BMC Med Genomics *13*, 178. Available at: https://doi.org/10.1186/s12920-020-00826-6.

37. D'souza, R.N., Huang, P.-Y., and Yeh, F.-C. (2020). Structural Analysis and Optimization of Convolutional Neural Networks with a Small Sample Size. Sci Rep *10*, 834. Available at: https://doi.org/10.1038/s41598-020-57866-2.

38. Bartlett, P.L., Harvey, N., Liaw, C., and Mehrabian, A. (2017). Nearly-tight VC-dimension and pseudodimension bounds for piecewise linear neural networks. Preprint at arXiv, https://arxiv.org/abs/1703.02930

39. Beleites, C., Neugebauer, U., Bocklitz, T., Krafft, C., and Popp, J. (2013). Sample size planning for classification models. Analytica Chimica Acta *760*, 25–33. Available at: https://doi.org/10.1016/j.aca.2012.11.007.

40. Balki, I., Amirabadi, A., Levman, J., Martel, A.L., Emersic, Z., Meden, B., Garcia-Pedrero, A., Ramirez, S.C., Kong, D., Moody, A.R., et al. (2019). Sample-Size Determination Methodologies for Machine Learning in Medical Imaging Research: A Systematic Review. Can Assoc Radiol J *70*, 344–353. Available at: https://doi.org/10.1016/j.carj.2019.06.002.

41. Azizi, Z., Lindner, S., Shiba, Y., Raparelli, V., Norris, C.M., Kublickiene, K., Herrero, M.T., Kautzky-Willer, A., Klimek, P., Gisinger, T., et al. (2023). A comparison of synthetic data generation and federated analysis for enabling international evaluations of cardiovascular health. Sci Rep *13*, 11540. Available at: https://doi.org/10.1038/s41598-023-38457-3.

42. Lloyd-Jones, D.M., Hong, Y., Labarthe, D., Mozaffarian, D., Appel, L.J., Van Horn, L., Greenlund, K., Daniels, S., Nichol, G., Tomaselli, G.F., et al. (2010). Defining and Setting National Goals for Cardiovascular Health Promotion and Disease Reduction. Circulation *121*, 586–613. Available at: https://doi.org/10.1161/CIRCULATIONAHA.109.192703.

43. Maclagan, L.C., Park, J., Sanmartin, C., Mathur, K.R., Roth, D., Manuel, D.G., Gershon, A., Booth, G.L., Bhatia, S., Atzema, C.L., et al. (2014). The CANHEART health index: a tool for monitoring the cardiovascular health of the Canadian population. CMAJ *186*, 180–187. Available at: https://doi.org/10.1503/cmaj.131358.

44. Goldberger, A.L., Amaral, L.A.N., Glass, L., Hausdorff, J.M., Ivanov, P.Ch., Mark, R.G., Mietus, J.E., Moody, G.B., Peng, C.-K., and Stanley, H.E. (2000). PhysioBank, PhysioToolkit, and PhysioNet: Components of a New Research Resource for Complex Physiologic Signals. Circulation *101*. Available at: https://doi.org/10.1161/01.CIR.101.23.e215.

45. Johnson, A., Pollard, T., and Mark, R. (2016). MIMIC-III Clinical Database (version 1.4). (PhysioNet). Available at: https://doi.org/10.13026/C2XW26.

46. Mitsakakis, N., Liu, D., and El Emam, K. Sample Size Calculation for Training Ensemble Machine Learning Models on Health Data. OSF. Available at: https://doi.org/10.17605/OSF.IO/7BS8Q
