# Supplementary material for: Sample size calculation for training ensemble machine learning models on health data
Source: Patterns (N Y). 2026 Mar 26;7(6):101498. doi: 10.1016/j.patter.2026.101498 (PMC13280678; doi:10.1016/j.patter.2026.101498)
Supplement: Document S2. Article plus supplemental information [file mmc2.pdf]

Article

Patterns

Sample size calculation for training ensemble machine learning models on health data

Highlights

- A conceptual model for thinking about power in prediction models has been developed
- A sample size estimator for ensemble tree-based ML models has been fitted
- The estimator is significantly more accurate than previously proposed methods
- The estimator can be used for sample size calculations for clinical prediction models

Authors

Nicholas Mitsakakis, Dan Liu, Thomas Walters, Khaled El Emam

Correspondence

kelemam@ehealthinformation.ca

In brief

Most clinical prediction modeling studies using machine learning models do not estimate sample size. A conceptual model for thinking about power for machine learning models is presented in this study, and an estimator has been fitted for sample size calculations during study design.

Mitsakakis et al., 2026, Patterns 7, 101498
June 12, 2026 © 2026 The Authors. Published by Elsevier Inc.
https://doi.org/10.1016/j.patter.2026.101498

CellPress

Patterns

CellPress OPEN ACCESS

Article

Sample size calculation for training ensemble machine learning models on health data

Nicholas Mitsakakis,[1] Dan Liu,[1,2] Thomas Walters,[3] and Khaled El Emam[1,2,4,*]
[1]CHEO Research Institute, Ottawa, ON, Canada
[2]School of Epidemiology and Public Health, Faculty of Medicine, University of Ottawa, Ottawa, ON, Canada
[3]Division of Gastroenterology, Hospital for Sick Children, Toronto, ON, Canada
[4]Lead contact
*Correspondence: kelemam@ehealthinformation.ca
https://doi.org/10.1016/j.patter.2026.101498

THE BIGGER PICTURE Machine learning (ML) is transforming healthcare by enabling predictive models that can guide clinical decisions, improve patient outcomes, and optimize resource allocation. Most clinical ML predictive studies, however, overlook a critical design element: determining the quantity of data needed to train a reliable model. Without adequate sample size planning, studies risk producing unstable models that fail to generalize, wasting resources and potentially leading to erroneous conclusions. This gap is especially concerning as ML becomes integral to regulatory submissions and clinical practice.

Our work introduces an empirically derived sample size calculator for ensemble ML models—random forests and gradient-boosted trees—trained on tabular health data. By modeling the relationship between sample size and predictive performance across diverse real-world datasets, we provide a practical tool that moves beyond outdated heuristics and inappropriately used statistical formulas, offering a method suitable for ML model complexity. This framework can improve study design, enhance transparency and reproducibility, and inform policy and reporting standards. Looking ahead, the methodology can be adapted to other domains where ML is applied to structured data, such as finance, education, and public policy. Ultimately, better sample size planning will lead to more trustworthy AI systems, fostering innovation while safeguarding patient and societal interests.

SUMMARY

Health research studies often suffer from small sample sizes, and training machine learning (ML) models requires large datasets. There is a dearth of literature on determining the adequate sample size for using ML models. We developed an empirically derived sample size calculator for ensemble ML models: random forests and two gradient-boosted decision trees (light gradient boosting machine [LGBM] and extreme gradient boosting [XGBoost]). This predicts the sample size required to achieve a pre-defined level of prognostic performance with a certain probability. Prognostic performance is defined as the sample area under the ROC curve (ROC-AUC) relative to the optimal model trained on the full (population) dataset. Our calculator's accuracy was compared to three common heuristics and a statistical approach to sample size calculation. For example, the median relative error sample size prediction was 25% to achieve 85% of the optimal performance with 90% certainty for LGBM. Our model has significantly better accuracy than other methods for tree-based ensemble ML models.

INTRODUCTION

Clinical research studies often have small dataset sizes.[1] Conducting a study with a sample size that is deemed to be too small can lead to model instability[2] especially of predictor effects,[3] as well as overfitting and an inability to generalize predictions to unseen data[4,5] even under ideal conditions (e.g., no data shift or drift). This can mean that the study is unable to meet its goals and objectives, and the resources and patient burden expended in collecting the data may not produce meaningful results, which is wasteful. In addition, the ethics of enrolling patients in studies that will not answer the research question(s) are tenuous.[6] Sample size determination during study design is therefore essential to ensure that studies have sufficient observations to adequately answer the research question.

© 2026 The Authors. Published by Elsevier Inc.
This is an open access article under the CC BY license (http://creativecommons.org/licenses/by/4.0/).

When the study employs traditional statistical methodologies, sample size calculation can be done using well-established methods.[7] However, sample size calculation methods have not been established for machine learning (ML) models, which are increasingly applied in clinical research studies.[8,9] While it is acknowledged that ML methods require large datasets to achieve optimal predictive performance,[4,10] it is unclear what the exact sample size should be to give such optimal and stable model performance and whether the sample size requirements vary by ML method. This poses a significant problem for researchers.

Previous studies that examined ML sample size used existing collected data to fit a power curve and then used that to estimate performance improvements if additional data are collected or additional samples are annotated.[11,12] However, this would not be applicable before data collection has started (i.e., during study design). Others make strong assumptions about the data, for example, normality of the predictor variables,[13–16] which would not likely be met in many health datasets. While sample size estimation studies have been performed for image data[17,18] and spectroscopy data,[19] these data modalities are not applicable to our context, which is tabular health data. One study examined the impact of sample size and feature selection on random forests; however, in that case, the concept of power was not defined in terms of prognostic performance.[20] Another study on sample size reported optimism, which is how well the model performed in the training data in comparison to the test data.[4] In addition, many of these studies (1) did not tune the ML models, and therefore, it is not clear to what extent their results can be applied to the more practical scenario where model tuning is applied, and (2) examined a small range of sample sizes. More details on these studies are included in the supplemental information.

The vast majority of prognostic clinical research studies utilizing ML methods do not report a sample size calculation[3,9,21,22] and very often rely on convenience sample sizes of available data, avoiding any discussion about the sufficiency of the data for developing an accurate and stable model. Some ML studies rely on precedents for sample size determination, which by themselves oftentimes cannot be justified. Alternatively, oversimplified "rule of thumb" practices borrowed from the statistical literature are often used, such as the "10 events per predictor,"[23,24] "15 events per variable" (often used in prediction models[25]), and "300 events per variable."[4] The former two are consistent with the median number of 12.5 events per predictor found in the literature.[9]

Approaches found in the statistical literature may be used. For example, one strategy[26] for calculating the required sample size relies on mathematical formulas and empirical approximations[23] that are applicable to statistical models, such as linear, logistic, or Cox regression. These formulas and calculations are not necessarily relevant for general ML models because they have significant differences from traditional statistical regression methods in the way they work and the complexity of the relationships that they can capture. However, these methods are still used to estimate the sample size for ML models, despite having potentially nontrivial errors.[27,28]

Finally, there are methodologies for the estimation of the required sample size for studies aiming to clinically validate previously trained and developed ML models.[29] These approaches assume the existence of already trained ML models, and they focus on the effect of the sample size of new data needed for precisely estimating measures of the model's performance as part of the validation objective. As such, their goal is distinct from estimating sample size for developing and training a new ML model, and therefore, they cannot be applied to our objectives.

In this study, we develop a sample size calculator with the following characteristics: sample size calculations that would be performed *a priori*, before data collection, (1) for training an ML model and (2) for ensemble binary classification ML modeling methods that will be trained on a structured tabular dataset for prognostic purposes and (3) that would perform better than the available heuristic and statistical methods.

The specific ensemble ML methods that we consider are random forests and gradient-boosted decision trees.

METHODS

Our methodology consists of performing a retrospective simulation to model the relationship between sample size and ML model performance.

Simulation design

We used large real datasets as "population" datasets. Then, we trained, tuned, and assessed three tree-based ensemble ML models, obtaining the "optimal" performance for these classifiers on the population datasets. Subsequently, we sampled data with varying sizes from the population datasets and used them for training, tuning, and testing the ML models. The performance of these sample-trained models was then compared with the "optimal" performance, indicating the impact of a smaller sample size relative to the optimal performance. An overview of the overall process is shown in Figure 1.

Datasets

We used 13 large real-world health datasets[30–38] to represent different populations. These datasets covered multiple domains and settings, including public health, health surveys, hospital discharge, intensive care unit (ICU) visits, adverse events, and specific population registries. The heterogeneity of complexity in these datasets was deliberate to enable generalization to a broader set of predictive clinical research scenarios. Being real datasets, they embodied a realistic mix of continuous and categorical variables, non-linearities, correlational structure, measurement error, possible noise variables, potential labeling errors for the outcome, and various levels of outcome imbalance. These are all factors that are known to have an impact on ML model performance and influence the relationship between sample size and performance.[39–41]

A summary of these datasets is provided in Table 1, with more details about each dataset provided in Tables S2–S14. For each dataset, a binary classification model was defined, and these are described in the supplemental information. The number of variables used in each classification model is shown in the last column of Table 1. In addition, the table shows the original number of observations and the number of observations after removing those with any missing values on the outcome variable.

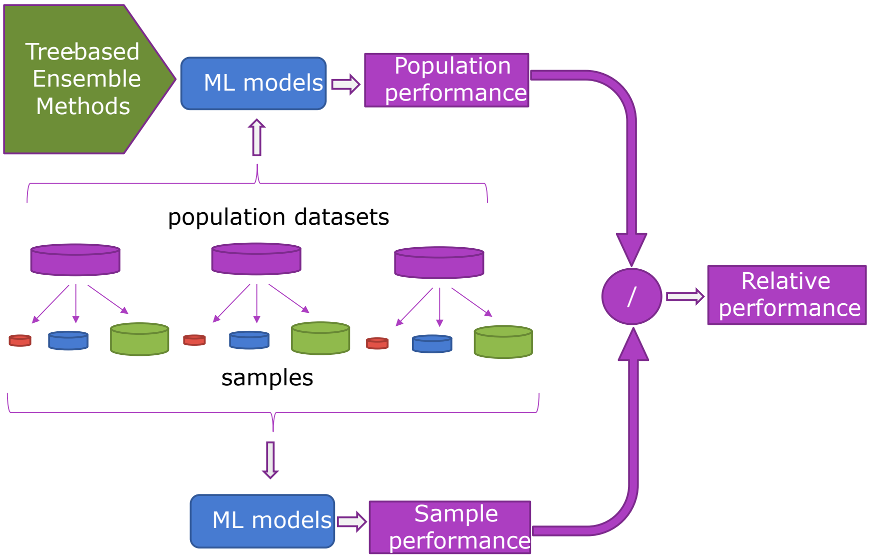

Figure 1. Diagram illustrating the process of our computational experiments

Evaluation approach

Every population dataset was randomly split into 70% training and 30% test datasets. For each population dataset, the same testing partition was used to evaluate all models trained using that dataset. This ensured that the results from all models, irrespective of the sample size that was used to train them and irrespective of the type of ML algorithm, would be evaluated on the same test set, which allows consistent comparability across all models.

The area under the ROC curve (ROC-AUC) was used to assess the performance of the binary classification models. ROC-AUC is a measure of discrimination, which is an important aspect of the performance of an ML model, and it is widely used in similar studies because it can be used to compare performance across different studies and datasets. Other measures, such as the Brier score (measuring both discrimination and calibration), do not share similar properties, as they depend on the particular dataset, and they can mainly be used for comparing the performance of models trained on the same dataset. For instance, the Brier score depends inherently on the prevalence of the outcome, posing interpretation challenges when used across different datasets.[42,43]

Sampling from population data

Samples were drawn from each of the training partitions, using different sample sizes. The sizes of the samples were based on a geometric series. Define $b \sim N(\mu = 1.5, \sigma^2 = 0.005^2)$, and the series would be $n_i = [b^{i+9}], i = 1, \ldots, 23$, where $[x]$ denotes rounding to the closest integer to x. The series would be stopped if a value exceeded the training partition size for a particular dataset.

The justification behind the choice of geometric series is based on the assumption and expectation that changes in a model's performance (on ROC-AUC) diminish as the sample size increases (i.e., the performance will plateau). 100 series were generated for each dataset, and for each value in a series, a random sample of that size was drawn from each training dataset. Therefore, for each dataset, there were 2,300 models trained with varying sample sizes. Each series is randomized so that we can have sample sizes across the range rather than at fixed points, ensuring smoothness in the modeling described below.

ML methods

Our investigation focuses on tree-based ensemble ML methods: random forests and gradient boosting decision trees (LightGBM and XGBoost). Tree-based models are the most common type of ML prognostic methods used in clinical research[9]; they perform better than linear models, such as logistic regression,[44–48] and have also been found to perform better than deep learning models on tabular datasets.[49,50]

Model tuning used 5-fold cross-validation. All models were tuned using Bayesian optimization.[51] The range for the tuning parameters, specific for each model, was previously suggested[52–54] and is detailed in Table S1. High-cardinality variables were converted to embeddings[55] using a scheme similar to target encoding. The sdgm R package that implements this functionality is available online.[56]

Model specification for observed sample size

Concept of certainty curves

Previous studies assessed the pseudo-power of an ML model by examining the relationships between predictors and the outcome[20] or used the power of the ROC-AUC calculation itself.[39] We sought to develop a direct approach that is more easily interpretable by analysts when designing their studies.

Here, we describe a new approach that can be used for measuring and assessing the adequacy of the size of a dataset to be used for training ML models. The adequacy is characterized based on the estimated performance of the model(s) training on the particular dataset, as it compares with the hypothetical maximum (or optimal) performance of the model(s) when applied to the population from which the dataset is sampled. We are making the assumption that the performance of the model is monotonically improving on average as the sample size increases, and it reaches its maximum under the population from which the dataset is sampled.

Since the estimated performance on the dataset is stochastic, we adopt a probabilistic framework. We describe certainty curves whereby the analyst already knows which ML modeling technique will be used and wishes to determine the sample size to use for training that ML model.

While the description below is for ROC-AUC, similar reasoning applies to other model performance measures that can be used across different datasets and studies. Under this setting, certainty curve $C(n)$ is a mathematical function from N^* to (0,1), given by

$$C(n) = Pr_{|S| = n, S \subset P}\left(AUC(S) \geq t_s \middle| AUC(P) \geq t_p\right), \quad \text{(Equation 1)}$$

Table 1. A description of the complete datasets used in the analyses

Dataset (dataset label)	Description of dataset	No. observations (original)	No. observations[a]	Variables used in analysis
COVID[30] (COVID)	COVID-19 health records of Canadians collected by Esri Canada	1,384,881	745,623	7
Canadian Community Health Survey[31] (CCHS)	a pooled version of survey data across multiple years that gathers health information for Canadian population	904,813	752,472	8
COVID Survival[32] (Nexoid)	a secondary web-based survey dataset concerning COVID-19 survival prediction collected by the Nexoid company in London, UK	968,408	968,394	19
FDA Adverse Event Reporting System[33] (FAERS)	adverse event and medication error reports submitted to FDA	881,204	251,409	7
Texas Inpatient Data[34] (TEXAS)	discharges from Texas hospitals	745,999	745,997	11
Washington State Hospital Discharge[35] (WASHINGTON2007)	hospital discharge information from the HCUP state inpatient database for 2007	644,902	644,901	8
Basic Stand Alone (BSA) Inpatient Claims[36]	claim-level information from 2008 Medicare inpatient claims	588,415	588,415	6
Washington State Hospital Discharge[35] (WASHINGTON2008)	hospital discharge information from the HCUP state inpatient database for 2008	652,340	652,340	18
California Hospital Discharge[35] (CALIFORNIA2007)	hospital discharge information from the HCUP state inpatient database for 2007	4,016,573	4,016,573	16
Florida Hospital Discharge[35] (FLORIDA2007)	hospital discharge information from the HCUP state inpatient database for 2007	2,327,563	2,327,563	12
New York Hospital Discharge[35] (NEWYORK2007)	hospital discharge information from the HCUP state inpatient database for 2007	4,666,541	4,666,541	14
Better Outcomes Registry & Network[37] (BORN)	a registry that contains comprehensive perinatal, newborn, and child information in Ontario	963,083	963,083	18
Medical Information Mart for Intensive Care III[38] (MIMIC-III)	health-related information for patients who stayed in critical care units of the Beth Israel Deaconess Medical Center between 2001 and 2012	540,482	540,482	10

[a]After data transformation/removing missing values on the outcome variable only.

where S denotes a random sample from a population dataset, $|S|$ is the size of S, $ROC\text{-}AUC(X)$ denotes the value of the ROC-AUC for the model trained on dataset X, t_s and t_p are threshold parameters for ROC-AUC values for models trained on a sample and on the population, respectively, and P is the population.

$C(n)$ is giving the probability for the specific ML model to have ROC-AUC performance of at least equal to t_s when trained on a dataset S, which has sample size n from a population P, given that the model gives a ROC-AUC at least equal to t_p when trained on this population P. In practice, $t_s \leq t_p$. For example, for values $t_s = 0.75$ and $t_p = 0.8$ and assuming that the modeling method is random forest, $C(n)$ gives the probability that a random forest model trained on a sample S with size n, from a specific population P, will have performance with a ROC-AUC ≥ 0.75, assuming that the model's performance based on the population is given by a ROC-AUC ≥ 0.8.

The performance of a trained ML model generally improves with the increasing size of the training data. Therefore, here we assume that the certainty curve function $C(n)$ is an increasing function (however, not necessarily *strictly* increasing).

Once $C(n)$ is available for different values of n, or as a curve, one can use this to "solve" for the value of n, given a specific value of $C(n)$. For instance, based on analogy with statistical power, we can obtain the sample size that gives 80% certainty by empirically solving $n^* = C^{-1}(0.8)$. Since the certainty curve may not have a unique mapping between an input and an output value, we define the pseudo-inverse of $C(n)$ to be $C^{-1}(c)$: $= \min\{n: C(n) = c\}$, i.e., for a certainty value c (e.g., 0.8), $C^{-1}(c)$ is equal to the smallest n that gives $C(n) = c$.

The concept of certainty curves is inspired by the traditional statistical concept of power, used in hypothesis testing. Power is defined as the probability of "detecting a signal" (often expressed by the rejection of the null hypothesis using a statistical test), given that the signal exists, and it has a specific magnitude (expressed by the assumption that an alternative hypothesis is true). The analogy of our method with the definition of statistical power is as follows: the assumption "$ROC\text{-}AUC(P) \geq t_p$" plays the role of the alternative hypothesis being true, while the event "$ROC\text{-}AUC(S) \geq t_s$" plays the role of the detection of the "signal" of interest in a sample, in our case a model trained on a dataset of size n with sufficient performance ($ROC\text{-}AUC(S) \geq t_s$).

While our framework is described under the general case of independent values for t_p and t_s thresholds, in our evaluation, we adopt the approach where t_s is given as a fraction of t_p, i.e., $t_s = \lambda \cdot t_p$. Under that setting, the required sample size refers to the capacity of a specific modeling method to be used for training a model that achieves satisfactory performance *relevant to the maximum performance* (based on the population data). For example, using $\lambda = 0.85$, assuming that at a population level, the performance of ROC-AUC ≥ 0.8 (i.e., $t_p = 0.8$), the performance of a model developed using a sample of the data is deemed satisfactory if ROC-AUC $\geq t_s$, where $t_s = \lambda \cdot t_p = 0.85 \times 0.8 = 0.68$.

Calculating the "observed" sample size

For each of the K datasets ($K = 13$) used for the simulation, we can obtain the observed sample size n^* at a given level of certainty by first using dataset-specific univariable logistic regression models for modeling the certainty given in Equation 1, the outcome being whether the ROC-AUC values exceed the t_s threshold or not. Let $\text{logit}(C_{obs}(n)) = a + b\ln(n)$; then, a fitted model can be used for determining the sample size n^* required for obtaining a sufficient level of certainty C^*, for a given dataset, i.e., $n^* = \min\{n: C_{obs}(n) \geq C^*\}$. This is considered to be the true sample size required to achieve a certain certainty level. A logistic regression model is fitted on the 2,300 observations per dataset.

Using the logistic regression model allows us to smooth the relationship between the outcome and the sample size and ensure that it is monotonic. The a and b parameters are not expected to be the same across different datasets and are used to determine the n^* values used for performance evaluation.

A calculator of sample size

The sample size calculator uses a prediction model for estimating the certainty curve as a function of various dataset characteristics. Based on the certainty curve, the required sample size is obtained.

In our experiments, we have $P_k, k = 1, \ldots, K$ real datasets, n_i sample sizes, $S_{ijk} \subset P_k$ sample sets from the population data, where i refers to the sequence in the geometric series. The value of j pertains to the series, with $j = 1, \ldots, 100$. Then, for each value of i,j,k, we obtain a binary variable indicating whether the condition $ROC\text{-}AUC(S_{ijk}) \geq t_s$ is met based on our experiments. More specifically, we set

$$y_{ijk^*} = I\left(AUC\left(S_{ijk^*}\right) \geq t_s\right), j = 1, \ldots, 100, k^* \in \left\{k : AUC(P_k) \geq t_p\right\}. \quad \text{(Equation 2)}$$

Then, the tuple $\left\{y_{ijk^*}, \left|S_{ijk^*}\right|\right\}$ can be used for fitting a predictive calculator model.

Additional predictors in the calculator model relate to characteristics of the datasets that have previously been found to affect the sample size requirements. These are the amount of imbalance between the two classes of the binary outcome, the information provided by each predictor (measured by entropy), and the number of predictor parameters (or degrees of freedom [*df*]).[23,25] They are captured by the following variables used as predictors in the calculator.

- The imbalance factor (*IF*) is given by $IF = \max\{prev/(1 - prev), (1 - prev)/prev\}$, where *prev* is the prevalence of the outcome of interest. For example, if the outcome is mortality and 4% of the individuals in the dataset have died, then $prev = 0.04$ and $IF = 24$.

Table 2. Characteristics of the population datasets

Dataset	Imbalance factor	Mean standardized entropy	Degrees of freedom
COVID	66.59	0.79	47
CCHS	1.77	0.87	11
Nexoid	1.55	0.34	31
FAERS	9.06	0.62	14,834
TEXAS	1.47	0.67	345
WASHINGTON2007	1.04	0.69	11,000
BSA	1.34	0.91	400
WASHINGTON2008	2.93	0.65	11,127
CALIFORNIA2007	1.18	0.68	9,330
FLORIDA2007	1.53	0.69	45,145
NEWYORK2007	1.62	0.75	19,669
BORN	13.27	0.32	27
MIMIC-III	3.76	0.28	46

- The mean standardized entropy (*entr*) is given by the average value of standardized entropy across all predictors in the model. For any individual variable that has been discretized, it is defined as

$$-\frac{\sum_{i=1}^{z} p_i \log_2(p_i)}{\log_2(z)}, \quad \text{(Equation 3)}$$

where z is the total number of categories in that variable and p_i is the proportion of observations in category i. For continuous variables with fewer than 10 unique values, it is treated as categorical. If a continuous variable has more than 10 categories, then it is categorized using the Sturges method.

- *df* parameters are based on the number of predictors, where each predictor contributes a value of 1 if it is numerical or binary and a value of l if it is categorical with $(l + 1)$ levels, and summed across all predictors. For example, if a dataset had two variables, a binary one and a categorical one indicating the highest degree obtained, and there were six categories, then the value would be $df = 1 + 5 = 6$.

The values for the population datasets are summarized in Table 2. All the values across i, j, and k were used for fitting the calculator model as

$$y \sim n + \log(IF) + entr + \log(df). \quad \text{(Equation 4)}$$

The model in Equation 4 can then be used for estimating the probability values that define the certainty curve function $C(n)$ for any value of n. Each model had 29,900 observations for fitting.

These data are clustered, with the dataset constituting the clustering factor. While modeling methods for clustered data would be the natural choice, their benefits when prediction (and not explanation) is the modeling objective are questionable.[57] We therefore chose to use LGBM as the modeling method, given its often-found superior performance. Note that

this is a second and different use of LGBM to fit the sample size calculator.

Using this estimation method and the results from our simulation, we can generate different certainty curves according to Equation 4 for each of the three ML modeling methods used in our simulation and each of the λ values. A researcher can choose the right certainty curve for estimating the required sample size, given that a specific modeling method and λ value have been chosen *a priori*.

Assuming a model fitted for a specific ML method m and using a specific value for λ, for a future study assuming data with known values for characteristics *IF*, *entr*, and *df*, the estimated certainty for a given sample size n is denoted by $C_{\lambda,m}(IF, entr, df, n)$. Given that, the required sample size for obtaining a certainty value of at least C^* is given by $\hat{n} = \min\{n: C_{\lambda,m}(IF, entr, df, n) \geq C^*\}$.

The pseudocode for the sample size calculator is provided in the supplemental information.

Performance measure for sample size estimation models

We assess the performance of the certainty curve method by comparing the observed and predicted values of the required sample size for the K datasets, where predicted values, denoted by $\hat{n}$, were obtained using leave-one-dataset-out (i.e., the predicted sample size for a particular sample dataset was obtained by using a model trained on the remaining $K - 1$ dataset samples).

For each of the K datasets ($K = 13$) used for the simulation, the observed sample size n^* was obtained by first constructing the observed certainty curve $C_{obs}(n)$ and then selecting the sample size n^* that corresponds to the desired certainty value from the curve. This is considered to be the true sample size required to achieve the desired certainty level for a particular dataset.

Because we use leave-one-dataset-out, no observations used to fit the logistic regression model are used in training a sample size estimation model (see Equation 4) when that dataset is taken out. This ensures that no data leakage occurs.

The performance of a statistical estimation method,[26] as well as three other heuristic approaches, 10 events per variable,[23,24] 300 events per variable,[4] and 15 events per variable,[25] were also calculated as comparators. At the time of writing, these four methods are the only ones currently available for analysts to use for sample size estimation.

One metric that provides a measure of bias and the direction of bias is the median relative error (mRE), defined as

$$\text{mRE} = 100 \times \text{median}\left(\frac{\hat{n}_{k^*} - n^*_{k^*}}{n^*_{k^*}}\right). \qquad \text{(Equation 5)}$$

We also use the median of the absolute log quotient error (mALQE) between the observed and predicted required sample sizes. That is,

$$\text{mALQE} = 100 \times \left(\exp\left(\text{median}\left|\log\left(\frac{\hat{n}_{k^*}}{n^*_{k^*}}\right)\right|\right) - 1\right). \qquad \text{(Equation 6)}$$

It can be interpreted as the median symmetric absolute percentage deviation between the observed and predicted values. Values close to 0 indicate a better fit. This measure is consistent with measures discussed previously,[58–60] and it is based on the ratio between the observed and predicted sample sizes. It returns the same error when predicted and observed values are swapped. It is therefore symmetrical, and this is important so that under- and overestimation do not cancel each other out when aggregating across the whole dataset (as with mRE). By exponentiating the mALQE, we obtained a value of >1, playing the role of the ratio. By subtracting 1, we obtain a measure where 0 indicates a perfect fit. However, it does not give direction of bias, which the mRE provides.

RESULTS

The performance of the three ensemble models trained on the samples was plotted against the sample size for each dataset separately; large variability in the trend between sample size and ROC-AUC values across different datasets and different modeling methods was observed. For some datasets, a small sample size was sufficient for the model to achieve performance comparable to the population level, while in some other cases, the latter was achieved with only very large sample sizes. Samples from the COVID and Better Outcomes Registry & Network (BORN) datasets seem to approach population-level performance "the fastest" (i.e., requiring the smallest sample sizes), while samples from the WASHINGTON2007 dataset require very large sizes in order to approach population-level performance. Figures 2, 3, and 4 illustrate these results for the three types of ensemble models, where the red horizontal line indicates population-level ROC-AUC and the green line indicates the "target" ROC-AUC, equal to 0.85*(population ROC-AUC).

The sample size calculator model was fitted to estimate certainty using different values for parameter λ (0.8, 0.85, and 0.9). The discrepancy between the predicted and observed required sample sizes at 80% and 90% certainty, measured by the mRE and mALQE, was compared between the proposed certainty curve method and the four other previously proposed methods. The results for the mRE are given in Tables 3 and 4 and for the mALQE in Tables 5 and 6 at 80% and 90% certainty, respectively. In addition to these summary error measures, the comparison of the predicted and observed required sample sizes for the 13 datasets is depicted in the scatterplots in Figures S1 and S2.

We can see that the certainty curve approach underestimates the sample size for low λ and overestimates for larger values of λ. The other methods consistently overestimate the sample size, often by a very wide margin. It can also be observed that the most accurate sample size estimation is obtained when the λ value is 0.85, and this is consistent across all settings.

DISCUSSION

Summary

Having a sample size calculator for training ML models allows an analyst to deliberately balance the level of prognostic accuracy against the required sample size. To achieve prognostic accuracy that is closer to the optimal value (obtained with a very large dataset), the larger the sample size needs to be. However, there are no validated sample size calculators that can be used by data analysts, in general and specifically for health datasets, when

CellPress
OPEN ACCESS

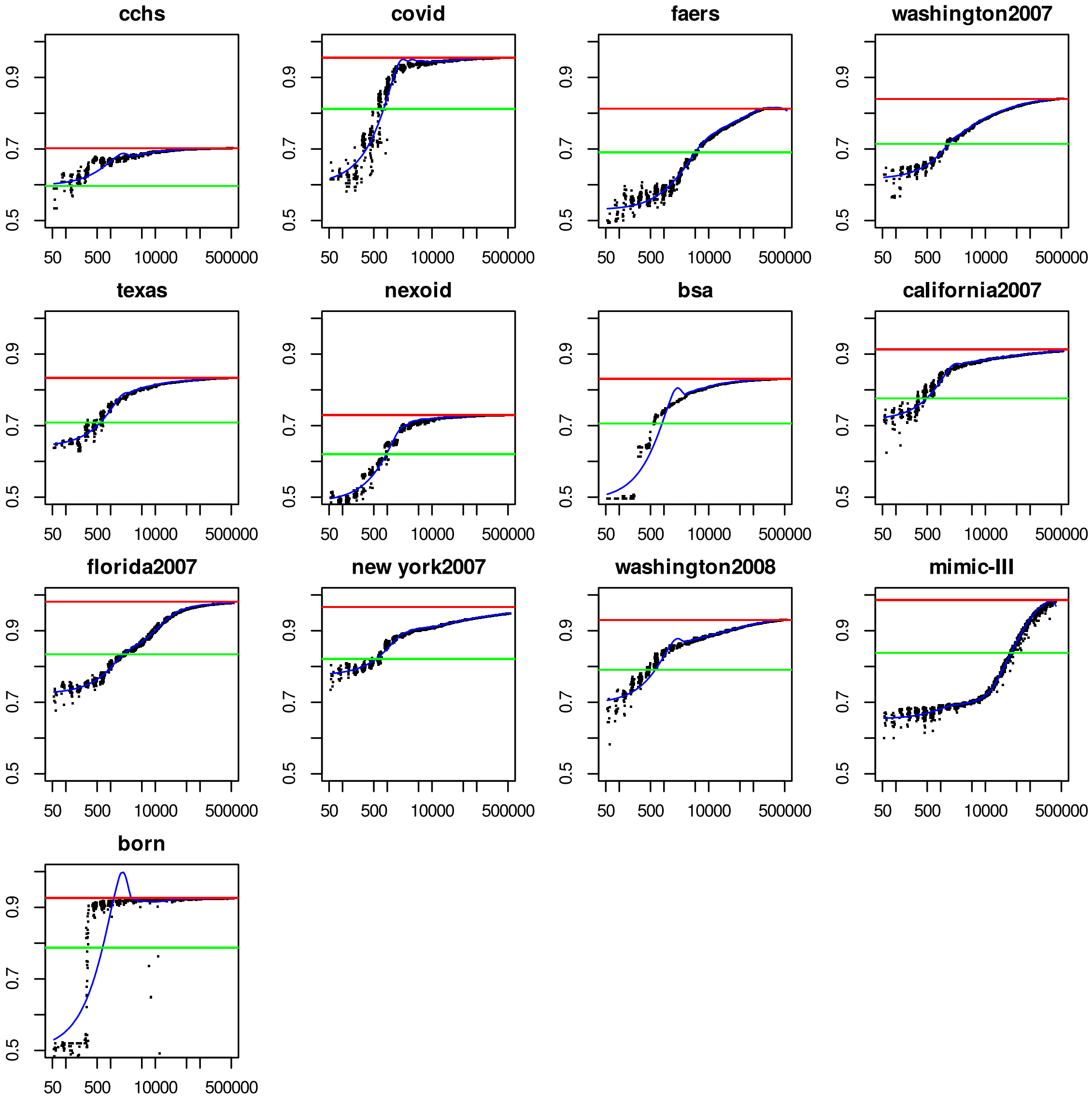

Figure 2. ROC-AUC values against sample size for LGBM models
The red (top horizontal) line indicates population ROC-AUC and the green (bottom horizontal) line indicates 0.85*(population ROC-AUC).

training ML models. In practice, most clinical predictive ML modeling studies do not provide an explanation for how the sample size was calculated.[3] This means that analysts need to rely on heuristics or sample size estimation methods that were not designed for ML applications. Oftentimes, researchers rely on sample sizes used in precedents, which tend to also be informed by the same heuristics, or use unsuitable calculators.[27]

In this study, we address this problem by developing an empirically based sample size calculator for tabular health data. The methodology we employ introduces the concept of a certainty curve that is parallel to traditional power analysis concepts. Three calculators were developed for ensemble methods, namely random forests and two boosted tree methods (LGBM and XGBoost). The R code for the calculators is available online.[61]

The analyst needs to determine the certainty level that they wish to have (analogous to the power level) to obtain a prognostic accuracy that is a λ proportion of the optimal value (analogous to the effect size). The analyst also needs to provide the various parameters that characterize the dataset, which capture the data complexity. These can be computed using domain knowledge, a small data sample, and/or data from previous studies with similar datasets.

For estimating the sample size, we trained an LGBM model using characteristics of the population level (*entr*, number of *df*, and class imbalance) as predictors. An alternative approach that seems attractive would be to instead use as predictors these characteristics but for the samples (which would be different from the parameter values at the population level). This approach would seemingly increase the accuracy of the calculator.

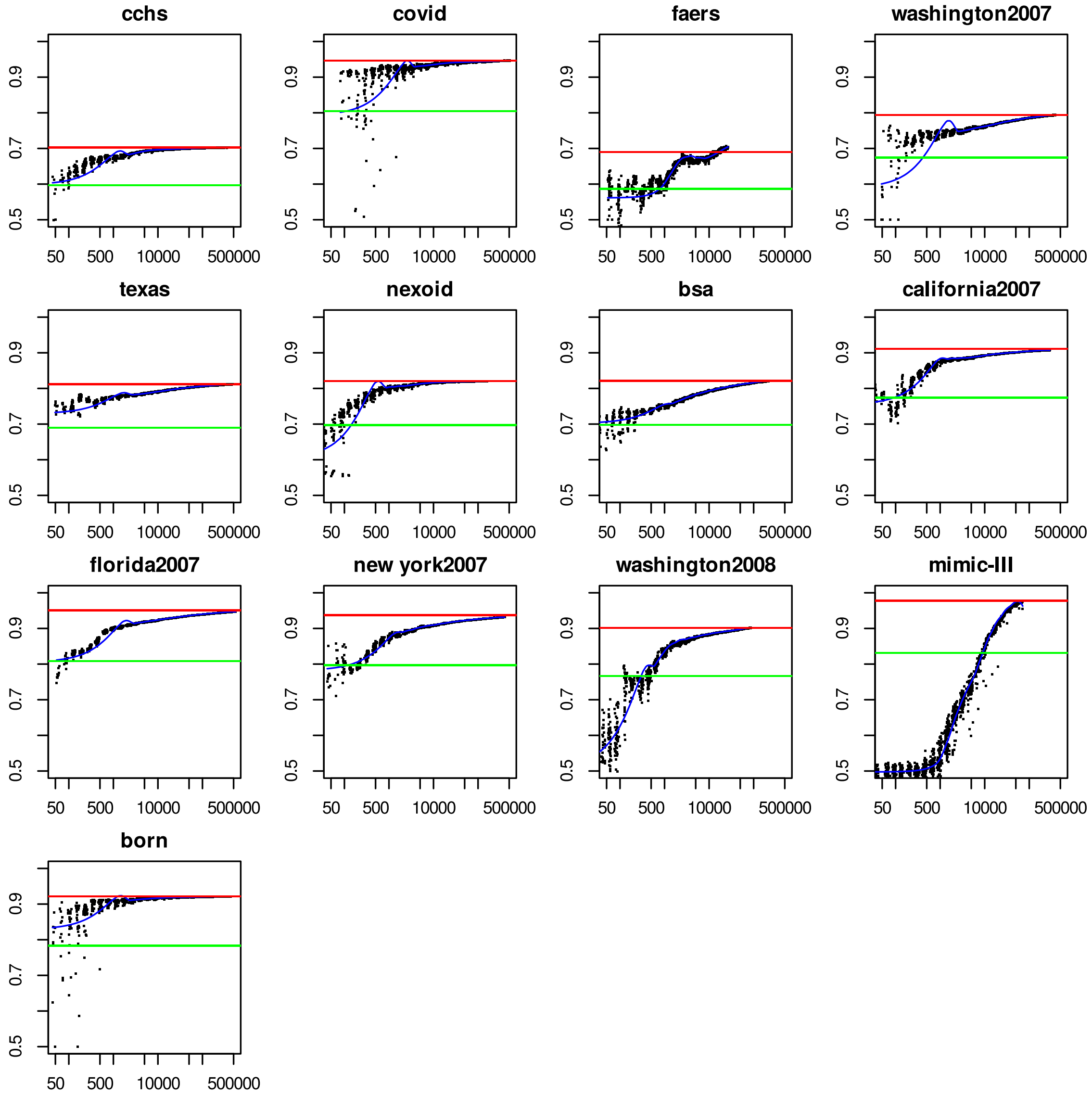

Figure 3. ROC-AUC values against sample size for RF models

The red (top horizontal) line indicates population ROC-AUC and the green (bottom horizontal) line indicates 0.85*(population ROC-AUC). RF, random forest.

However, such a model will not be of practical use for the estimation of the required sample size for achieving a specific performance target with the required certainty level. This is because in that case, the required sample size would be given by $\min\{n: C_{\lambda,m}(IF(n), entr(n), df(n), n) \geq C^*\}$, and the user would need to provide as input to the sample size calculator the hypothetical values for *IF*, *entr*, and *df* parameters that correspond to a nominal range of possible samples sizes, i.e., as functions of *n* (*IF*(*n*), *entr*(*n*), *df*(*n*)). These functions are largely heterogeneous across different datasets and therefore difficult to determine *a priori* by the user. For all of these reasons, we refrained from using the sample-specific values of the parameters as predictors in the LGBM model for the prediction of the certainty curve, using instead the population-level values, which would be more stable.

Other measures of dataset complexity have been proposed in the literature.[62] However, these would be difficult to compute without access to complete datasets, which is not going to be the case when estimating sample size during the study design phase of a project.

The results show that our calculator has an accuracy that is significantly better than existing heuristic methods or calculators designed for statistical methods. In fact, our results provide a compelling case against using the heuristics and the sample size calculators designed for regression models, as these tend to significantly overestimate the required sample size.

As shown in Figures S1 and S2, our sample size calculator has the largest error on the BORN and Medical Information Mart for Intensive Care (MIMIC) datasets. It is likely that there are other data characteristics that are not captured by our three model parameters and that this results in the full complexity of these datasets not being captured.

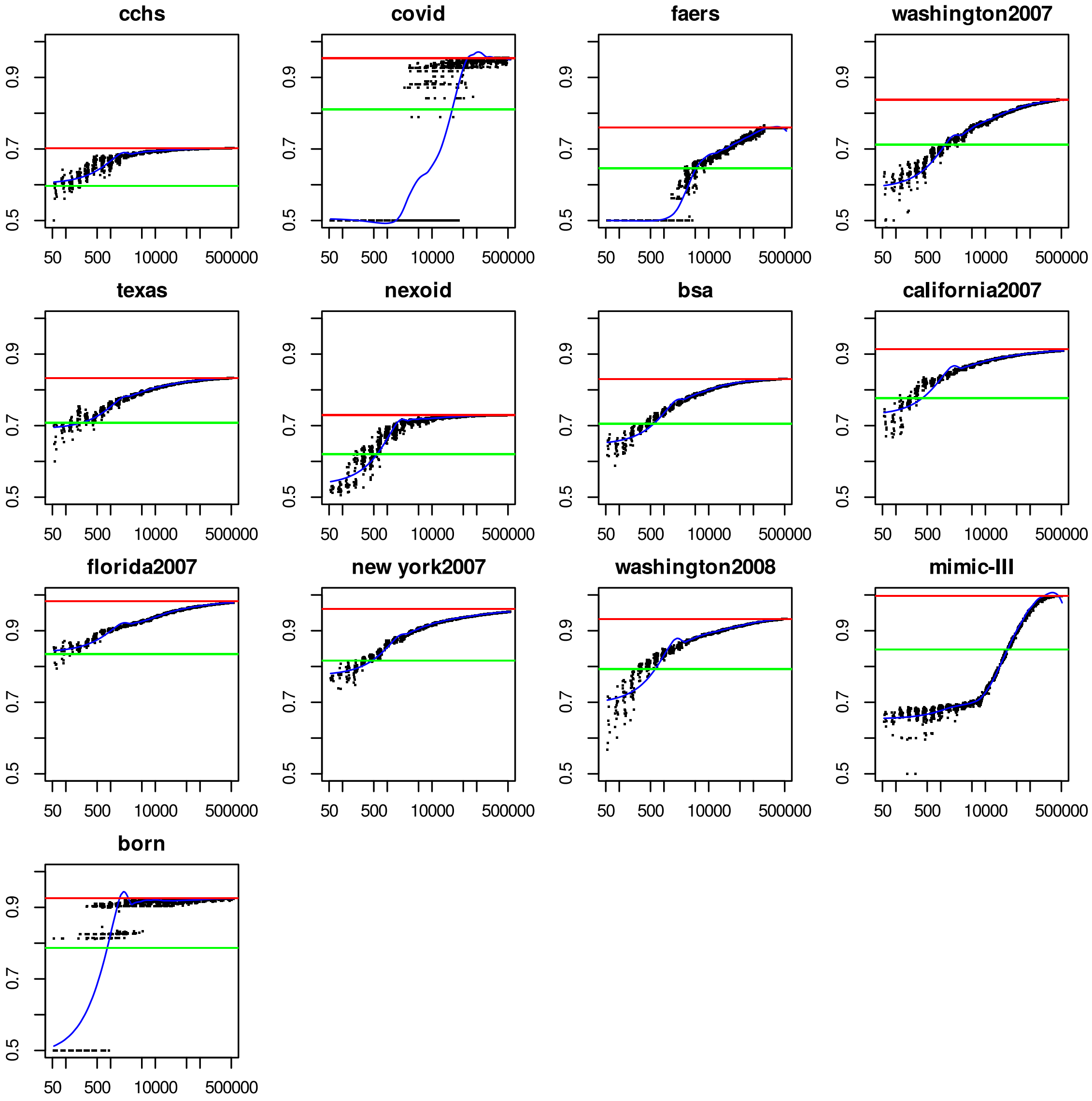

Figure 4. ROC-AUC values against sample size for XGB models

The red (top horizontal) line indicates population ROC-AUC and the green (bottom horizontal) line indicates 0.85*(population ROC-AUC). XGB, XGBoost.

The most accurate results using our sample size calculator are obtained at a λ of 0.85, which means that the model performance is at 85% of the optimal model performance with the full population. One explanation for that is that the slope of the relationship between the sample size and ROC-AUC is steepest at that λ value across all datasets, making it easier to get more precise sample size estimates. For the 0.8 and 0.9 λ values, one can adjust the estimated sample sizes by considering the mRE. For example, if at a λ of 0.9, the estimated sample size is 1,000 observations, then it can be adjusted down by 56%–641% for training an LGBM model.

The alternative and currently more often used approach to calculating the sample size for ML models is the Riley et al. method, originally developed for regression models. To illustrate the differences in error, we present some examples of sample size calculations using the certainty curve method, as well as the Riley et al. regression method. For our examples, we assume an LGBM model, λ = 0.85, and 80% certainty. For the first example, we use the COVID dataset, which has 47 *df* but a large *IF* of 66.59. The true required sample size is 670, while the one calculated using the certainty curve method is 450. The Riley et al. method, which is highly influenced by the large *IF*, predicts *n* = 19,168, which is an extreme overestimation.

In a different scenario, the WASHINGTON2007 dataset has almost perfect outcome balance (*IF* = 1.04) but a very large number of *df* (11,000). The true sample size is 1,410, and our method predicts *n* = 444—an underestimation. The Riley et al. method predicts *n* = 662,957, which is again an extreme overestimation.

Finally, for the Canadian Community Health Survey (CCHS) dataset, with 11 *df* and *IF* = 1.77, the true required sample size

Table 3. Comparison of the percentage median (IQR) relative error using leave-one-dataset-out at 80% certainty between the certainty curve approach and other methods (Riley et al., 300 events per variable, 15 events per variable, and 10 events per variable)

Model	λ	Certainty curve (%)	Riley (%)	EPV300 (%)	EPV15 (%)	EPV10 (%)
LGBM	0.80	−66.8 (−88.7, 79.1)	9,012 (4,219, 95,297.5)	142,094.5 (29,570.5, 1,448,657.9)	7,009.9 (1,383.6, 72,338.1)	4,639.9 (889.2, 48,192.1)
LGBM	0.85	16.5 (−62.9, 222.7)	4,270.3 (2,760.9, 57,274.4)	142,094.5 (29,570.5, 1,448,657.9)	7,009.9 (1,383.6, 72,338.1)	4,639.9 (889.2, 48,192.1)
LGBM	0.90	56.2 (−41.2, 1,131)	2,868.2 (1,843.9, 45,661.1)	142,094.5 (29,570.5, 1,448,657.9)	7,009.9 (1,383.6, 72,338.1)	4,639.9 (889.2, 48,192.1)
RF	0.80	−37.4 (−62.5, 23.6)	93,404 (12,797.1, 503,340.6)	732,748.5 (231,330, 4,680,502.3)	36,543.1 (11,472, 233,930.8)	24,328.5 (7,616, 155,920.8)
RF	0.85	8.8 (−25.3, 150.1)	57,824 (6,276, 345,942.8)	732,748.5 (231,330, 4,680,502.3)	36,543.1 (11,472, 233,930.8)	24,328.5 (7,616, 155,920.8)
RF	0.90	130.8 (31.9, 392.5)	38,930 (4,334, 247,243.9)	732,748.5 (231,330, 4,680,502.3)	36,543.1 (11,472, 233,930.8)	24,328.5 (7,616, 155,920.8)
XGB	0.80	−33 (−88.9, 47.3)	19,078.9 (1,339.6, 195,964.9)	134,669.5 (8,221.8, 2,253,523.3)	6,638.9 (316.4, 112,581.5)	4,392.6 (178.2, 7,5021.1)
XGB	0.85	56.7 (−80.7, 312.8)	11,970.5 (590.8, 121,139.6)	134,669.5 (8,221.8, 2,253,523.3)	6,638.9 (316.4, 112,581.5)	4,392.6 (178.2, 7,5021.1)
XGB	0.90	395 (−44, 1070.2)	8,108.9 (368, 86,185.9)	134,669.5 (8,221.8, 2,253,523.3)	6,638.9 (316.4, 112,581.5)	4,392.6 (178.2, 7,5021.1)

is $n = 100$, and our method predicts $n = 855$, while the Riley et al. method predicts $n = 3,667$.

Therefore, in all examples with different dataset characteristics, the Riley et al. method overestimates the sample size required. In a recent study, this method was assumed to be a lower bound for estimating the sample sizes required for ML prognostic models,[21] concluding that most clinical prediction studies using ML have sample sizes that are too small. Our results suggest that this regression-based approach for determining sample size is actually overestimating sample size requirements and therefore should not be used to determine minimal sample size requirements.

A recent study examined the impact of sample size on the prognostic performance of multiple types of ML models for a single digital mental health dataset.[5] Based on that, they recommended a minimal sample size of 750 for simple models and 1,000–1,500 observations for more complex models. These results provide practical guidance within a single domain, and it is uncertain if they can be applied to datasets of varying complexity and in different domains.

Another study developed models to predict sample sizes for untuned ML models and was trained on a mixture of real and synthetic datasets under an assumption of $\lambda = 0.97$,[63] with the sample size estimates shown in Table 7. These highlight an important point: setting a high λ can result in unrealistic sample sizes for contemporary clinical research. For example, if an estimator recommends 10 million observations for training an ML model with a very high λ, then that is not a useful result because it is unlikely that researchers can collect datasets of that size. Furthermore, some of these numbers are larger than the population size. Part of the high sample size estimate values may be attributed to ML models that are not tuned. It is also very likely that the high λ values in these estimation models result in a large overestimation of sample size. The λ values we used give more realistic sample size estimates and make explicit the performance expectations.

It is clear from our results that the certainty curve method is an improvement over existing heuristic and model-based methods, sometimes by multiple orders of magnitude. Nevertheless, the certainty curve method still exhibits wide variation in its accuracy

Table 4. Comparison of the percentage median relative error using leave-one-dataset-out at 90% certainty between the certainty curve approach and other methods (Riley et al., 300 events per variable, 15 events per variable, and 10 events per variable)

Model	λ	Certainty curve (%)	Riley (%)	EPV300 (%)	EPV15 (%)	EPV10 (%)
LGBM	0.80	−61.3 (−87.2, 149.2)	9,012 (4,219, 94,946)	142,094 (28,828, 1,448,657)	7,009 (1,346, 72,338)	4,639 (864, 48,192)
LGBM	0.85	25.3 (−60.6, 254.6)	4,270.3 (2,760.9, 57,063)	142,094 (28,828, 1,448,657)	7,009 (1,346, 72,338)	4,639 (864, 48,192)
LGBM	0.90	64.8 (−38, 1,165.2)	2,868.2 (1,843.9, 45,661)	142,094 (28,828, 1,448,657)	7,009 (1,346, 72,338)	4,639 (864, 48,192)
RF	0.80	−28.1 (−59.7, 22.7)	91,072 (12,797, 476,843)	680,402 (231,330, 4,346,173)	33,925 (11,472, 217,214)	22,583 (7,616, 144,776)
RF	0.85	9.3 (−21.1, 166.8)	55,466 (6,276, 327,730)	680,402 (231,330, 4,346,173)	33,925 (11,472, 217,214)	22,583 (7,616, 144,776)
RF	0.90	137.4 (62.2, 444)	36,958 (4,334, 234,225)	680,402 (231,330, 4,346,173)	33,925 (11,472, 217,214)	22,583 (7,616, 144,776)
XGB	0.80	−22.7 (−86.8, 72.3)	19,078.9 (1,311, 193,711)	134,669 (7,528, 2,253,523)	6,638 (281, 112,581)	4,392 (155, 75,021)
XGB	0.85	74.6 (−77.5, 368.6)	11,970.5 (577, 121,139)	134,669 (7,528, 2,253,523)	6,638 (281, 112,581)	4,392 (155, 75,021)
XGB	0.90	471.2 (−34.8, 1136.1)	8,108.9 (358, 86,185)	134,669 (7,528, 2,253,523)	6,638 (281, 112,581)	4,392 (155, 75,021)

Table 5. Comparison of the percentage median absolute quotient error using leave-one-dataset-out at 80% certainty between the certainty curve approach and other methods (Riley et al., 300 events per variable, 15 events per variable, and 10 events per variable)

Model	λ	Certainty Curve	Riley	EPV300	EPV15	EPV10
LGBM	0.80	482	9,012	142,095	7,010	4,640
LGBM	0.85	222	4,270	142,095	7,010	4,640
LGBM	0.90	345	3,227	142,095	7,010	4,640
RF	0.80	121	93,404	732,749	36,543	24,328
RF	0.85	55	57,824	732,749	36,543	24,328
RF	0.90	202	38,930	732,749	36,543	24,328
XGB	0.80	681	1,9079	134,670	6,639	4,393
XGB	0.85	418	11,971	134,670	6,639	4,393
XGB	0.90	397	8,109	134,670	6,639	4,393

Table 6. Comparison of the percentage median absolute quotient error using leave-one-dataset-out at 90% certainty between the certainty curve approach and other methods (Riley et al., 300 events per variable, 15 events per variable, and 10 events per variable)

Model	λ	Certainty curve	Riley	EPV300	EPV15	EPV10
LGBM	0.80	465	9,012	142,095	7,010	4,640
LGBM	0.85	239	4,270	142,095	7,010	4,640
LGBM	0.90	390	3,239	142,095	7,010	4,640
RF	0.80	109	91,072	680,402	33,926	22,584
RF	0.85	45	55,466	680,402	33,926	22,584
RF	0.90	203	36,958	680,402	33,926	22,584
XGB	0.80	549	19,079	134,670	6,639	4,393
XGB	0.85	369	11,971	134,670	6,639	4,393
XGB	0.90	489	8,109	134,670	6,639	4,393

even when the mRE is quite small, and it can benefit from further work to improve the sample size calculation model.

We have provided an R package that can be used to compute sample size requirements for future studies using these three ML models we investigated.[63] Future work can extend this study to other types of ML models.

These results and accompanying code can support organizations in their medical device submissions to health regulators that include ML models, whereby the regulators stipulate the need for the use of an adequate sample size.[64] Furthermore, recent reporting guidelines for ML research studies require the provision of sample size justifications.[65–67]

Impact of additional data complexity characteristics

Plots of discrepancies between observed and predicted sample sizes for all 13 datasets, shown in Figures S1 and S2, reveal a systematic discrepancy for the MIMIC-III (underprediction) and BORN (overprediction) datasets. This discrepancy appears to be present in most combinations of ML methods and λ values used. One possible explanation for this discrepancy is that the data characteristic variables used as predictors for the model to estimate the certainty curve and predict the required sample size are missing important data characteristics that impact the sample size requirements.

While the data characteristics we considered in the sample size calculator reflect those that can be calculated with reasonable certainty before data collection (during study design) or at early stages of data collection, there may be data complexity characteristics that are influential that are not easily computed. We nevertheless explore such complexity metrics to understand why MIMIC-III and BORN behave differently.

We explored the relevance of additional data complexity metrics, namely L2 and mutual information (MI). The L2 metric has been used to represent data complexity,[62,68] and it indicates linear separability (returning the misclassification error of a linear support vector machine (SVM) model on the entire dataset), with high values indicating less linear separability. The MI metric is calculated as the average pairwise MI values across all pairs of predictors in the dataset. High MI indicates that the predictors are dependent on each other, and therefore, there is greater redundancy among them.

We calculated values for these complexity metrics for large-sized subsets (n = 10,000, 20,000, 30,000, 40,000, and 50,000) from the original data. Given the computationally intensive nature of these metrics, we used linear regression models of the metric values against the sample size and projected the values for datasets of size n = 100,000. Patterns observed for this sample size would be relevant and present in the full datasets as well. The projected values are shown in Table 8.

For the BORN dataset, we see that it has the lowest value for L2, indicating a very high linear separability, suggesting that a simple prediction model can achieve high prediction accuracy. Such model simplicity would also explain why a smaller sample size would be sufficient for good performance. This is evident in the plots in Figures 2, 3, and 4. For this type of dataset, the sample size estimation model overestimated the sample size needed to reach high accuracy.

We can see in Figures 2, 3, and 4 that for the MIMIC-III dataset, the benefit of larger sample sizes only occurs at relatively large numbers, with a distinct "elbow" at around N = 10,000, which is quite different from the other datasets. Some of this behavior may be explained by the relatively low MI value, suggesting limited redundancy among the predictors, as well as middling L2 separability. Therefore, unlike the BORN dataset, the non-redundant predictors have lower predictive power, requiring a more complex prediction model, and thus more data, to improve performance. Because of the relatively favorable values of other variables (*IF* and *df*), the predicted sample size is not very high; however, the observed values are much higher.

Therefore, additional complexity metrics, which would be difficult to collect during the early stages of a study before data collection, can potentially improve the sample size prediction.

Sensitivity analysis

There are two types of sensitivity analyses that are relevant to the sample size estimation model. The first pertains to sensitivity to a particular dataset that is used in its training. The second is sensitivity to the accuracy of the input values that characterize a dataset.

Table 7. Sample size estimates for our original 13 datasets using the estimation models in REF.[63] for random forest and XGBoost models

Dataset	RF	XGB
BORN	89,961	202,410
BSA	6,004	59,595
CALIFORNIA2007	4.8E+13	1,696,640,204
CCHS	6,714,146	1,409,297
COVID	186,712	281,171
FAERS	14,805,033,363	45,100,940
FLORIDA2007	3E+15	10,920,225,797
MIMIC-III	1.59E+52	3.55E+26
Nexoid	N/A[a]	N/A[a]
NEWYORK2007	5.96E+18	3.29E+11
TEXAS	209,326	294,405
WASHINGTON2007	1.47E+25	2.47E+14
WASHINGTON2008	1.50E+22	1.12E+13

The original paper did not have results for LGBM.

[a]One of the parameters required for using this estimator is the difference in performance between logistic regression and the ML model, and that is used as a measure of non-linearity. The estimator expects the ML model to always perform better. For the Nexoid dataset, logistic regression performed better than XGB, and therefore, this resulted in model misbehavior/failure.

Table 8. The projected values for complexity metrics for the 13 datasets

Dataset	MI	L2
BORN	0.002	0.054
BSA	0.241	0.273
CALIFORNIA2007	0.191	0.189
CCHS	0.069	0.364
COVID	0.082	0.101
FAERS	0.708	0.070
FLORIDA2007	0.129	0.182
MIMIC-III	0.028	0.115
Nexoid	0.002	0.196
NEWYORK2007	0.299	0.172
TEXAS	0.05	0.259
WASHINGTON2007	0.376	0.280
WASHINGTON2008	0.182	0.213

With respect to sensitivity to the training dataset, Tables S15–S18 present the mALQE and mRE values after the removal of each training dataset. The mRE results show greater variability than the mALQE results, demonstrating higher sensitivity to individual datasets. For the mRE results, datasets such as COVID, FDA Adverse Event Reporting System (FAERS), WASHINGTON2007, and TEXAS had a sizable impact on the results when they were removed, indicating sensitivity to that performance metric. For example, the mRE for 80% certainty and 0.85 λ on the LGBM model changed from 16% to 77.56% for these datasets and from 56.2% to 83.42% for the 0.9 λ value. The mALQE results were less impacted by the datasets, demonstrating more robustness to the training data used for that metric. Notwithstanding the sensitivity to these specific training datasets, these results would not change the overall findings from our study.

The user of our sample size estimation model would need to provide three inputs: *entr*, number of *df*, and class imbalance. These values would be needed at an early stage of a study to determine how much data to collect.

The number of *df* (the number of categories for categorical variables) would be known *a priori*, as these would be defined as part of the study design (e.g., response categories in a questionnaire or diagnoses in a set of inclusion criteria). Plus, this information would be available from previous ML modeling work that used similar datasets in the domain, as it is almost always reported as part of descriptive statistics, and ML modeling reporting guidelines require that information to be reported (for example, see Klement and El Emam,[65] El Emam et al.,[66] and Collins et al.[67]).

Similarly, the class imbalance would be known from previous studies, and an analyst's domain knowledge would indicate whether an outcome is rare or common. Such imbalance information is required as part of ML modeling reporting guidelines.[65,67]

However, the *entr* requires an approximate knowledge of the distribution of the variables. Instead, one needs to rely on previous studies to estimate it or data collected in a smaller feasibility study, which may lead to imprecision in its calculation. To examine this, we performed a post hoc sensitivity analysis to determine how much the predicted sample size using our calculator is affected by inaccuracies in the calculation of the *entr*.

We used 17 medium-sized datasets with binary outcomes[69–85] (a summary of these datasets is provided in Table S19) and calculated the estimates for *entr*, number of *df*, and *IF* based on the data. Using these values and our sample size calculator, we estimated the required sample size based on $\lambda = 0.85$ and a certainty of 0.8. Subsequently, we recalculated the predicted sample size but this time used values for standardized mean entropy that deviate from the original value by some amount. We measure the discrepancy in the sample size prediction as the percentage of relative error given by $100*(n' - n)/n$, where n and n' denote the calculated sample size when the current and deviated entropy value is used, respectively. Subsequently, we calculated an aggregate measure of discrepancy as the proportion of the datasets where the relative error does not exceed a prespecified value (10%, 25%, and 50%).

Table 9 presents the aggregate discrepancy for different values of standardized entropy deviation. For example, for a deviation of -0.25 in the *entr*, 47% of the sample size estimates were within 10% of the value estimates with the correct *entr*. This means that approximately half the time, the sample size is within 10% of the actual estimated value if there was no error in calculating the *entr* value.

The table shows that medium-level deviation results in modest discrepancy for the sample size estimate. When the deviation of *entr* approaches ±0.5, the sample size estimates become less reliable. Therefore, the sample size estimator is robust to small to medium errors in the calculation of this parameter. However, analysts should still refer to prior studies to get the best approximation of the distribution of predictor variables in a dataset.

Table 9. Aggregate discrepancy (as the percentage of relative difference from the originally predicted value) for the calculated sample size, for different levels of deviation of the mean standardized entropy

	Deviation in mean standardized entropy					
Discrepancy	−0.5	−0.25	−0.1	0.1	0.25	0.5
Median	48	2	0	−1	−1	−1
Within 10%	17.6	47	64.7	41.1	41.1	29.4
Within 25%	23.5	52.9	82.3	70.5	76.4	70.5
Within 50%	58.8	82.35	88.2	100	100	100

Detailed results of the discrepancy for each individual dataset are found in Table S20.

Future work

The error we observe in our estimators may be a function of the heterogeneity of the datasets that we used in our study. This was a deliberate decision to ensure that the results would be applicable across a broad set of health domains. However, to the extent that heterogeneity affected model error, future work can follow the provided methodology to develop new sample size estimators that are suitable for more homogeneous datasets. This can be achieved by having more domain-specific datasets (e.g., many public health datasets) and, for each domain, developing a different sample size estimator that is specific. The development of a catalog of such specific sample size estimators would work best if there were sharp definitions of the domains so that an analyst can easily determine which estimator is most applicable.

Analysis of additional data complexity metrics that cannot be computed at the start of a study, before data collection, indicates that they may capture important data characteristics that impact sample size computation. These were not included in our model because they are not useful in a practical setting where decisions need to be made during study design. However, they do suggest further data complexity metrics that future work should attempt to estimate.

The concepts and methods presented in this study can be extended in future work to genetic data and imaging data to enable sample size calculations for these data modalities.

Limitations

Our sample size calculator was developed using 13 large datasets. These datasets covered different situations, including public health, hospital discharge, ICUs, registry, adverse events, and health surveys. This heterogeneity arguably improves the generalizability of the results. However, this heterogeneity also likely caused the sensitivity of our results to the training datasets used, as assessed using the mRE. A more homogeneous set of data would provide sample size calculators that are tailored to specific domains and that would more completely capture potential non-linearities that appear in practice between the features and outcomes.

The sample characteristics, such as the *entr*, may need to be estimated from similar datasets. However, our sensitivity analysis indicates that the sample size calculator is tolerant of medium levels of imprecision in the calculation of *entr*.

Ethics

This study was approved by the CHEO Research Institute Research Ethics Board protocol number 24/36X.

RESOURCE AVAILABILITY

Lead contact

Requests for further information and resources should be directed to and will be fulfilled by the lead contact, Khaled El Emam (kelemam@ehealthinformation.ca).

Materials availability

This study did not generate new unique reagents.

Data and code availability

The following provides information on the availability of each of the datasets.

- BORN[37]: BORN collects Ontario's prescribed perinatal, newborn, and child registry with the role of facilitating quality care for families across the province. It can be accessed through a data request at https://bornontario.ca/en/data/data.aspx.
- Basic Stand Alone (BSA)[36]: the BSA inpatient claims dataset is about claim-level information, where each record is an inpatient claim incurred by a 5% sample of Medicare beneficiaries. The dataset is publicly available at https://www.cms.gov/data-research/statistics-trends-and-reports/basic-stand-alone-medicare-claims-public-use-files/bsa-inpatient-claims-puf.
- California, Florida, New York, and Washington State Inpatient Databases (SID)[35] Healthcare Cost and Utilization Project (HCUP) and Agency for Healthcare Research and Quality: these datasets contain the patient's hospital discharge information for 2007 and 2008 and are available for purchase at https://hcup-us.ahrq.gov/tech_assist/centdist.jsp.
- CCHS[31]: CCHS data are Canadian population-level information concerning health status, health system utilization, and health determinants collected by Statistics Canada through a telephone survey. The availability of CCHS data is restricted and requires an access request at https://www150.statcan.gc.ca/n1/pub/82-620-m/2005001/4144189-eng.htm.
- COVID-19[30]: the COVID-19 dataset collects Canadian health records of COVID-19 gathered by the Public Health Agency of Canada and is available at Esri Canada (https://resources-covid19canada.hub.arcgis.com/).
- FAERS[33]: FAERS is a database comprising information on adverse event and medication error reports submitted to the FDA and can be downloaded at https://open.fda.gov/data/faers/.
- MIMIC-III[38]: MIMIC-III is a large database that contains deidentified health-related data associated with over 40,000 patients who stayed in critical care units of the Beth Israel Deaconess Medical Center between 2001 and 2012. Access to the MIMIC database is granted upon signing a data use agreement with PhysioNet at https://physionet.org/content/mimiciii/1.4/.
- COVID-19 Survival (Nexoid)[32]: the COVID-19 survival dataset comprises web-based survey data collected by a company called Nexoid in the UK. It is publicly available at https://www.covid19survivalcalculator.com/en/download.
- Texas Hospital Discharge[34]: the TEXAS dataset contains the patient's hospital discharge information from Texas and is available at (may require a fee) https://www.dshs.texas.gov/center-health-statistics/chs-data-sets-reports/texas-health-care-information-collection/health-data-researcher-information/texas-inpatient-public-use.

The code used in this analysis can be accessed as follows.

- The ML modeling was performed using the R sdgm package available from https://osf.io/DCJM6.[56]
- The R code for applying the sample size estimation models on a new dataset is available from https://osf.io/7bs8q/.[61]

ACKNOWLEDGMENTS

The authors thank Fida Dankar for reviewing an earlier version of this manuscript. This research is funded by the Canada Research Chairs Program through the Canadian Institutes of Health Research, Discovery Grant RGPIN-2022-04811 from the Natural Sciences and Engineering Research Council of Canada, and the Canadian Children Inflammatory Bowel Disease Network.

AUTHOR CONTRIBUTIONS

Conceptualization and design, N.M., K.E.E., and D.L.; analysis, simulations, and drafting of the initial version of the article, N.M., K.E.E., and D.L.; reviewing and revising the article, N.M., K.E.E., and D.L.; drafting the manuscript, N.M., K.E.E., and D.L.; review and editing, N.M., K.E.E., D.L., and T.W.

DECLARATION OF INTERESTS

At the time of writing, K.E.E. was the Scholar-in-Residence at the Office of the Information and Privacy Commissioner of Ontario.

SUPPLEMENTAL INFORMATION

Supplemental information can be found online at https://doi.org/10.1016/j.patter.2026.101498.

Received: December 24, 2024
Revised: May 14, 2025
Accepted: February 3, 2026
Published: March 26, 2026

REFERENCES

1. Szucs, D., and Ioannidis, J.P.A. (2017). Empirical assessment of published effect sizes and power in the recent cognitive neuroscience and psychology literature. PLoS Biol. *15*, e2000797. https://doi.org/10.1371/journal.pbio.2000797.
2. Riley, R.D., and Collins, G.S. (2023). Stability of clinical prediction models developed using statistical or machine learning methods. Biom. J. *65*, 2200302. https://doi.org/10.1002/bimj.202200302.
3. Riley, R.D., Ensor, J., Snell, K.I.E., Archer, L., Whittle, R., Dhiman, P., Alderman, J., Liu, X., Kirton, L., Manson-Whitton, J., et al. (2025). Importance of sample size on the quality and utility of AI-based prediction models for healthcare. Lancet Digit. Health *7*, 100857. https://doi.org/10.1016/j.landig.2025.01.013.
4. van der Ploeg, T., Austin, P.C., and Steyerberg, E.W. (2014). Modern modelling techniques are data hungry: a simulation study for predicting dichotomous endpoints. BMC Med. Res. Methodol. *14*, 137. https://doi.org/10.1186/1471-2288-14-137.
5. Zantvoort, K., Nacke, B., Görlich, D., Hornstein, S., Jacobi, C., and Funk, B. (2024). Estimation of minimal data sets sizes for machine learning predictions in digital mental health interventions. npj Digit. Med. *7*, 1–10. https://doi.org/10.1038/s41746-024-01360-w.
6. Halpern, S.D., Karlawish, J.H.T., and Berlin, J.A. (2002). The Continuing Unethical Conduct of Underpowered Clinical Trials. JAMA *288*, 358–362. https://doi.org/10.1001/jama.288.3.358.
7. Cohen, J. (1988). Statistical Power Analysis for the Behavioral Sciences (L. Erlbaum Associates).
8. Koppe, G., Meyer-Lindenberg, A., and Durstewitz, D. (2021). Deep learning for small and big data in psychiatry. Neuropsychopharmacol *46*, 176–190. https://doi.org/10.1038/s41386-020-0767-z.
9. Andaur Navarro, C.L., Damen, J.A.A., van Smeden, M., Takada, T., Nijman, S.W.J., Dhiman, P., Ma, J., Collins, G.S., Bajpai, R., Riley, R.D., et al. (2023). Systematic review identifies the design and methodological conduct of studies on machine learning-based prediction models. J. Clin. Epidemiol. *154*, 8–22. https://doi.org/10.1016/j.jclinepi.2022.11.015.
10. Luedtke, A., Sadikova, E., and Kessler, R.C. (2019). Sample Size Requirements for Multivariate Models to Predict Between-Patient Differences in Best Treatments of Major Depressive Disorder. Clin. Psychol. Sci. *7*, 445–461. https://doi.org/10.1177/2167702618815466.
11. Figueroa, R.L., Zeng-Treitler, Q., Kandula, S., and Ngo, L.H. (2012). Predicting sample size required for classification performance. BMC Med. Inform. Decis. Mak. *12*, 8. https://doi.org/10.1186/1472-6947-12-8.
12. Mukherjee, S., Tamayo, P., Rogers, S., Rifkin, R., Engle, A., Campbell, C., Golub, T.R., and Mesirov, J.P. (2003). Estimating Dataset Size Requirements for Classifying DNA Microarray Data. J. Comput. Biol. *10*, 119–142. https://doi.org/10.1089/106652703321825928.
13. Raudys, S.J., and Jain, A.K. (1990). Small sample size effects in statistical pattern recognition: recommendations for practitioners and open problems. In [1990] Proceedings. 10th International Conference on Pattern Recognition (IEEE Comput. Soc. Press), pp. 417–423. https://doi.org/10.1109/ICPR.1990.118138.
14. Larracy, R., Phinyomark, A., and Scheme, E. (2021). Machine Learning Model Validation for Early Stage Studies with Small Sample Sizes. In 2021 43rd Annual International Conference of the IEEE Engineering in Medicine & Biology Society (EMBC) (IEEE), pp. 2314–2319. https://doi.org/10.1109/EMBC46164.2021.9629697.
15. Vabalas, A., Gowen, E., Poliakoff, E., and Casson, A.J. (2019). Machine learning algorithm validation with a limited sample size. PLoS One *14*, e0224365. https://doi.org/10.1371/journal.pone.0224365.
16. Fukunaga, K., and Hayes, R.R. (1989). Effects of sample size in classifier design. IEEE Trans. Pattern Anal. Machine Intell. *11*, 873–885. https://doi.org/10.1109/34.31448.
17. D'souza, R.N., Huang, P.-Y., and Yeh, F.-C. (2020). Structural Analysis and Optimization of Convolutional Neural Networks with a Small Sample Size. Sci. Rep. *10*, 834. https://doi.org/10.1038/s41598-020-57866-2.
18. Balki, I., Amirabadi, A., Levman, J., Martel, A.L., Emersic, Z., Meden, B., Garcia-Pedrero, A., Ramirez, S.C., Kong, D., Moody, A.R., and Tyrrell, P.N. (2019). Sample-Size Determination Methodologies for Machine Learning in Medical Imaging Research: A Systematic Review. Can. Assoc. Radiol. J. *70*, 344–353. https://doi.org/10.1016/j.carj.2019.06.002.
19. Beleites, C., Neugebauer, U., Bocklitz, T., Krafft, C., and Popp, J. (2013). Sample size planning for classification models. Anal. Chim. Acta *760*, 25–33. https://doi.org/10.1016/j.aca.2012.11.007.
20. Acharjee, A., Larkman, J., Xu, Y., Cardoso, V.R., and Gkoutos, G.V. (2020). A random forest based biomarker discovery and power analysis framework for diagnostics research. BMC Med. Genomics *13*, 178. https://doi.org/10.1186/s12920-020-00826-6.
21. Tsegaye, B., Snell, K.I.E., Archer, L., Kirtley, S., Riley, R., Sperrin, M., van Calster, B., Collins, G., and Dhiman, P. (2024). Larger Sample Sizes are Needed When Developing a Clinical Prediction Model Using Machine Learning in Oncology: Methodological Systematic Review. Preprint at (Social Science Research Network). https://doi.org/10.2139/ssrn.4816525.
22. Dhiman, P., Ma, J., Andaur Navarro, C.L., Speich, B., Bullock, G., Damen, J.A.A., Hooft, L., Kirtley, S., Riley, R.D., Van Calster, B., et al. (2022). Methodological conduct of prognostic prediction models developed using machine learning in oncology: a systematic review. BMC Med. Res. Methodol. *22*, 101–116. https://doi.org/10.1186/s12874-022-01577-x.
23. van Smeden, M., Moons, K.G., de Groot, J.A., Collins, G.S., Altman, D.G., Eijkemans, M.J., and Reitsma, J.B. (2019). Sample size for binary logistic prediction models: Beyond events per variable criteria. Stat. Methods Med. Res. *28*, 2455–2474. https://doi.org/10.1177/0962280218784726.
24. Cai, Y.-Q., Gong, D.-X., Tang, L.-Y., Cai, Y., Li, H.-J., Jing, T.-C., Gong, M., Hu, W., Zhang, Z.-W., Zhang, X., and Zhang, G.W. (2024). Pitfalls in Developing Machine Learning Models for Predicting Cardiovascular Diseases: Challenge and Solutions. J. Med. Internet Res. *26*, e47645. https://doi.org/10.2196/47645.

CellPress
OPEN ACCESS

25. Harrell, F.E. (2015). Regression Modeling Strategies: With Applications to Linear Models, Logistic and Ordinal Regression, and Survival Analysis (Springer International Publishing). https://doi.org/10.1007/978-3-319-19425-7.
26. Riley, R.D., Ensor, J., Snell, K.I.E., Harrell, F.E., Martin, G.P., Reitsma, J.B., Moons, K.G.M., Collins, G., and van Smeden, M. (2020). Calculating the sample size required for developing a clinical prediction model. BMJ *368*, m441. https://doi.org/10.1136/bmj.m441.
27. Infante, G., Miceli, R., and Ambrogi, F. (2023). Sample size and predictive performance of machine learning methods with survival data: A simulation study. Stat. Med. *42*, 5657–5675. https://doi.org/10.1002/sim.9931.
28. Carriero, A., Luijken, K., de Hond, A., Moons, K.G.M., van Calster, B., and van Smeden, M. (2025). The Harms of Class Imbalance Corrections for Machine Learning Based Prediction Models: A Simulation Study. Stat. Med. *44*, e10320. https://doi.org/10.1002/sim.10320.
29. Goldenholz, D.M., Sun, H., Ganglberger, W., and Westover, M.B. (2023). Sample Size Analysis for Machine Learning Clinical Validation Studies. Biomedicines *11*, 685. https://doi.org/10.3390/biomedicines11030685.
30. Esri Canada (2023). Canadian health records of COVID-19 gathered by the Public Health Agency of Canada. https://resources-covid19canada.hub.arcgis.com/.
31. Canadian Community Health Survey (2021). Statistics Canada. https://www150.statcan.gc.ca/n1/pub/82-620-m/2005001/4144189-eng.htm.
32. Nexoid (2021). COVID-19 survival dataset. https://www.covid19survivalcalculator.com/en/download.
33. FDA Adverse Event Reporting System (2018). Database comprising information on adverse event and medication error reports submitted to FDA. https://open.fda.gov/data/faers/.
34. Texas Inpatient Public Use Data File (2025). Patient hospital discharge information from Texas hospitals. https://www.dshs.texas.gov/center-health-statistics/chs-data-sets-reports/texas-health-care-information-collection/health-data-researcher-information/texas-inpatient-public-use.
35. Healthcare Cost and Utilization Project (HCUP), Agency for Healthcare Research and Quality (2025). California, Florida, New York, and Washington State Inpatient Databases (SID). https://hcup-us.ahrq.gov/tech_assist/centdist.jsp.
36. Centers for Medicare & Medicaid Services (CMS) (2025). Basic Stand Alone (BSA) Inpatient Claims Public Use File (PUF). https://www.cms.gov/data-research/statistics-trends-and-reports/basic-stand-alone-medicare-claims-public-use-files/bsa-inpatient-claims-puf.
37. Better Outcomes Registry & Network (BORN) Ontario (2021). Data Resource Profile: Better Outcomes Registry & Network (BORN) Ontario. Int. J. Epidemiol. *50*, 1416–1425. https://doi.org/10.1093/ije/dyab033.
38. Johnson, A.E.W., Pollard, T.J., Shen, L., Lehman, L.W.H., Feng, M., Ghassemi, M., Moody, B., Szolovits, P., Celi, L.A., and Mark, R.G. (2016). MIMIC-III, a freely accessible critical care database. Sci. Data *3*, 160035. https://doi.org/10.1038/sdata.2016.35.
39. McNamara, M.E., Zisser, M., Beevers, C.G., and Shumake, J. (2022). Not just "big" data: Importance of sample size, measurement error, and uninformative predictors for developing prognostic models for digital interventions. Behav. Res. Ther. *153*, 104086. https://doi.org/10.1016/j.brat.2022.104086.
40. Guo, Y., Graber, A., McBurney, R.N., and Balasubramanian, R. (2010). Sample size and statistical power considerations in high-dimensionality data settings: a comparative study of classification algorithms. BMC Bioinf. *11*, 447. https://doi.org/10.1186/1471-2105-11-447.
41. Jacobucci, R., and Grimm, K.J. (2020). Machine Learning and Psychological Research: The Unexplored Effect of Measurement. Perspect. Psychol. Sci. *15*, 809–816. https://doi.org/10.1177/1745691620902467.
42. Assel, M., Sjoberg, D.D., and Vickers, A.J. (2017). The Brier score does not evaluate the clinical utility of diagnostic tests or prediction models. Diagn. Progn. Res. *1*, 19. https://doi.org/10.1186/s41512-017-0020-3.
43. Kattan, M.W., and Gerds, T.A. (2018). The index of prediction accuracy: an intuitive measure useful for evaluating risk prediction models. Diagn. Progn. Res. *2*, 7. https://doi.org/10.1186/s41512-018-0029-2.
44. Rousset, A., Dellamonica, D., Menuet, R., Lira Pineda, A., Sabatine, M.S., Giugliano, R.P., Trichelair, P., Zaslavskiy, M., and Ricci, L. (2022). Can machine learning bring cardiovascular risk assessment to the next level? Eur. Heart J. Digit. Health *3*, 38–48. https://doi.org/10.1093/ehjdh/ztab093.
45. Weng, S.F., Reps, J., Kai, J., Garibaldi, J.M., and Qureshi, N. (2017). Can machine-learning improve cardiovascular risk prediction using routine clinical data? PLoS One *12*, e0174944. https://doi.org/10.1371/journal.pone.0174944.
46. Akyea, R.K., Qureshi, N., Kai, J., and Weng, S.F. (2020). Performance and clinical utility of supervised machine-learning approaches in detecting familial hypercholesterolaemia in primary care. npj Digit. Med. *3*, 142–149. https://doi.org/10.1038/s41746-020-00349-5.
47. Desai, R.J., Wang, S.V., Vaduganathan, M., Evers, T., and Schneeweiss, S. (2020). Comparison of Machine Learning Methods With Traditional Models for Use of Administrative Claims With Electronic Medical Records to Predict Heart Failure Outcomes. JAMA Netw. Open *3*, e1918962. https://doi.org/10.1001/jamanetworkopen.2019.18962.
48. Li, Y.M., Jiang, L.C., He, J.J., Jia, K.Y., Peng, Y., and Chen, M. (2020). Machine Learning to Predict the 1-Year Mortality Rate After Acute Anterior Myocardial Infarction in Chinese Patients. TCRM *16*, 1–6. https://doi.org/10.2147/TCRM.S236498.
49. Shwartz-Ziv, R., and Armon, A. (2022). Tabular data: Deep learning is not all you need. Inf. Fusion *81*, 84–90. https://doi.org/10.1016/j.inffus.2021.11.011.
50. Grinsztajn, L., Oyallon, E., and Varoquaux, G. (2022). Why do tree-based models still outperform deep learning on typical tabular data? Adv. Neural Inf. Process. Syst. *35*, 507–520.
51. Snoek, J., Larochelle, H., and Adams, R.P. (2012). Practical Bayesian optimization of machine learning algorithms. In Proceedings of the 25th International Conference on Neural Information Processing Systems - Volume 2 NIPS'12 (Curran Associates Inc.), pp. 2951–2959. https://papers.nips.cc/paper_files/paper/2012/hash/05311655a15b75fab86956663e1819cd-Abstract.html.
52. E. Bartz, T. Bartz-Beielstein, M. Zaefferer, and O. Mersmann, eds. (2023). Hyperparameter Tuning for Machine and Deep Learning with R: A Practical Guide (Springer Nature). https://doi.org/10.1007/978-981-19-5170-1.
53. Bischl, B., Binder, M., Lang, M., Pielok, T., Richter, J., Coors, S., Thomas, J., Ullmann, T., Becker, M., Boulesteix, A.-L., et al. (2023). Hyperparameter optimization: Foundations, algorithms, best practices, and open challenges. WIREs Data Min. Knowl. *13*, e1484. https://doi.org/10.1002/widm.1484.
54. Binder, M., Pfisterer, F., and Bischl, B. (2020). Collecting Empirical Data About Hyperparameters for Data Driven AutoML. In 7th ICML Workshop on Automated Machine Learning.
55. Johnson, J.M., and Khoshgoftaar, T.M. (2021). Medical Provider Embeddings for Healthcare Fraud Detection. SN Comput. Sci. *2*, 276. https://doi.org/10.1007/s42979-021-00656-y.
56. El Emam, K., Pilgram, L., and Liu, D. Sdgm Machine Learning R Package. OSF. https://doi.org/10.17605/OSF.IO/DCJM6.
57. Bouwmeester, W., Zuithoff, N.P.A., Mallett, S., Geerlings, M.I., Vergouwe, Y., Steyerberg, E.W., Altman, D.G., and Moons, K.G.M. (2012). Reporting and Methods in Clinical Prediction Research: A Systematic Review. PLoS Med. *9*, 1–12. https://doi.org/10.1371/journal.pmed.1001221.
58. Botchkarev, A. (2019). Performance Metrics (Error Measures) in Machine Learning Regression, Forecasting and Prognostics: Properties and Typology. IJIKM *14*. 045–076. https://doi.org/10.28945/4184.
59. Morley, S.K., Brito, T.V., and Welling, D.T. (2018). Measures of Model Performance Based On the Log Accuracy Ratio. Space Weather *16*, 69–88. https://doi.org/10.1002/2017SW001669.
60. Yu, S., Eder, B., Dennis, R., Chu, S.-H., and Schwartz, S.E. (2006). New unbiased symmetric metrics for evaluation of air quality models. Atmos. Sci. Lett. *7*, 26–34. https://doi.org/10.1002/asl.125.

61. Mitsakakis, N., Liu, D., and El Emam, K. Sample Size Calculation for Training Ensemble Machine Learning Models on Health Data. OSF. https://doi.org/10.17605/OSF.IO/7BS8Q.
62. Sowkarthika, B., Gyanchandani, M., Wadhvani, R., and Shukla, S. (2024). Data complexity measures for classification of a multi-concept dataset. Multimed Tools Appl *84*, 571–602. https://doi.org/10.1007/s11042-024-18965-8.
63. Silvey, S., and Liu, J. (2024). Sample Size Requirements for Popular Classification Algorithms in Tabular Clinical Data: Empirical Study. J. Med. Internet Res. *26*, e60231. https://doi.org/10.2196/60231.
64. Health Canada, US Federal Drug Agency, and Medicines and Healthcare products Regulatory Agency (2021). Good Machine Learning Practice for Medical Device Development: Guiding principles. https://www.canada.ca/en/health-canada/services/drugs-health-products/medical-devices/good-machine-learning-practice-medical-device-development.html.
65. Klement, W., and El Emam, K. (2023). Consolidated Reporting Guidelines for Prognostic and Diagnostic Machine Learning Modeling Studies: Development and Validation. J. Med. Internet Res. *25*, e48763. https://doi.org/10.2196/48763.
66. El Emam, K., Klement, W., and Malin, B. (2023). Reporting and Methodological Observations on Prognostic and Diagnostic Machine Learning Studies. JMIR *2*, e47995. https://doi.org/10.2196/47995.
67. Collins, G.S., Moons, K.G.M., Dhiman, P., Riley, R.D., Beam, A.L., Van Calster, B., Ghassemi, M., Liu, X., Reitsma, J.B., van Smeden, M., et al. (2024). TRIPOD+AI statement: updated guidance for reporting clinical prediction models that use regression or machine learning methods. BMJ *385*, e078378. https://doi.org/10.1136/bmj-2023-078378.
68. Liu, D., Kababji, S.E., Mitsakakis, N., Pilgram, L., Walters, T., Clemons, M., Pond, G., El-Hussuna, A., and Emam, K.E. (2025). Synthetic Data Generation for Augmenting Small Samples. Preprint at arXiv. https://doi.org/10.48550/arXiv.2501.18741.
69. Heart Disease (1989). Dataset for predicting heart disease presence. UCI Machine Learning Repository. https://doi.org/10.24432/C52P4X.
70. Breast Cancer Wisconsin (1993). Breast cancer diagnostic dataset. UCI Machine Learning Repository. https://doi.org/10.24432/C5DW2B.
71. Chronic Kidney Disease (2015). Kidney disease prediction dataset. UCI Machine Learning Repository. https://doi.org/10.24432/C5G020.
72. Breast Cancer Coimbra (2018). Diagnostic Coimbra breast cancer dataset. UCI Machine Learning Repository. https://doi.org/10.24432/C52P59.
73. Breast Cancer (1988). Breast cancer prediction dataset. UCI Machine Learning Repository. https://doi.org/10.24432/C51P4M.
74. EEG Eye State (2013). EEG measurement dataset. UCI Machine Learning Repository. https://doi.org/10.24432/C57G7J.
75. Adult (1996). Adult income classification dataset. UCI Machine Learning Repository. https://doi.org/10.24432/C5XW20.
76. Bank note (2012). Bank note dataset. UCI Machine Learning Repository. https://doi.org/10.24432/C55P57.
77. Titanic Survival (1912). Titanic Survival Prediction Dataset (Kaggle). https://www.kaggle.com/datasets/hesh97/titanicdataset-traincsv.
78. Stroke (2023). Stroke Prediction Dataset (OSF). https://doi.org/10.17605/OSF.IO/7BS8Q.
79. Colposcopy/green (2017). Digital Colposcopy (green) dataset. UCI Machine Learning Repository. https://doi.org/10.24432/C5C022.
80. Colposcopy/hinselmann (2017). Digital Colposcopy (hinselmann) dataset. UCI Machine Learning Repository. https://doi.org/10.24432/C5C022.
81. Colposcopy/schiller(2017). Digital Colposcopy (schiller) dataset. UCI Machine Learning Repository. https://doi.org/10.24432/C5C022.
82. Thoracic Surgery (2014). Post-operative life expectancy classification prediction dataset. UCI Machine Learning Repository. https://doi.org/10.24432/C5Z60N.
83. Diabetic Retinopathy Debrecen (2014). Diabetic retinopathy prediction dataset. UCI Machine Learning Repository. https://doi.org/10.24432/C5XP4P.
84. Z-Alizadeh Sani (2013). Coronary artery disease prediction dataset. UCI Machine Learning Repository. https://doi.org/10.24432/C5Q31T.
85. Pima Indians Diabetes (1988). Diabetes dataset from Pima Indians (OSF). https://doi.org/10.17605/OSF.IO/7BS8Q.

Patterns, Volume 7

Supplemental information

Sample size calculation for training ensemble machine learning models on health data

Nicholas Mitsakakis, Dan Liu, Thomas Walters, and Khaled El Emam

Supplemental Methods and Notes

Supplemental Figures

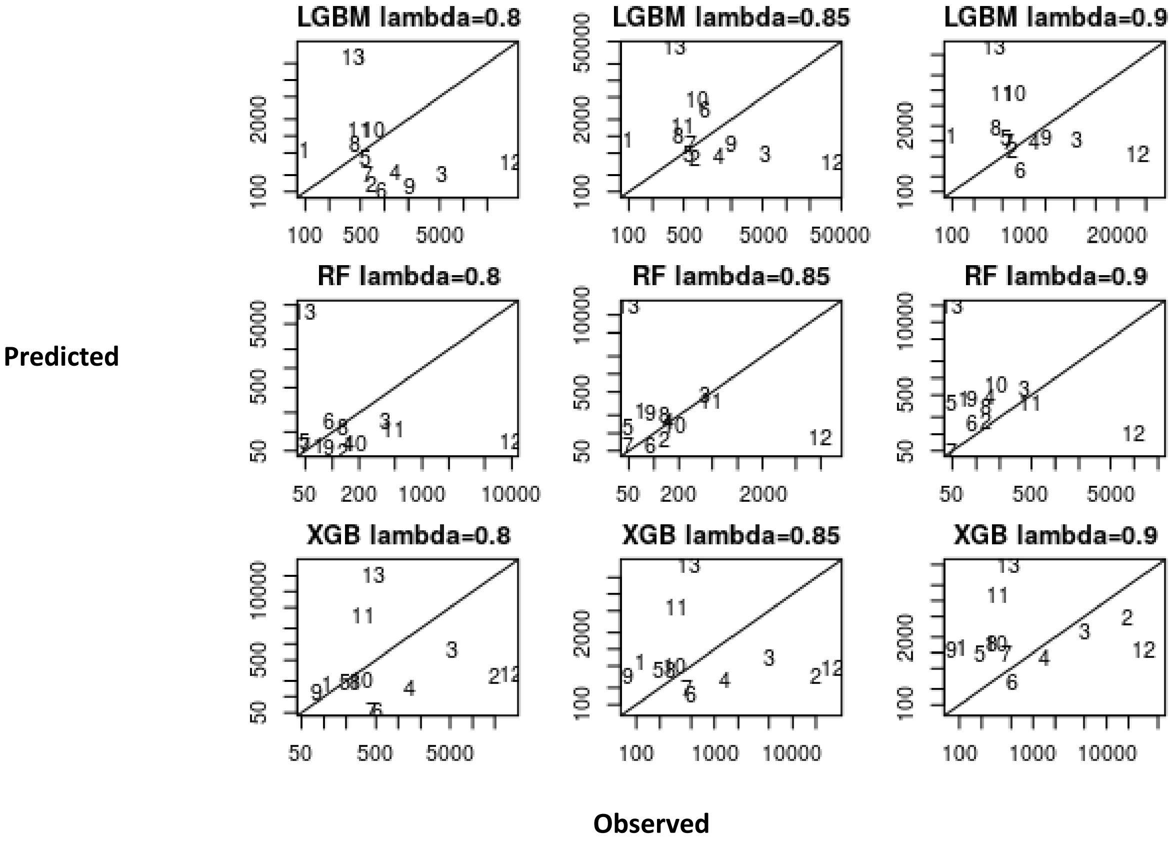

Figure S1: Comparison of the predicted and observed sample sizes for the 13 datasets for different modeling methods and values of lambda, for 80% certainty. Each number in a scatterplot corresponds to a specific dataset, according to the following numbering: 1: cchs, 2: covid, 3: faers, 4: washington2007, 5: texas, 6: nexoid, 7: bsa, 8: california2007, 9: florida2007, 10: newyork2007, 11: washington2008, 12: mimic, 13: born. [S1-S9]

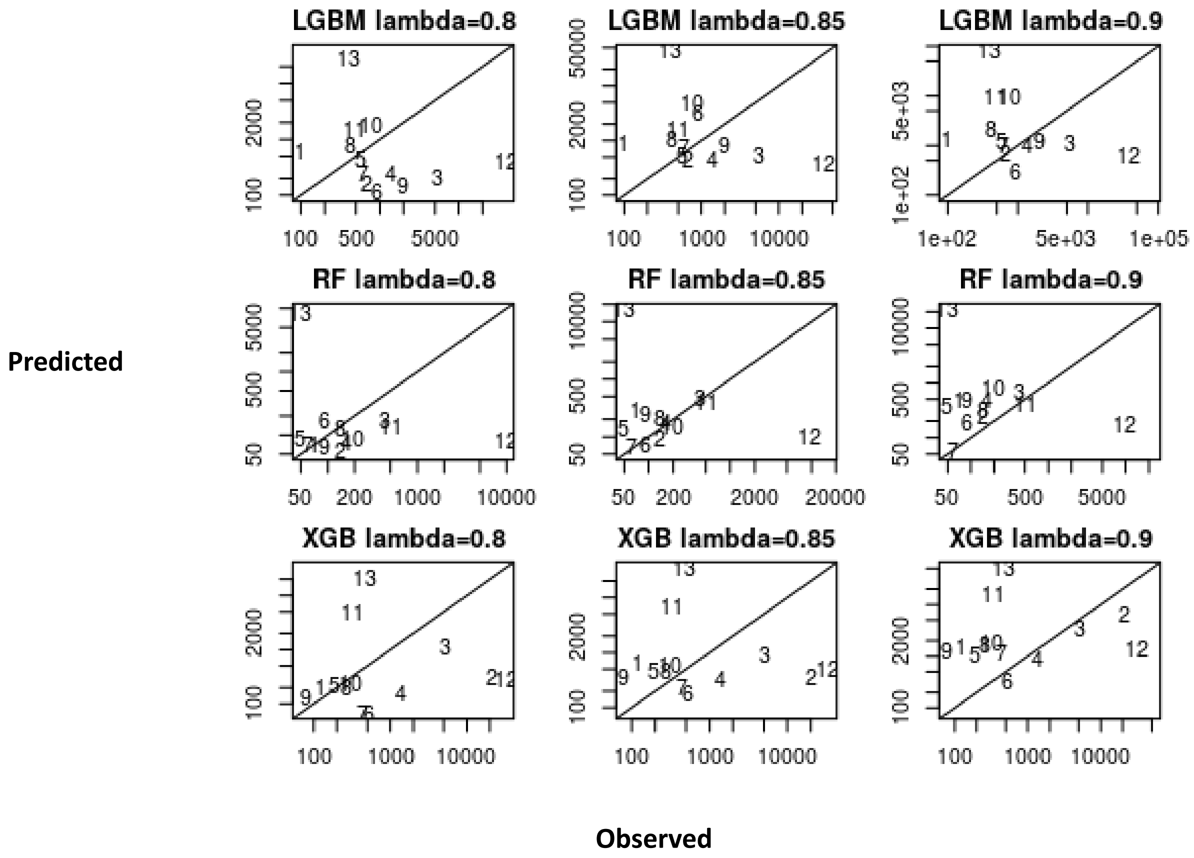

Figure S2: Observed vs predicted required sample sizes for different modeling methods and values of lambda, for 90% certainty.

Supplemental Tables

<table>
<tr><th colspan="5">LGBM</th></tr>
<tr><th>Hyperparameter</th><th>Default</th><th>Lower bound</th><th>Upper bound</th><th>Transform</th></tr>
<tr><td>booster</td><td>1 (gbdt)</td><td>1 (gbdt)</td><td>2 (goss)</td><td rowspan="6">2^learning_rate</td></tr>
<tr><td>max_depth</td><td>6</td><td>1</td><td>15</td></tr>
<tr><td>learning_rate</td><td>log2 (0.3)</td><td>-10</td><td>0</td></tr>
<tr><td>early_stopping_rounds</td><td>7</td><td>7</td><td>30</td></tr>
<tr><td>min_data_in_leaf</td><td>10</td><td>1</td><td>60</td></tr>
<tr><td>num_leaves</td><td>15</td><td>4</td><td>60</td></tr>
<tr><th colspan="5">Random forest</th></tr>
<tr><th>Hyperparameter</th><th>Default</th><th>Lower bound</th><th>Upper bound</th><th>Transform</th></tr>
<tr><td>num.trees</td><td>500</td><td>1</td><td>2000</td><td rowspan="4">round(n^min.node.size), where n is the number of observations</td></tr>
<tr><td>min.node.size</td><td>0.5</td><td>0</td><td>1</td></tr>
<tr><td>max.depth</td><td>15</td><td>1</td><td>50</td></tr>
<tr><td>min.bucket</td><td>10</td><td>1</td><td>60</td></tr>
<tr><th colspan="5">XGBoost</th></tr>
<tr><th>Hyperparameter</th><th>Default</th><th>Lower bound</th><th>Upper bound</th><th>Transform</th></tr>
<tr><td>gamma</td><td>0</td><td>-15</td><td>3</td><td>2^gamma</td></tr>
<tr><td>eta</td><td>log2(0.3)</td><td>-10</td><td>0</td><td>2^eta</td></tr>
<tr><td>max_depth</td><td>6</td><td>1</td><td>15</td><td rowspan="3"></td></tr>
<tr><td>early_stopping_rounds</td><td>7</td><td>7</td><td>30</td></tr>
<tr><td>max_leaves</td><td>15</td><td>4</td><td>60</td></tr>
<tr><td>min_child_weight</td><td>1</td><td>0</td><td>7</td><td>2^min_child_weight</td></tr>
</table>

Table S1: The hyperparameters, their default values, and range for tuning the ensemble models.

Variable	Description	Type	Mean (SD[*]) or level count (% of total size) or number of categories	Missingness (% of the total size)
Date	The date when a case is reported	Numeric (computed as the number of days since 1st January 2020)	348.07 (96.16)	0.00
Age group	Patient's age group in years	Numeric	<20: 17.92%<br>20-29: 20.26%<br>30-39: 17.08%<br>40-49: 14.82%<br>50-59: 13.53%<br>60-69: 8.26%<br>70-79: 3.99%<br>>80: 2.16%	1.99
Gender	Patient's gender	Categorical	Female: 43.39%<br>Male: 50.05%	0.00
Region	Health unit in Canada	Categorical	40	0.00
Exposure	The type of being exposed to someone with COVID-19	Categorical	Close contact: 31.21%<br>Outbreak: 10.78%<br>Travel-related: 1.14%	0.00
Province	Province in Canada where case is reported	Categorical	Ontario: 70.25%<br>Alberta: 29.75%	0.00
Case status	The status of a patient	Categorical	Recovered: 1.48 %<br>Deceased: 98.52%	0.00

[*]SD: standard deviation

Table S2: Descriptive statistics for the COVID-19 dataset [S1]

Variable	Description	Type	Mean (SD) or level count (% of total size)	Missingness (% of the total size)
Age	Patient's age in years	Numeric	47.24 (20.19)	0.00
Sex	Patient's gender	Categorical	Female: 45.84%<br>Male: 54.16%	0.00
Education	Patient's highest level of education	Categorical	< Secondary school graduate: 26.14%<br>Secondary school graduate: 16.93%<br>Some post-secondary education: 6.65%<br>Post-secondary certificate: 48.55%	1.72
Marital status	Patient's marital status	Categorical	Married: 43.41%<br>Common-law: 8.12%<br>Widow/separation/divorce: 19.55%<br>Single/never married: 28.74%	0.17
House income	Total household income from all sources	Numeric	57603.42 (32061.49)	9.59
Household size	Size of entire household	Numeric	2.39 (1.23)	15.66
Immigration	Whether a patient is an immigrant	Categorical	Immigrant: 13.50%<br>Non-immigration: 84.11%	1.81
Smoking	Type of smoking	Categorical	Daily smoking: 17.92%<br>Occasional smoking: 2.64%<br>Always occasional smoking: 1.68%<br>Former daily smoking: 25.76%<br>Former occasional smoking: 14.56%<br>Never smoked: 36.91%	0.00
Obesity	Patient's self-reported BMI	Numeric	25.80 (5.15)	0.00
Hypertension	Whether a patient was diagnosed with hypertension	Categorical	Yes: 20.23%<br>No: 79.46%	0.00
Diabetes	Whether a patient was diagnosed with diabetes	Categorical	Yes: 6.99%<br>No: 92.91%	0.00
Physical activity	Daily energy expenditure	Numeric	2.20 (2.43)	0.00
Fruit and vegetable consumption	Daily consumption of fruits and vegetables	Numeric	4.78 (2.53)	0.00
CANHEART	Whether a patient is in ideal cardiovascular health; this is a sum of the prior six	Categorical	Ideal: 63.95%<br>Non-ideal: 36.05%	0.00

	variables.			

Table S3: Descriptive statistics for the CCHS dataset [S2].

Variable	Description	Type	Mean (SD) or level count (% of total size)	Missingness (% of the total size)
Age	Age group in years	Numeric	0_10: 0.58%<br>10_20: 3.35%<br>20_30: 21.56%<br>30_40: 29.84%<br>40_50: 21.09%<br>50_60: 12.48%<br>60_70: 7.47%<br>70_80: 2.97%<br>80_90: 0.54%<br>90_100: 0.12%<br>100_110: 0.01%	0.00
Sex	Patient’s gender	Categorical	Female: 63.13%<br>Male: 36.53%	0.34
Race	Patient’s race	Categorical	White: 24.19%<br>Hispanic: 1.40%<br>Asian: 1.21%<br>Mixed: 0.96%<br>Black: 0.46%<br>Other: 0.32%	71.45
Smoking	Type of smoking	Categorical	Heavy: 1.68%<br>Medium: 7.64%<br>Light: 4.44%<br>Quit0: 5.54%<br>Quit5: 6.58%<br>Quit10: 9.23%<br>Vape: 5.95%<br>Never smoked: 58.74%	0.19
BMI	Body mass index	Numeric	29.37 (7.81)	0.00
House count	House person count	Numeric	3.14 (1.57)	0.00
Public transport count	Number of public transports used	Numeric	0.38 (1.70)	71.12
Nursing home	Whether it is a nursing home	Categorical	1: 0.07%<br>0: 99.93%	0.00
COVID-19 symptoms	Whether a patient shows symptoms of COVID-19	Categorical	1: 2.04%<br>0: 97.96%	0.00
COVID-19 contact	Whether a patient has close contact with someone infected with COVID-19	Categorical	1: 4.33%<br>0: 95.67%	0.00
Health worker	Whether a patient is a healthcare worker	Categorical	1: 1.79%<br>0: 98.21%	0.00
Asthma	Whether a patient has asthma	Categorical	1: 15.26%<br>0: 84.74%	0.00
Kidney disease	Whether a patient has kidney disease	Categorical	1: 0.36%<br>0: 99.64%	0.00
Liver disease	Whether a patient has liver disease	Categorical	1: 0.21%<br>0: 99.79%	0.00
Heart disease	Whether a patient has heart disease	Categorical	1: 1.87%<br>0: 98.13%	0.00

Lung disease	Whether a patient has lung disease	Categorical	1: 1.45%<br>0: 98.55%	0.00
Diabetes	Whether a patient has diabetes	Categorical	1: 6.17%<br>0: 93.83%	0.00
Hypertension	Whether a patient has hypertension	Categorical	1: 13.83%<br>0: 86.17%	0.00
Outcome	Whether a patient has a high risk of getting infected with COVID-19	Categorical	Yes: 39.20%<br>No: 60.80%	0.00

Table S4: A summary of descriptive statistics for the COVID survival dataset [S3].

Variable	Description	Type	Mean (SD) or level count (% of total size) or number of categories	Missingness (% of the total size)
Outcome	Whether a patient has died	Categorical	Death: 9.94%<br>Non-death: 90.06%	0.00
Event date	Date the adverse event occurred	Numeric<br>(difference from 1/1/2020)	466.52 (827.94)	62.26
Gender	Patient's gender	Categorical	Female: 51.82%<br>Male: 37.85%	10.33
Age	Patient's age in years	Numeric	55.90 (20.80)	33.41
Weight	Patient's weight in kg	Numeric	73.05 (25.70)	74.13
Drug name	Name of medicinal product	Categorical	10,545	0.00
Indication	Medical terminology describing the indication for use	Categorical	4,287	0.00

Table S5: Descriptive statistics for the FAERS dataset [S4].

Variable	Description	Type	Mean (SD) or level count (% of total size) or number of categories	Missingness (% of the total size)
Outcome	Whether a patient's length of stay is greater than 3 days	Categorical	0: 40.42%<br>1: 59.58%	0.00
Age	Patient's age groups	Numeric	0: 11.95%<br>1: 1.66%<br>2: 1.62%<br>3: 1.08%<br>4: 1.39%<br>5: 1.58%<br>6: 1.58%<br>7: 4.84%<br>8: 5.28%<br>9: 4.94%<br>10: 3.75%<br>11: 3.41%<br>12: 3.93%<br>13: 4.96%<br>14: 5.58%<br>15: 5.95%<br>16: 6.47%<br>17: 6.01%<br>18: 5.78%<br>19: 5.32%<br>20: 3.94%<br>21: 2.40%<br>22: 0.21%<br>23: 2.68%<br>24: 2.86%<br>25: 0.54%<br>26: 0.28%	0.00
Sex	Patient's gender	Categorical	Female: 56.25%<br>Male: 37.16%	6.58
Race	Patient's race	Categorical	1 American Indian/Eskimo/Aleut: 0.77%	0.13

			2 Asian or Pacific Islander: 1.68%<br>3 Black: 12.61%<br>4 White: 61.45%<br>5 Other: 23.35%	
Ethnicity	Whether a patient is of Hispanic origin	Categorical	1 Hispanic Origin: 28.14%<br>2 Not of Hispanic Origin: 70.45%	1.41
Location	Patient's mailing address in Texas and contiguous states	Categorical	AR: 0.48%<br>FC: 0.25%<br>LA: 0.21%<br>NM: 0.57%<br>OK: 0.32%<br>TX: 97.21%<br>XX: 0.02%<br>ZZ: 0.92%	0.01
Weekday	The day of week a patient is admitted	Categorical	1 Monday: 16.97%<br>2 Tuesday: 17.22%<br>3 Wednesday: 16.38%<br>4 Thursday: 15.89%<br>5 Friday: 14.98%<br>6 Saturday: 9.42%<br>7 Sunday: 9.14%	0.00
Risk mortality	Risk of mortality score from the All Patient Refined (APR) Diagnosis Related Group (DRG) from the 3M™ APR-DRG Grouper.	Categorical	0 No class specified: 0.10%<br>1 Minor: 60.15%<br>2 Moderate: 20.26%<br>3 Major: 13.26%<br>4 Extreme: 6.22%	0.00
Severity	Severity of illness score from the All Patient Refined (APR) Diagnosis Related Group (DRG) from the 3M™ APR-DRG Grouper.	Categorical	0 No class specified: 0.10%<br>1 Minor: 35.40%<br>2 Moderate: 33.39%<br>3 Major: 22.76%<br>4 Extreme: 8.35%	0.00
DRG	All Patient Refined (APR) Diagnosis Related Group	Categorical	316	0.00

	(DRG) as assigned by 3M APR-DRG Grouper			
Fees	Total non-covered amount of the charge	Numeric	57.51 (1375.47)	0.02

Table S6: Descriptive statistics for the Texas inpatient dataset [S5].

**Variable**	**Description**	**Type**	**Mean (SD) or 1 count (% of total size) or number of categories**	**Missingness (% of the total size)**
Outcome	Whether a patient's length of stay is greater than 3 days	Categorical	Yes: 49.07%<br>No: 50.93%	0.00
Age	Patient's age in years	Numeric	45.58 (28.45)	0.01
Atype	Admission type	Categorical	1: 34.69%<br>2: 18.03%<br>3: 34.23%<br>4: 12.81%<br>5: 0.23%	0.00
Aweekend	Whether admission occurs on a weekend	Categorical	1: 19.32%<br>0: 80.68%	0.00
Died	Whether a patient died during hospitalization	Categorical	1: 1.99%<br>0: 98.01%	0.00
DRG	Diagnosis-related-group (DRG) in effect on discharge date	Categorical	862	0.00
DX1	Primary diagnosis	Categorical	5863	15.14
ZIP	Patient's ZIP code	Categorical	4271	0.06

Table S7: Descriptive statistics for the Washington state hospital discharge dataset [S6].

**Variable**	**Description**	**Type**	**Mean (SD) or 1 count (% of total size) or number of categories**	**Missingness (% of the total size)**
Outcome	Whether the length of stay on a claim is greater than 2.5 days	Categorical	Yes: 57.35%<br>No: 42.65%	0.00
Age	The beneficiary's age	Numeric	1 Under 65: 19.73%<br>2 65- 69: 13.19%<br>3 70-74: 14.65%<br>4 75-79: 15.55%<br>5 80-84: 16.10%<br>6 85 & older: 20.78%	0.00
Gender	The beneficiary's gender	Categorical	1 Male: 56.12%<br>2 Female: 43.88%	0.00

DRG	Diagnostic related groups to which a hospital claim belongs for prospective payment purposes	Categorical	311	0.00
ICD-9	Primary procedure (primarily surgical procedures) performed during the inpatient stay	Categorical	85	47.00
Payment	Quintile value (or code) to which the actual Medicare payment amount on the claim belongs	Categorical	1: 19.97%<br>2: 20.12%<br>3: 20.00%<br>4: 19.82%<br>5: 20.09%	0.00

Table S8: Descriptive statistics for the Basic Stand Alone inpatient claims dataset [S7].

Variable	Description	Type	Mean (SD) or level count (% of total size) or number of categories	Missingness (% of the total size)
Outcome	Whether a patient's length of stay is greater than 2 days	Categorical	1: 74.58%<br>0: 25.42%	0.00
AGE	Patient's age in years	Numeric	45.79 (28.43)	0.01
FEMALE	Whether a patient's gender is female	Categorical	1 Female: 58.69%<br>0 Male: 41.31%	0.01
RACE	Patient's race	Categorical	1 White: 23.86%<br>2 Black: 1.18%<br>3 Hispanic: 2.80%<br>4 Asian or Pacific Islander: 1.31%<br>5 Native American: 0.48%<br>6 Other: 0.02%	70.34
ATYPE	Admission type	Categorical	1 Emergency: 35.59%<br>2 Urgent: 17.80%<br>3 Elective: 33.22%<br>4 Newborn: 12.74%<br>5 Trauma Center: 0.65%	0.00
AWEEKEND	Whether a patient's admission day is on a weekend	Categorical	1 Admitted Saturday - Sunday: 19.50%<br>0 Admitted Monday - Friday: 80.50%	0.00
DRG	Diagnosis Related Group	Categorical	746	0.00
DX1	ICD-9-CM Diagnosis	Categorical	6149	0.00
PAY1	Expected primary payer (Medicare, Medicaid, private insurances, etc.)	Categorical	1 Medicare: 31.21%<br>2 Medicaid: 20.03%<br>3 Private insurance: 42.89%<br>4 Self-pay: 2.80%<br>5 No charge: 0.60%<br>6 Other: 2.46%	0.00
TOTCHG	Total charges	Numeric	26040.52 (43943.20)	0.01
ZIP	Zip code	Categorical	4192	0.00
CHRON1	ICD-9-CM Chronic Condition Indicators	Categorical	1 Chronic condition: 35.16%<br>0 Non-chronic condition: 64.82%	0.01
CHRONB1	Chronic Condition Indicators - body system	Categorical	18	0.01

PCLASS1	Procedure Classes Refined for ICD-10-PCS procedure codes	Categorical	1 Minor diagnostic: 6.66%<br>2 Minor therapeutic: 24.98%<br>3 Major diagnostic: 0.44%<br>4 Major Therapeutic: 31.07%	36.84
CM_ALCOH	AHRQ comorbidity measure for ICD-9-CM codes: alcohol abuse	Categorical	1 Comorbidity is present: 3.01%<br>0 Comorbidity is not present: 96.99%	0.00
CM_DEPRE	AHRQ comorbidity measure for ICD-9-CM codes: depression	Categorical	1 Comorbidity is present: 6.31%<br>0 Comorbidity is not present: 93.69%	0.00
CM_HTN_C	AHRQ comorbidity measure for ICD-9-CM codes: hypertension (combine uncomplicated and complicated)	Categorical	1 Comorbidity is present: 28.69%<br>0 Comorbidity is not present: 71.31%	0.00
CM_OBESE	AHRQ comorbidity measure for ICD-9-CM codes: obesity	Categorical	1 Comorbidity is present: 5.55%<br>0 Comorbidity is not present: 94.45%	0.00

Table S9: Descriptive statistics for the hospital Washington dataset [S6].

Variable	Description	Type	Mean (SD) or level count (% of total size) or number of categories	Missingness (% of the total size)
Outcome	Whether a patient's length of stay is greater than 3 days	Categorical	1: 54.10%<br>0: 45.90%	0.00
AGE	Patient's age in years	Numeric	44.59 (28.58)	0.95
FEMALE	Whether a patient's gender is female	Categorical	1 Female: 57.24%<br>0 Male: 39.85%	2.91
RACE	Patient's race	Categorical	1 White: 46.72%<br>2 Black: 7.27%<br>3 Hispanic: 28.52%<br>4 Asian or Pacific Islander: 7.07%<br>5 Native American: 0.07%<br>6 Other: 2.04%	8.31
AWEEKEND	Whether a patient's admission day is on a weekend	Categorical	1 Admitted Saturday - Sunday: 20.39%<br>0 Admitted Monday - Friday: 79.61%	0.00
DRG	Diagnosis Related Group	Categorical	746	0.00
DX1	ICD-9-CM Diagnosis	Categorical	8548	0.00
PAY1	Expected primary payer (Medicare, Medicaid, private insurances, etc.)	Categorical	1 Medicare: 31.13%<br>2 Medicaid: 25.57%<br>3 Private insurance: 34.79%<br>4 Self-pay: 3.41%<br>6 Other: 5.09%	0.02
TOTCHG	Total charges	Numeric	45065.28 (78294.38)	12.00
CHRON1	ICD-9-CM Chronic Condition Indicators	Categorical	1 Chronic condition: 34.22%<br>0 Non-chronic condition: 65.78%	0.00
CHRONB1	Chronic Condition Indicators - body system	Categorical	18	0.00
PCLASS1	Procedure Classes Refined for ICD-10-PCS procedure codes	Categorical	1 Minor diagnostic: 9.65%<br>2 Minor therapeutic: 28.12%<br>3 Major diagnostic: 0.46%<br>4 Major Therapeutic: 26.37%	35.40
CM_ALCOH	AHRQ comorbidity measure for ICD-9-CM codes: alcohol abuse	Categorical	1 Comorbidity is present: 3.84%<br>0 Comorbidity is not present: 96.16%	0.00

CM_DEPRE	AHRQ comorbidity measure for ICD-9-CM codes: depression	Categorical	1 Comorbidity is present: 5.80%<br>0 Comorbidity is not present: 94.20%	0.00
CM_HTN_C	AHRQ comorbidity measure for ICD-9-CM codes: hypertension (combine uncomplicated and complicated)	Categorical	1 Comorbidity is present: 33.31%<br>0 Comorbidity is not present: 66.69%	0.00
CM_OBESE	AHRQ comorbidity measure for ICD-9-CM codes: obesity	Categorical	1 Comorbidity is present: 7.23%<br>0 Comorbidity is not present: 92.77%	0.00

Table S10: Descriptive statistics for the hospital California dataset [S6].

Variable	Description	Type	Mean (SD) or level count (% of total size) or number of categories	Missingness (% of the total size)
Outcome	Whether a patient's length of stay is greater than 3 days	Categorical	1: 60.46%<br>0: 39.54%	0.00
AGE	Patient's age in years	Numeric	51.23 (27.04)	0.00
FEMALE	Whether a patient's gender is female	Categorical	1 Female: 56.02%<br>0 Male: 43.98%	0.00
RACE	Patient's race	Categorical	1 White: 65.53%<br>2 Black: 16.92%<br>3 Hispanic: 13.28%<br>4 Asian or Pacific Islander: 0.76%<br>5 Native American: 0.27%<br>6 Other: 2.47%	0.76
ATYPE	Admission type	Categorical	1 Emergency: 54.15%<br>2 Urgent: 16.43%<br>3 Elective: 20.73%<br>4 Newborn: 8.17%<br>5 Trauma Center: 0.52%	0.00
AWEEKEND	Whether a patient's admission day is on a weekend	Categorical	1 Admitted Saturday - Sunday: 19.51%<br>0 Admitted Monday - Friday: 80.49%	0.00
DRG	Diagnosis Related Group	Categorical	861	0.00
DX1	ICD-9-CM Diagnosis	Categorical	7380	0.00
PAY1	Expected primary payer (Medicare, Medicaid, private insurances, etc.)	Categorical	1 Medicare: 42.71%<br>2 Medicaid: 17.50%<br>3 Private insurance: 27.52%<br>4 Self-pay: 6.23%<br>5 No charge: 2.28%<br>6 Other: 3.77%	0.00
TOTCHG	Total charges	Numeric	33604.48 (52812.95)	0.01
ZIP	Zip code	Categorical	14729	0.00

Note: SD: standard deviation

Table S11: Descriptive statistics for the hospital Florida dataset [S6].

Variable	Description	Type	Mean (SD) or level count (% of total size) or number of categories	Missingness (% of the total size)
Outcome	Whether a patient's length of stay is greater than 3 days	Categorical	1: 61.82%<br>0: 38.18%	0.00
AGE	Patient's age in years	Numeric	48.87 (27.36)	0.00
FEMALE	Whether a patient's gender is female	Categorical	1 Female: 56.68%<br>0 Male: 43.32%	0.00
RACE	Patient's race	Categorical	1 White: 56.73%<br>2 Black: 17.43%<br>3 Hispanic: 13.56%<br>4 Asian or Pacific Islander: 3.38%<br>5 Native American: 1.01%<br>6 Other: 5.90%	1.98
ATYPE	Admission type	Categorical	1 Emergency: 60.22%<br>2 Urgent: 9.86%<br>3 Elective: 20.86%<br>4 Newborn: 8.90%<br>5 Trauma Center: 0.00%	0.16
AWEEKEND	Whether a patient's admission day is on a weekend	Categorical	1 Admitted Saturday - Sunday: 19.26%<br>0 Admitted Monday - Friday: 80.74%	0.00
DRG	Diagnosis Related Group	Categorical	863	0.00
DX1	ICD-9-CM Diagnosis	Categorical	7956	0.00
PAY1	Expected primary payer (Medicare, Medicaid, private insurances, etc.)	Categorical	1 Medicare: 36.08%<br>2 Medicaid: 23.69%<br>3 Private insurance: 32.38%<br>4 Self-pay: 5.44%<br>5 No charge: 0.17%<br>6 Other: 2.23%	0.00
TOTCHG	Total charges	Numeric	24628.84 (43545.43)	0.01
ZIP	Zip code	Categorical	10814	0.00
CHRON1	ICD-9-CM Chronic Condition Indicators	Categorical	1 Chronic condition: 40.86%<br>0 Non-chronic condition: 59.14%	0.00
CHRONB1	Chronic Condition Indicators - body system	Categorical	18	0.00

PCLASS1	Procedure Classes Refined for ICD-10-PCS procedure codes	Categorical	1 Minor diagnostic: 15.19%<br>2 Minor therapeutic: 31.30%<br>3 Major diagnostic: 0.54%<br>4 Major Therapeutic: 25.86%	27.12

Table S12: Descriptive statistics for the hospital New York dataset [S6].

Variable	Description	Type	Mean (SD) or level count (% of total size) or number of categories	Missingness (% of the total size)
Birth weight	Whether a newborn baby has low birthweight (<2,500 grams)	Categorical	1: 7.01%<br>0: 92.99%	0.00
Gestational age	Gestational age of a newborn baby	Numeric	1 < 34 weeks: 2.32%<br>2 34-36 weeks: 6.00%<br>3 37-38 weeks: 27.15%<br>4 39-41 weeks: 64.11%<br>5 >=42 weeks: 0.43%	0.00
Maternal age	Maternal age in years at time of stillbirth or live birth	Numeric	1 <= 19: 2.15%<br>2 20-34: 74.12%<br>3 35-39: 19.31%<br>4 >=40: 4.36%	0.06
Maternal BMI	Maternal pre-pregnancy body mass index	Numeric	1 <18.5: 4.53%<br>2 18.5-24.9: 43.44%<br>3 25-29.9: 20.22%<br>4 >=30: 15.86%	15.94
Parity	Total number of pregnancies a mother has experienced	Numeric	0: 42.82%<br>1: 34.57%<br>2: 13.98%<br>3: 4.64%<br>>=4: 2.89%	1.10
Preterm birth	Number of previous preterm pregnancies	Numeric	0: 93.57%<br>1: 4.41%<br>2: 0.71%<br>3: 0.13%<br>>=4: 0.05%	1.13
Abortions	Number of previous abortions	Numeric	0: 65.77%<br>1: 20.85%<br>2: 7.25%<br>3: 2.50%<br>>=4: 1.50%	2.13
Smoking	Maternal smoking status at time of admission	Categorical	Yes: 7.64%<br>No: 88.14%	4.21
Alcohol	Alcohol exposure in pregnancy	Categorical	Yes: 2.23%<br>No: 92.54%	5.23
Prenatal screening	Whether a mother has prenatal screening during pregnancy	Categorical	Yes: 66.30%<br>No: 33.70%	0.00

Addiction	Mental health concern regarding addiction	Categorical	Yes: 0.60%<br>No: 93.72%	5.68
Anxiety	Mental health concern regarding anxiety	Categorical	Yes: 8.99%<br>No: 85.33%	5.68
Depression	Mental health concern regarding depression	Categorical	Yes: 7.58%<br>No: 86.74%	5.68
Diabetes	Maternal health condition regarding diabetes	Categorical	Yes: 1.00%<br>No: 93.27%	5.73
Genetics	Maternal health condition regarding genetics	Categorical	Yes: 0.00%<br>No: 94.26%	5.73
Cocaine drug	Drug exposure to Cocaine in pregnancy	Categorical	Yes: 0.25%<br>No: 94.65%	5.10
Hallucinogens drug	Drug exposure to Hallucinogens in pregnancy	Categorical	Yes: 0.02%<br>No: 94.88%	5.10
Opioids drug	Drug exposure to Opioids in pregnancy	Categorical	Yes: 0.42%<br>No: 94.48%	5.10

Table S13: Descriptive statistics for the BORN dataset [S8].

Variable	Description	Type	Mean (SD) or level count (% of total size) or number of categories	Missingness (% of the total size)
Readmission	Whether a patient is re-admitted to ICU	Categorical	1 Yes: 21.01%<br>0 No: 78.99%	0.00
Age	Patient's age in the time of first admission	Numeric	63.43 (16.16)	0.00
Ethnicity	Patient's ethnicity group	Categorical	38	15.14%
Admission type	Patient's admission type	Categorical	Elective: 18.10%<br>Emergency: 78.52%<br>Urgent: 3.37%	0.00
Heart rate	Vital sign for heart rate	Numeric	87.86 (15.89)	0.47%
NT-proBNP	Lab test for N-terminal prohormone of brain natriuretic peptide	Numeric	4.10 (1.17)	43.55%
Creatinine	Lab test for serum creatinine	Numeric	4.10 (1.17)	43.54%
Bun	Lab test for blood urea nitrogen	Numeric	4.10 (1.17)	43.53%
Potassium	Lab test for potassium	Numeric	4.10 (1.17)	43.52%
Cholesterol	Lab test for cholesterol	Numeric	4.10 (1.17)	43.54%

Table S14: Descriptive statistics for the MIMIC-III dataset [S9].

	**LGBM**			**RF**			**XGB**		
**lambda**	0.80	0.85	0.90	0.80	0.85	0.90	0.80	0.85	0.90
cchs	-73.45	1.88	26.22	-46.09	8.44	104.81	-58.71	0.25	260.81
covid	-47.52	77.56	83.42	-23.82	14.51	166.26	-30.13	59.16	395.98
faers	-47.52	77.56	83.42	-23.82	14.51	166.26	-30.13	59.16	395.98
washington2007	-47.52	77.56	83.42	-23.82	14.18	104.81	-30.13	59.16	395.98
texas	-73.45	77.56	26.22	-46.09	8.44	104.81	-58.71	0.25	260.81
nexoid	-47.52	1.88	83.42	-46.09	14.51	166.26	-30.13	59.16	395.98
bsa	-54.18	62.98	53.40	-46.09	8.44	166.26	-30.13	59.16	395.98
california2007	-73.45	1.88	26.22	-46.09	8.44	140.23	-58.71	0.25	260.81
florida2007	-47.52	77.56	83.42	-32.55	8.44	104.81	-58.71	0.25	260.81
newyork2007	-73.45	1.88	26.22	-23.82	14.51	104.81	-55.87	2.68	261.76
washington2008	-73.45	1.88	26.22	-23.82	14.51	166.26	-58.71	0.25	260.81
mimic	-47.52	77.56	83.42	-23.82	14.51	166.26	-30.13	59.16	395.98
born	-73.45	1.88	26.22	-46.09	8.44	104.81	-58.71	0.25	260.81
**default**	**-66.8**	**16.5**	**56.2**	**-37.4**	**8.8**	**130.8**	**-33**	**56.7**	**395**

Table S15: The percentage mRE sensitivity to the dataset at 80% certainty.

	LGBM			RF			XGB		
lambda	0.80	0.85	0.90	0.80	0.85	0.90	0.80	0.85	0.90
cchs	-68.58	8.31	36.11	-40.16	8.58	127.17	-50.07	13.99	313.33
covid	-41.28	92.19	94.50	-17.87	11.13	170.17	-22.35	81.06	479.88
faers	-41.28	92.19	94.50	-17.87	11.13	170.17	-22.35	81.06	479.88
washington2007	-41.28	92.19	94.50	-17.87	10.41	127.17	-22.35	81.06	479.88
texas	-68.58	92.19	36.11	-40.16	8.58	127.17	-50.07	13.99	321.97
nexoid	-41.28	8.31	94.50	-40.16	11.13	170.17	-22.35	81.06	479.88
bsa	-48.55	75.18	65.85	-40.16	8.58	170.17	-22.35	81.06	479.88
california2007	-68.58	8.31	36.11	-40.16	8.58	159.94	-50.07	20.44	313.33
florida2007	-41.28	92.19	94.50	-29.95	8.58	127.17	-50.07	13.99	313.33
newyork2007	-68.58	8.31	36.11	-17.87	11.13	127.17	-49.70	13.99	313.33
washington2008	-68.58	8.31	36.11	-17.87	11.13	170.17	-50.07	13.99	313.33
mimic	-41.28	92.19	94.50	-17.87	11.13	170.17	-22.35	81.06	479.88
born	-68.58	8.31	36.11	-40.16	8.58	127.17	-50.07	13.99	313.33
default	-61.3	25.3	64.8	-28.1	9.3	137.4	-22.7	74.6	471.2

Table S16: The percentage mRE sensitivity to the dataset at 90% certainty.

	LGBM			RF			XGB		
**lambda**	0.80	0.85	0.90	0.80	0.85	0.90	0.80	0.85	0.90
cchs	449.51	214.95	287.82	139.87	51.15	163.90	740.41	362.28	395.97
covid	491.24	222.31	381.43	89.14	51.15	260.03	608.45	362.28	419.38
faers	441.04	214.95	287.82	89.14	57.65	260.03	740.41	362.28	419.38
washington2007	441.04	222.31	381.43	105.01	57.65	214.94	608.45	364.81	419.38
texas	491.24	222.31	381.43	139.87	51.15	163.90	740.41	420.48	418.39
nexoid	441.04	215.36	381.43	139.87	57.65	260.03	608.45	420.48	419.38
bsa	491.24	222.31	381.43	139.87	57.65	260.03	662.72	420.48	419.38
california2007	491.24	222.31	319.76	139.87	53.28	260.03	740.41	420.48	395.97
florida2007	441.04	222.31	381.43	139.87	51.15	163.90	740.41	420.48	395.97
newyork2007	491.24	214.95	287.82	89.14	57.65	163.90	740.41	420.48	419.38
washington2008	491.24	214.95	287.82	89.14	57.65	260.03	608.45	362.28	395.97
mimic	441.04	214.95	287.82	89.14	51.15	163.90	608.45	362.28	395.97
born	441.04	214.95	287.82	89.14	51.15	163.90	608.45	362.28	395.97
**default**	**482**	**222**	**345**	**121**	**55**	**202**	**681**	**418**	**397**

Table S17: The percentage mALQE sensitivity to the dataset at 80% certainty.

	LGBM			RF			XGB		
**lambda**	0.80	0.85	0.90	0.80	0.85	0.90	0.80	0.85	0.90
cchs	383.45	219.29	276.08	113.28	35.77	168.17	602.46	356.05	479.81
covid	480.32	246.61	408.59	97.90	39.42	266.97	436.15	356.05	495.36
faers	383.45	219.29	290.14	97.90	49.28	266.97	602.46	356.05	495.36
washington2007	396.97	246.61	408.59	101.56	49.28	224.85	436.15	396.00	495.36
texas	480.32	246.61	408.59	113.28	35.77	168.17	602.46	396.00	495.36
nexoid	383.45	219.29	408.59	113.28	49.28	266.97	436.15	396.00	495.36
bsa	480.32	246.61	408.59	113.28	49.28	266.97	480.18	396.00	495.36
california2007	480.32	246.61	276.08	113.28	35.77	266.97	602.46	396.00	479.81
florida2007	383.45	246.61	408.59	113.28	35.77	168.17	602.46	382.74	479.81
newyork2007	480.32	219.29	276.08	97.90	49.28	168.17	602.46	396.00	486.54
washington2008	480.32	226.63	276.08	97.90	49.28	266.97	436.15	356.05	479.81
mimic	383.45	219.29	276.08	97.90	35.77	168.17	436.15	356.05	479.81
born	383.45	219.29	276.08	97.90	35.77	168.17	436.15	356.05	479.81
**default**	**465**	**239**	**390**	**109**	**45**	**203**	**549**	**369**	**489**

Table S18: The percentage mALQE sensitivity to the dataset at 90% certainty.

Dataset #	Data Name	Description	Number of Records	Number of Variables
1	Adult	The census income data from 1994 Census database	44842	13
2	BankNote	Data of images that were taken for the evaluation of tan authentication procedure for banknotes.	1371	5
4	Breast Cancer Wisconsin	Diagnostic Wisconsin Breast Cancer Database	683	9
4	Breast Cancer Coimbra	Diagnostic Coimbra Breast Cancer Database	116	9
5	Breast Cancer	This data is provided by the Oncology Institute to predict the breast cancer.	227	10
6	Chronic Kidney Disease	This dataset is collected in Apollo Hospital, India. It can be used to predict the chronic kidney disease.	209	21
7	Heart Disease	The Cleveland heart database	303	13
8	Colposcopy/green	The three modalities of data are dedicated to determining two classes of the colposcopic sequences (bad, good).	98	56
9	Colposcopy/hinselmann		97	56
10	Colposcopy/schiller		92	56
11	Diabetic Mellitus	The data is dedicated to determining the type of diabetic mellitus.<br>This dataset is from OpenML.	281	97
12	Diabetic Retinopathy	This dataset contains features extracted from the Messidor image set to predict whether an image contains signs of diabetic retinopathy or not.	1151	19
13	EEGb Eye State	All data is from one continuous EEG measurement with the Emotiv EEG Neuroheadset. The data set consists of 14 EEG values and a value indicating the eye state.	14980	14
14	Stroke	Health care database to predict stroke	29072	10
15	Thoracic Surgery	The data is dedicated to the classification problem related to the post-operative life expectancy in lung cancer patients.	470	16
16	Titanic_train	The train dataset on Kaggle is a subset of the passenger information on Titanic. This dataset is used to predict whether the passenger survived or not. This dataset is from Kaggle.	891	8
17	Z-Alizadeh Sani	This data is used to predict two possible categories of CAD (normal or not normal).	303	56

Table S19: Datasets [S10-S26] used for performing the sensitivity analysis of mean standardized entropy. Unless otherwise stated, the datasets are available from the UCI repository.

Dataset #	Deviation in Standardized Mean Entropy					
	**-0.5**	**-0.25**	**-0.1**	**0.1**	**0.25**	**0.5**
1	0.9	0.0	0.0	-41.7	-27.8	-34.2
2	40.5	40.5	-17.7	-2.0	-11.3	-11.3
3	38.8	38.8	1.0	-9.9	-8.7	-8.7
4	95.9	94.2	94.2	40.1	26.0	27.7
5	1736.1	0.0	0.0	0.0	-25.0	-11.8
6	0.0	0.0	0.0	0.0	0.0	0.0
7	46.3	17.6	8.8	82.1	82.1	82.1
8	-33.5	-29.2	-22.7	-0.1	-17.8	-17.8
9	5141.9	-0.8	0.0	0.0	0.0	-20.1
10	3126.6	0.0	0.0	-19.5	-12.0	-26.7
11	3977.4	0.0	0.0	-43.6	-27.8	-35.1
12	95.9	94.2	94.2	40.1	26.0	27.7
13	26.4	26.9	26.9	-9.2	8.5	63.6
14	16.4	16.4	1.6	-12.1	-13.9	-13.9
15	-9.0	0.0	0.0	-41.3	-16.9	-24.1
16	22.6	22.6	0.6	-7.2	-8.8	-8.8
17	38.6	38.6	-21.9	-0.2	-8.9	-8.9

Table S20: The discrepancy results for the required sample size estimation corresponding to deviation from mean entropy value, as input to the sample size calculator. Discrepancy is measured by the percent relative error given by 100*(n’-n)/n, where n, n’ denotes the calculated sample size when the measured and deviated entropy value is used, respectively.

Supplemental Notes

Previous Sample Size Estimation Studies

Figueroa et at [S27], discusses a learning curve fitting approach, proposed also by Mukherjee et al [S28]. The authors used an inverse power law method to estimate the effect of training data size and accuracy of a classifier. The method requires the estimate of a performance measure for training data of different sizes. The authors use 3 large datasets to validate their method. It can be used in real applications in order to determine if additional data would be beneficial in terms of significantly increasing the performance of the classifier. However, it cannot be used for determining a-priori and without training a model on existing data what the required sample size of the training data would be.

Raudys and Jain [S29] reviewed a number of statistical pattern recognition methods for binary classification, focusing on the effect of training and testing sample size on feature selection and error estimation. The investigation includes mainly parametric methods (FDA, QDA, Parzen Window classifier) as well as KNN. They summarize the number of observations in the smallest class needed so that the classification error estimated by the sample is less than 50% larger than the theoretical classification error (produced by an infinite size sample). This investigation relies on normality and other assumptions. It includes a series of theoretical results that can be used for specific cases and under specific assumptions. As such, generalization of these results requires a lot of caution.

Fukunara and Hayes [S30] investigate the effect of sample size on the performance of a binary classifier. They consider the cases of Linear and Quadratic classifiers under the assumption of normally distributed data. They provide mathematical expressions of how sample size of the training and test set affect the estimation of important parameters of the underlying distribution (such as means and covariate matrix), which are needed for the classifiers under investigation.

Larracy and colleagues [S31] used a simulation to investigate the effect of training sample size, validation framework and feature selection method, under different scenarios of discriminability in binary classification problems. They also assess the performance of learning curves as tools for forecasting the improvement of ML models with larger training data sizes. The study confirms that nested CV is the preferred validation method when the sample size is small. However, it has various limitations: the data simulation mechanism is simplistic (all features are normally distributed with equal standard deviations, while their mean determines the two classes, and the discriminability between them). The only ML method that is used is linear SVM. Sample sizes under consideration range from 10 to 600, where the performance of the models seems to plateau.

van der Ploeg et al [S32] use simulated population data generated based on three existing clinical datasets and investigated the performance of four ML models, as well as logistic regression, when trained and tested on subsets of the population sets with varying sizes. They reported “optimism” of each model (i.e., how well the model performed in the training data, with comparison to the test data), concluding that ML modeling methods are “data hungry” when compared to traditional logistic regression. The study has some limitations including ignoring the essential step of tuning for the ML models, using a limited set of simulated population dataset scenarios, and using optimism as a measure of model’s performance is of limited utility in practice, as the “test” performance can be directly evaluated using resampling methods (e.g. cross-validation).

Vabalas and colleagues [S33] investigate how the settings around training and model assessment affect the performance of the model and the accuracy of the assessment. They explore the literature on the topic focusing on the application of ML methods in studies on autism, using meta-analysis on the effect of sample size and model accuracy. Furthermore, they perform simulations, on varying sample sizes (n=200 to 1000), two-class data with 50 normally distributed features (similar to Laracy et al.), distinguishing two scenarios of discriminability (all features being noise vs 10 discriminating features and 40 noise) and 3 approaches for feature selection. They use SVM as their ML model, and distinguish between a number of validation strategies (split, k-fold CV, nested CV). They show that for small sample sizes, nested CV gives a more accurate assessment of model performance than simple CV (where feature selection and tuning

happens a priori). In their simulations, the models' performance seems to plateau at a sample size of around 600.

McNemara and colleagues [S34] also study the effect of sample size on the performance of ML models, using an extensive simulation. They are particularly interested in the robustness of the models under training against measurement error in the predictors, in addition to mislabeling error, and presence of features that are unrelated with the outcome. They compare the performance of gradient boosted machines, random forests, logistic regression with regularization (elastic net), using the Area Under the Curve and binary log loss as performance measures. They found that the tree-based models achieve superior performance that plateaus at around n=3000. Overall, the study is well designed and provides useful insights, however the simulation data mechanism is limited to a couple of specific approaches and results cannot be generalized to an arbitrary setting of real data.

Zantvoort et al. [S35] evaluated prognostic improvement as data size increases for a single mental health dataset, also examining the impact of the number of features and the complexity of the features. They provide guidance on appropriate minimal sample sizes in this domain.

In addition, the following studies use specific data types different from clinical data.

Acharjee and colleagues [S36] use a simulation to validate a number of variable selection methods coupled with random forests. Their method aims to guide study design and required size of training data, but mainly focus on "omics" applications (genomics, metabolomics) and it is uncertain if these approaches can be extended to different fields.

D'souza and colleagues [S37] investigate the effect of the "structural optimization" of CNN models on the accuracy, for classification problems, under different sample sizes. Various candidate structures were generated over the choices of the number of layers and weights with a constrained value of the Vapkin-Chervonenkis (VC) dimension (a measure of the complexity of the model, calculated by an expression provided in Bartlet et al [S38]). Their analysis uses samples of varying sizes from three large image datasets (MNIST, CIFAR10, mitosis). Along other details, they conclude that the influence of the network structure is larger when the training sample size is small. Their study can provide various insights on the importance of finding an optimal network structure, under different sample size constraints. However, the applicability of these findings to other types of ML models is very limited, considering also the very limited number of datasets used (only 3) and variety of training data sizes (100, 500, 1000).

In the work of Beleitas et al [S39] the effect of the size of both the training and the test sample on the accuracy and precision of the performance measure is investigated. The authors use real and simulated sprectroscopy data and they apply an LDA model using 10 latent variables (derived by the application of a partial least square model) as features. They focus exclusively on multiclass sensitivity as performance measure, and they assess how well a learning curve is able to forecast the performance of a model with a larger training dataset. They provide a simple calculation of required sample size for achieving desired precision (as measured by the width of the confidence interval of the sensitivity). They primarily focus on very small sample sizes (n=20), which are more relevant in the field of mass spectrometry analysis. Their analysis provides some interesting insights, but it has limited generalizability to the general context of sample size requirements in ML applications.

Balki et al. [S40] investigate the issue of sample size requirements and its effect on model performance in the field of medical imaging. They conduct a systematic review and find a limited number of studies (n=18) that assess the performance of their models with respect to the size of training data. An even smaller number of studies (n=4) attempt to develop a methodology for specifying the required sample size for obtaining the desired level of accuracy. These methodological approaches can be divided into pre-hoc (model based) and post-hoc (curve-fitting). The former use theoretical findings for specific models (e.g. 1-hidden layer feed-forward neural network) and construct mathematical rules and expressions for guiding the determination of required sample size. The latter are used for fitting a curve of model's accuracy on training data size, which can then be used for forecasting the required size for obtaining a desired performance level. This type of methodology is relevant and useful in the setting of medical imaging where the size of training data depends on the capacity to label the images. The required sample size is not known a-priori, but it can be determined after training and assessing the model on small size data and as such obtaining points to be used in curve fitting, and subsequent forecasting of the required sample

size. Although the review is very thorough and comprehensive, its findings cannot necessarily generalize to the general case of ML applications on non-image data. Nevertheless, they illustrate the scarcity of sound and robust methodological approaches for sample size determination for ML applications, and the need for further development.

Details on the population datasets

The following are the details for the datasets that were used in this study:

Canadian COVID-19

The first dataset is the Canadian COVID-19 dataset from the Public Health Agency of Canada [S1]. It contains over 1 million health records of individuals who have tested positive for COVID-19. We are interested in fitting a model that predicts mortality caused by COVID-19. The binary outcome of interest is derived from the case status in the dataset, and a value of 1 is assigned if the patient has died due to COVID-19 while a value 0 is assigned if the patient has recovered. The selected predictors for modeling include the following variables: date, age group, gender, region, exposure, province. Table S2 presents an overview of the variables that are included in the binary model.

Canadian Community Health Survey

The CCHS data is a cross-sectional telephone survey administered by Statistics Canada that collects information on health status, health care utilization and health determinants of Canadians [S2]. This dataset is a pooled version of survey data from 2001 to 2013, and variables we are using are presented in Table S3. The model outcome is cardiovascular health and the covariates are age, sex, education, house income, household size, immigration as predictors to predict the ideal state of cardiovascular health using variables from the dataset [S41]. To assess cardiovascular health, we follow the definition of ideal cardiovascular health introduced by the American Heart Association to calculate the Cardiovascular Health in Ambulatory Care Research Team (CANHEART) health index score, which is determined by 7 health factors including smoking, obesity, hypertension, diabetes, physical activity, and fruit and vegetable consumption [S42]. The final CANHEART index score ranges from 0 (worst) to 6 (best). The outcome is assigned to be 1 if the score is above 3.5 [S43], which is considered to be an intermediate or ideal state of cardiovascular health, and 0 otherwise.

COVID-19 Survival

The COVID-19 survival dataset [S3] that is used in the study is a web-based survey data collected by the research team by Nexoid, a company in United Kingdom. They collect demographic, socioeconomic and health-related information of individuals to predict two crucial aspects related to COVID-19: the probability of being infected with COVID-19 as well as probability of mortality associated with COVID-19. In our study, we focus on the probability of COVID-19 infection using the important demographic, behavioral and health factors including age, sex, race, smoking, nursing home, COVID-19 symptoms, COVID-19 contact, health worker, and the presence of comorbidities such as asthma, kidney disease, liver disease, heart disease, lung disease, diabetes, and hypertension. The outcome of interest is determined by the risk scores of getting infected with COVID-19. The patients whose risk scores exceed the mean risk score are considered as having a high risk of contracting COVID-19, while those with scores below the mean are classified as having a low risk. Table S4 summarizes the basic statistics of the selected variables.

FDA Adverse Events

The next dataset contains the reports submitted to the FDA Adverse Event Reporting System for patients with adverse events [S4]. The binary outcome of interest for this dataset is whether or a patient has died. Our primary goal with this dataset is to explore the relationship between the patient mortality and various predictors, including event date, gender, age, weight, drug name and the indication for drug use. Detailed statistics for these variables can be found in Table S5.

Texas Inpatients (2012)

Texas inpatient dataset [S5] contains 75 variables. Similar to the Washington state hospital discharge data, in this dataset, we explore the relationship between those demographic and health factors and the length of stay in the Texas hospitals. The involved covariates include age, sex, race, ethnicity, location,

weekday, risk mortality, severity, DRG and fees with detailed description in Table S6. According to their length of stay in the hospital, the patients are classified into two groups, and the outcome is assigned with a value of 1 if the patient's length of stay is greater than or equal to 3 days and 0 otherwise.

Washington State Hospital Discharges (2007)

The Washington State Hospital Discharge dataset [S6] contains over 350 variables. Among these, we model the relationship between those demographic and health factors and the length of stay in the hospital. The covariates were: age, atype, aweekend, died, DRG, primary diagnosis code, and ZIP code. A detailed description of these variables is displayed in Table S7. The outcome of our study categories patients into two groups based on their length of stay. A value of 1 is assigned if the patient's length of stay is greater than or equal to 3 days and 0 otherwise.

Basic Stand Alone (BSA) Inpatient Claims

This dataset [S7] contains the claim-level information with each recording being an inpatient claim chosen from a 5% random sample of Medicare beneficiaries during 2008. In this study, we choose the variables including age, gender, DRG, ICD-9 primary procedure code, Medicare payment and the length of stay and explore the relationship between the length of stay and its relevant demographic and claim-related factors. The outcome is defined as a binary variable taking a value of 1 if the length of stay on the file is greater than or equal to 2.5 days, and 0 otherwise. Table S8 provides an overview of the detailed statistics for these variables.

Washington State Hospital Discharges (2008)

This dataset [S6] contains 652,340 inpatient discharge records in 2008 from community hospitals in Washington from State Inpatient Databases that are used to track the trends in healthcare utilization, access, charges, quality and outcomes in United States. We are interested in examining the relationship between length of stay and its demographic and health factors. Specifically, the covariates of interest include age, female, race, admission type, aweekend, DRG, DX1, primary payer, total charges, zip code, chronic conditional indicators, and procedure classes for ICD-10-PCS procedure codes, comorbidity measures for alcohol abuse, depression, hypertension and obesity. The outcome is created by classifying the patients into two groups based on the median of their length of stay. A value of 1 is assigned if the patient's length of stay is greater than or equal to 2 days and 0 otherwise. Detailed statistics of the variables are displayed in Table S9.

California Hospital Discharges (2008)

This dataset [S6] contains over 4 million inpatient discharge records in 2008 from community hospitals in California from State Inpatient Databases that are used to track the trends in healthcare utilization, access, charges, quality and outcomes in United States. We are interested in exploring the relationship between length of stay and its demographic and health factors. Specifically, the covariates of interest include age, female, race, aweekend, DRG, DX1, primary payer, total charges, chronic conditional indicators, and procedure classes for ICD-10-PCS procedure codes, comorbidity measures for alcohol abuse, depression, hypertension and obesity. The outcome is generated by dividing the patients into two groups based on the median of their length of stay. A value of 1 is assigned if the patient's length of stay is greater than or equal to 3 days and 0 otherwise. Detailed statistics of the variables are displayed in Table S10.

Florida Hospital Discharges (2007)

This dataset [S6] contains over 2.3 million inpatient discharge records in 2007 from community hospitals in Florida from State Inpatient Databases that are used to track the trends in healthcare utilization, access, charges, quality and outcomes in United States. We are interested in exploring the relationship between length of stay and its demographic and health factors. Specifically, the covariates of interest

include age, female, race, admission type, aweekend, DRG, DX1, primary payer, total charges and zip code. The outcome is created by classifying the patients into two groups based on the median of their length of stay. A value of 1 is assigned if the patient's length of stay is greater than or equal to 3 days and 0 otherwise. Detailed statistics of the variables are displayed in Table S11.

New York Hospital Discharges (2007)

This dataset [S6] consists of over 4.6 million inpatient discharge records in 2007 from community hospitals in New York from State Inpatient Databases that are used to track the trends in healthcare utilization, access, charges, quality and outcomes in United States. We are interested in examining the relationship between length of stay and demographic and health factors. Specifically, the covariates of interest include age, female, race, admission type, aweekend, DRG, DX1, primary payer, total charges, zip code, chronic conditional indicators and procedure classes for ICD-10-PCS procedure codes. The outcome is created by classifying the patients into two groups based on the median of their length of stay. A value of 1 is assigned if the patient's length of stay is greater than or equal to 3 days and 0 otherwise. Detailed statistics of the variables are displayed in Table S12.

Better Outcomes Registry & Network

Data are collected from BORN Ontario birth registry [S8] that covers about 1 million records regarding Ontario's maternal demographic characteristics, obstetrical history, health behaviors, prenatal screening and newborn care information. We combine the pregnancy and infant datasets and examine the association between low birthweight and its related risk factors. The relevant factors include as gestational age, maternal age, maternal body mass index, total number of pregnancies a mother has experienced, number of previous preterm pregnancies, number of previous abortions, maternal smoking status, alcohol exposure, prenatal screening, mental health concerns for addiction, anxiety, depression, maternal health conditions for diabetes and genetics and drug exposure to Cocaine, Hallucinogens and Opioids. We follow the definition of low birthweight[25] and classify the newborns whose birth weights are less than 2,500 grams as infants with low birthweight. A value of 1 is given for newborns with low birthweight and 0 otherwise. A summary of descriptive statistics for the variables is presented in Table S13.

Medical Information Mart for Intensive Care III

The dataset [S9] is extracted from MIMIC-III relational database (version 1.4), which contains deidentified clinical data of the patients who were admitted to the Beth Israel Deaconess Medical Center in Boston, Massachusetts [S9,S44,S45]. It contains various tables of patient's data regarding the demographics, admission information, lab tests, diagnosis codes, caregiver information, discharge notes. We use this dataset to investigate the relationship between 30-day readmission and its related demographics, vital signs and lab test values. The demographics include the age of the patients when they were first admitted to the ICU, their ethnicity group and admission type. The vital signs consider the (systolic and diastolic) blood pressure, heart rate and respiration rate. Several lab measurements are also incorporated into the analysis. The selection criteria for readmitted patients is to include those who were readmitted within 30-day of initial hospital discharge from the ICU. The patients who were readmitted to the ICU are assigned a label of 1, while those who were not readmitted are assigned a label of 0. Table S14 summarizes the descriptive statistics of the selected variables.

Sensitivity Analysis of Sample Size Calculation to Training Dataset

We investigated the sensitivity of the sample size calculator to the datasets that were used to train it. Tables S15-S18 show the results of the certainty curve sample size estimation performance on our two evaluative metrics, mRE and mALQE, when removing each individual dataset completely from training at different values of lambda and certainty. For example, the CCHS row indicates the performance value when the CCHS is removed from the training datasets. The performance is still calculated using leave-one-dataset-out for the remaining 12 datasets after removing each dataset from training. The default value at the bottom of the table shows the performance value with all of the datasets included in the calculation.

Pseudocode process for the sample size calculator function

The following pseudocode describes the algorithm implanted in the R code found in [S46].

Algorithm: EstimateRequiredSampleSize

Goal:
Estimate the minimum sample size needed to achieve a desired certainty level using a predictive model and smoothed certainty estimates.

Inputs:

- model: a trained predictive model (e.g., LightGBM)
- certainty_threshold: target certainty level (default = 0.8)
- degrees_of_freedom: model complexity indicator
- entropy: average entropy of the data
- imbalance: class imbalance factor

Output:

- required_sample_size: the estimated sample size needed
- achieved_certainty: the model's predicted certainty at that sample size

Steps:

1. **Initialize Parameters**
    - Define a range of candidate sample sizes.
    - Compute their logarithmic values for modeling.
2. **Predict Certainty Across Sample Sizes**
    - Use the model to estimate certainty for each candidate sample size, given fixed values of entropy, imbalance, and degrees of freedom.
3. **Determine Relevant Sample Size Range**
    - If the predicted certainty varies meaningfully:
        - Identify the central region where certainty transitions.
        - Expand this region to include context before and after the transition.
        - Exclude sample sizes that are too small.
    - Otherwise, use the full range of sample sizes.
4. **Refine Certainty Estimates**
    - Recompute certainty predictions over the refined sample size range using a secondary model.

- Apply a smoothing function (e.g., loess) to the logit-transformed certainty values.

5. **Find Minimum Sample Size Meeting Certainty Threshold**
    - Identify the smallest sample size where the smoothed certainty exceeds the threshold.
    - If none found, default to the smallest candidate.
6. **Return Results**
    - Convert the selected log sample size back to its original scale.
    - Return both the required sample size and the corresponding certainty.

References

1. Esri Canada (2023). Canadian health records of COVID-19 gathered by the Public Health Agency of Canada. Available at: https://resources-covid19canada.hub.arcgis.com/

2. Canadian Community Health Survey (2021). Statistics Canada. Available at: https://www150.statcan.gc.ca/n1/pub/82-620-m/2005001/4144189-eng.htm

3. Nexoid (2021). COVID-19 survival dataset. Available at: https://www.covid19survivalcalculator.com/en/download

4. FDA Adverse Event Reporting System (2018). Database comprising information on adverse event and medication error reports submitted to FDA. Available at: https://open.fda.gov/data/faers/

5. Texas Inpatient Public Use Data File (2025). Patient hospital discharge information from Texas hospitals. Available at: https://www.dshs.texas.gov/center-health-statistics/chs-data-sets-reports/texas-health-care-information-collection/health-data-researcher-information/texas-inpatient-public-use

6. Healthcare Cost and Utilization Project (HCUP), Agency for Healthcare Research and Quality (2025). California, Florida, New York, and Washington State Inpatient Databases (SID). Available at: https://hcup-us.ahrq.gov/tech_assist/centdist.jsp

7. Centers for Medicare & Medicaid Services (CMS) (2025). Basic Stand Alone (BSA) Inpatient Claims Public Use File (PUF). Available at: https://www.cms.gov/data-research/statistics-trends-and-reports/basic-stand-alone-medicare-claims-public-use-files/bsa-inpatient-claims-puf

8. Better Outcomes Registry & Network (BORN) Ontario (2021). Data Resource Profile: Better Outcomes Registry & Network (BORN) Ontario. Available at: https://academic.oup.com/ije/article/50/5/1416/6294519

9. Johnson, A.E.W., Pollard, T.J., Shen, L., Lehman, L.H., Feng, M., Ghassemi, M., Moody, B., Szolovits, P., Celi, L.A., and Mark, R.G. (2016). MIMIC-III, a freely accessible critical care database. Scientific Data 3, 160035. Available at: https://doi.org/10.1038/sdata.2016.35

10. Heart Disease (1989). Dataset for predicting heart disease presence. Available at: https://doi.org/10.24432/C52P4X; also available at: https://doi.org/10.17605/OSF.IO/7BS8Q

11. Breast Cancer Wisconsin (1993). Breast cancer diagnostic dataset. Available at: https://doi.org/10.24432/C5DW2B; also available at: https://doi.org/10.17605/OSF.IO/7BS8Q

12. Chronic Kidney Disease (2015). Kidney disease prediction dataset. Available at: https://doi.org/10.24432/C5G020; also available at: https://doi.org/10.17605/OSF.IO/7BS8Q

13. Breast Cancer Coimbra (2018). Diagnostic Coimbra breast cancer dataset. Available at: https://doi.org/10.24432/C52P59; also available at: https://doi.org/10.17605/OSF.IO/7BS8Q

14. Breast Cancer (1988). Breast cancer prediction dataset. Available at: https://doi.org/10.24432/C51P4M; also available at: https://doi.org/10.17605/OSF.IO/7BS8Q

15. EEG Eye State (2013). EEG measurement dataset. Available at: https://doi.org/10.24432/C57G7J; also available at: https://doi.org/10.17605/OSF.IO/7BS8Q

16. Adult (1996). Adult income classification dataset. Available at: https://doi.org/10.24432/C5XW20; also available at: https://doi.org/10.17605/OSF.IO/7BS8Q

17. Bank note (2012). Bank note dataset. Available at: https://doi.org/10.24432/C55P57; also available at: https://doi.org/10.17605/OSF.IO/7BS8Q

18. Titanic Survival (1912). Titanic survival prediction dataset. Available at: https://www.kaggle.com/datasets/hesh97/titanicdataset-traincsv; also available at: https://doi.org/10.17605/OSF.IO/7BS8Q

19. Stroke (2023). Stroke Prediction dataset. Available at: https://doi.org/10.17605/OSF.IO/7BS8Q

20. Colposcopy/green (2017). Digital Colposcopy (green) dataset. Available at: https://doi.org/10.24432/C5C022; also available at: https://doi.org/10.17605/OSF.IO/7BS8Q

21. Colposcopy/hinselmann (2017). Digital Colposcopy (hinselmann) dataset. Available at: https://doi.org/10.24432/C5C022; also available at: https://doi.org/10.17605/OSF.IO/7BS8Q

22. Colposcopy/schiller(2017). Digital Colposcopy (schiller) dataset. Available at: https://doi.org/10.24432/C5C022; also available at: https://doi.org/10.17605/OSF.IO/7BS8Q

23. Thoracic Surgery (2014). Post-operative life expectancy classification prediction dataset. Available at: https://doi.org/10.24432/C5Z60N; also available at: https://doi.org/10.17605/OSF.IO/7BS8Q

24. Diabetic Retinopathy Debrecen (2014). Diabetic retinopathy prediction dataset. Available at: https://doi.org/10.24432/C5XP4P; also available at: https://doi.org/10.17605/OSF.IO/7BS8Q

25. Z-Alizadeh Sani (2013). Coronary artery disease prediction dataset. Available at: https://doi.org/10.24432/C5Q31T; also available at: https://doi.org/10.17605/OSF.IO/7BS8Q

26. Pima Indians Diabetes (1988). Diabetes dataset from Pima Indians. Available at: https://doi.org/10.17605/OSF.IO/7BS8Q

27. Figueroa, R.L., Zeng-Treitler, Q., Kandula, S., and Ngo, L.H. (2012). Predicting sample size required for classification performance. BMC Med Inform Decis Mak *12*, 8. Available at: https://doi.org/10.1186/1472-6947-12-8.

28. Mukherjee, S., Tamayo, P., Rogers, S., Rifkin, R., Engle, A., Campbell, C., Golub, T.R., and Mesirov, J.P. (2003). Estimating Dataset Size Requirements for Classifying DNA Microarray Data. Journal of Computational Biology *10*, 119–142. Available at: https://doi.org/10.1089/106652703321825928.

29. Raudys, S.J., and Jain, A.K. (1990). Small sample size effects in statistical pattern recognition: recommendations for practitioners and open problems. In [1990] Proceedings. 10th International Conference on Pattern Recognition (IEEE Comput. Soc. Press), pp. 417–423. Available at: https://doi.org/10.1109/ICPR.1990.118138.

30. Fukunaga, K., and Hayes, R.R. (1989). Effects of sample size in classifier design. IEEE Trans. Pattern Anal. Machine Intell. *11*, 873–885. Available at: https://doi.org/10.1109/34.31448.

31. Larracy, R., Phinyomark, A., and Scheme, E. (2021). Machine Learning Model Validation for Early Stage Studies with Small Sample Sizes. In 2021 43rd Annual International Conference of the IEEE Engineering in Medicine & Biology Society (EMBC) (IEEE), pp. 2314–2319. Available at: https://doi.org/10.1109/EMBC46164.2021.9629697.

32. van der Ploeg, T., Austin, P.C., and Steyerberg, E.W. (2014). Modern modelling techniques are data hungry: a simulation study for predicting dichotomous endpoints. BMC Medical Research Methodology *14*, 137. Available at: https://doi.org/10.1186/1471-2288-14-137.

33. Vabalas, A., Gowen, E., Poliakoff, E., and Casson, A.J. (2019). Machine learning algorithm validation with a limited sample size. PLOS ONE *14*, e0224365. Available at: https://doi.org/10.1371/journal.pone.0224365.

34. McNamara, M.E., Zisser, M., Beevers, C.G., and Shumake, J. (2022). Not just “big” data: Importance of sample size, measurement error, and uninformative predictors for developing prognostic models for digital interventions. Behaviour Research and Therapy *153*, 104086. Available at: https://doi.org/10.1016/j.brat.2022.104086.

35. Zantvoort, K., Nacke, B., Görlich, D., Hornstein, S., Jacobi, C., and Funk, B. (2024). Estimation of minimal data sets sizes for machine learning predictions in digital mental health interventions. npj Digit. Med. *7*, 1–10. Available at: https://doi.org/10.1038/s41746-024-01360-w.

36. Acharjee, A., Larkman, J., Xu, Y., Cardoso, V.R., and Gkoutos, G.V. (2020). A random forest based biomarker discovery and power analysis framework for diagnostics research. BMC Med Genomics *13*, 178. Available at: https://doi.org/10.1186/s12920-020-00826-6.

37. D’souza, R.N., Huang, P.-Y., and Yeh, F.-C. (2020). Structural Analysis and Optimization of Convolutional Neural Networks with a Small Sample Size. Sci Rep *10*, 834. Available at: https://doi.org/10.1038/s41598-020-57866-2.

38. Bartlett, P.L., Harvey, N., Liaw, C., and Mehrabian, A. (2017). Nearly-tight VC-dimension and pseudodimension bounds for piecewise linear neural networks. Preprint at arXiv, https://arxiv.org/abs/1703.02930

39. Beleites, C., Neugebauer, U., Bocklitz, T., Krafft, C., and Popp, J. (2013). Sample size planning for classification models. Analytica Chimica Acta *760*, 25–33. Available at: https://doi.org/10.1016/j.aca.2012.11.007.

40. Balki, I., Amirabadi, A., Levman, J., Martel, A.L., Emersic, Z., Meden, B., Garcia-Pedrero, A., Ramirez, S.C., Kong, D., Moody, A.R., et al. (2019). Sample-Size Determination Methodologies for Machine Learning in Medical Imaging Research: A Systematic Review. Can Assoc Radiol J *70*, 344–353. Available at: https://doi.org/10.1016/j.carj.2019.06.002.

41. Azizi, Z., Lindner, S., Shiba, Y., Raparelli, V., Norris, C.M., Kublickiene, K., Herrero, M.T., Kautzky-Willer, A., Klimek, P., Gisinger, T., et al. (2023). A comparison of synthetic data generation and federated analysis for enabling international evaluations of cardiovascular health. Sci Rep *13*, 11540. Available at: https://doi.org/10.1038/s41598-023-38457-3.

42. Lloyd-Jones, D.M., Hong, Y., Labarthe, D., Mozaffarian, D., Appel, L.J., Van Horn, L., Greenlund, K., Daniels, S., Nichol, G., Tomaselli, G.F., et al. (2010). Defining and Setting National Goals for Cardiovascular Health Promotion and Disease Reduction. Circulation *121*, 586–613. Available at: https://doi.org/10.1161/CIRCULATIONAHA.109.192703.

43. Maclagan, L.C., Park, J., Sanmartin, C., Mathur, K.R., Roth, D., Manuel, D.G., Gershon, A., Booth, G.L., Bhatia, S., Atzema, C.L., et al. (2014). The CANHEART health index: a tool for monitoring the cardiovascular health of the Canadian population. CMAJ *186*, 180–187. Available at: https://doi.org/10.1503/cmaj.131358.

44. Goldberger, A.L., Amaral, L.A.N., Glass, L., Hausdorff, J.M., Ivanov, P.Ch., Mark, R.G., Mietus, J.E., Moody, G.B., Peng, C.-K., and Stanley, H.E. (2000). PhysioBank, PhysioToolkit, and PhysioNet: Components of a New Research Resource for Complex Physiologic Signals. Circulation *101*. Available at: https://doi.org/10.1161/01.CIR.101.23.e215.

45. Johnson, A., Pollard, T., and Mark, R. (2016). MIMIC-III Clinical Database (version 1.4). (PhysioNet). Available at: https://doi.org/10.13026/C2XW26.

46. Mitsakakis, N., Liu, D., and El Emam, K. Sample Size Calculation for Training Ensemble Machine Learning Models on Health Data. OSF. Available at: https://doi.org/10.17605/OSF.IO/7BS8Q
